# Investigation of the effect of outflow from a sharp bend, influenced by a pipe installed at the bend exit, on the scour pattern in the downstream straight reach under unsteady flow conditions

**DOI:** 10.1371/journal.pone.0360062

**Published:** 2026-10-27

**Authors:** Elaheh Barzegarpisheh, Mohammad Vaghefi, Seyed Hamed Meraji

**Affiliations:** Department of Civil Engineering, Persian Gulf University, Bushehr, Iran; Tishk International University, IRAQ

## Abstract

A major threat to the stability of pipes crossing rivers at width is scouring around such structures. Pipe failure and the consequent serious damage to and life-threatening cutoff of vital streams are possible outcomes of this phenomenon. Although numerous studies have investigated scour around pipes in straight channels and bends, changes in bed topography along the straight reach downstream of a sharp bend, which is affected by the complex flow pattern issuing from the bend, have received less attention. Therefore, this study examines the effect of pipe installation at the end of a sharp bend and its influence on the downstream bed topography along the straight reach. In addition, from both an operational and hydraulic perspective, placing the pipe outside the bend would be a preferable option for designers and engineers. The study investigated, among others, the pipe position relative to the bed level as well as the hydrographic parameters in terms of flow unsteadiness and the effect these could have on the pattern of scouring downstream of the 180-degree sharp bend. The experiments were conducted under three pipe-installation conditions relative to the initial bed level, namely level with the bed, above the bed, and buried, with the pipe positioned perpendicular to the flow direction and under four different ratios of hydrograph peak timing to flood duration in unsteady flow. To evaluate the effects of different pipe installation conditions as well as the flow hydrograph, parameters such as the extent of scour-hole development, the maximum scour depth, the maximum deposition height, and the bed topography changes in the straight reach downstream of the bend were measured and analyzed. The main focus of the research was toward identification of the scour hole development and the maximum scour depth variations and how this phenomenon interacted with the stated parameters. The study findings indicated that the most critical case of bed topography variations under the influence of pipe occurred when the pipe was in a level position with the bed, where altering the pipe level from bed level to buried levels decreased the maximum scour and sedimentation by respectively 37.1% and 38.0%. Furthermore, alteration of unsteady-flow related parameters had less effect toward scour reduction than that of pipe level position with respect to the initial bed level. The findings of this study can be used to identify the most suitable pipe installation condition in the straight reach downstream of sharp bends, reduce scour risk, predict bed changes, and improve the design and protection of pipelines in river engineering projects.

## 1. Introduction

Pipelines as vital streams aimed for transferring fluids such as water, oil and gas, are occasionally and inevitably crossing rivers at width. Since most rivers are naturally meandering and consisting of bends, complex flow patterns at such sections could result in complicated interactions between the flowing water and the riverbed, hence intensifying the process of sedimentation and scouring beneath these pipes. Such a phenomenon could decrease stability and lead to pipe structure failure or damage, and considerable environmental and economical outcomes. Special attention is therefore needed to understand the effect of river bend geometry and flow conditions on the scour pattern around pipes for secure and stable designing of transmission pipelines. For better understanding and due to the significance of the issue, a literature review was conducted in this regard.

River bends play a determining role in flow behavior as they represent geometric variations in flow path. This alteration in the path results in flow diversion, non-unified velocity and pressure distribution and formation of complicated flow patterns, all of which have considerable effects on flow hydraulic properties. The prior research on the effects of bends on flow pattern was first to be reviewed. Vaghefi et al. (2016) studied the effect of variations in streamlines, maximum velocity distribution and the secondary flow power on bed shear stress distribution in a sharp 180-degree bend [1]. In a later laboratory study, Akbari and Vaghefi (2017) focused on streamlines in a sharp 180-degree and found that as the flow approached the bend apex, the power of the secondary flow as well as the size of the vortices increased [2]. Sui et al. (2006), Biswas and Barbhuiya (2020) investigated scour in river bends with 90- and 180-degree angles and, in their studies, examined the parameters affecting scour as well as changes in the bed topography of the bend [3, 4].

Flows, in terms of temporal variability, are classified into steady and unsteady types. Given the temporal variations in flow depth and velocity inherent to the unsteady flow type, the present study focused on examining this class of flows. Accordingly, previous studies and published works related to the behavior of unsteady flows and their effects on flood management and bed changes are reviewed in the following sections. Salamatian et al. (2014) experimentally investigated sediment transport under unsteady flow conditions in a straight laboratory flume [5]. Zarrati and Tabarestani (2017) conducted an experimental study on local scour around a bridge pier under flooding flow conditions, with particular emphasis on steep hydrographs, in a straight laboratory flume [6]. Wang et al. (2019) In this experimental study, the evolution of bed morphology due to bed load transport under unsteady hydrograph flows was investigated. The results indicate that the magnitude and unsteadiness of the hydrograph have significant impacts on sediment transport rates and yields, as well as on the hysteresis patterns and yield ratios generated during the rising and falling limbs of the hydrograph [7]. Solati et al. (2021) experimented on unsteady flow, investigating the effects of peak discharge timing and flooding duration on local scour around a bridge pier in a sharp bend [8]. Cahyani et al. (2022) a study was conducted on the Cengkareng Floodway to investigate the impact of sediment transport on bed morphological changes. Utilizing a Quasi-Unsteady Flow model within the HEC-RAS 1D software and accounting for tidal boundary conditions, the researchers simulated the sedimentation rates [9]. Solati et al. (2023) carried out an experimental study in a 180-degree bend to study the effects of unsteady flows on scour around bridge piers with different shapes and positions [10]. Mananoma et al. (2025) Regarding the management of flood flows and sediment transport, the researchers designed a multi-stage channel profile using HEC-HMS and HEC-RAS models to control flooding and mitigate the adverse impacts of sediment movement [11]. Kusmajaya et al. (2025) Regarding flood risk management, this study investigates the optimization of Limboto Lake operations as a flood retention basin. By employing optimization techniques and simulating various operational scenarios for the Tapodu Canal gates, the research demonstrates that appropriate gate regulation can significantly reduce peak discharge during high-return-period flood events, effectively preventing overflow in the downstream canal [12]. Given that this subsection focuses on the behavior of unsteady flows, sediment transport, and bed changes, the studies related to scour around pipelines under these conditions have been reviewed separately in the next section in order to maintain coherence.

The presence of a pipe directly affects flow behavior and plays a significant role in altering flow hydraulics and bed topography. As the present study focused on assessing the influence of pipe presence on downstream bed topographic variations in a channel bend, relevant previous studies on flow characteristics in the presence of pipes are reviewed in the following section. Dey and Singh (2008) investigated the maximum scour depth beneath a pipeline in a rectangular channel under clear-water and steady-flow conditions, using both uniform and non-uniform sediments. Their results indicated that the equilibrium scour depth increased up to a certain limit when increasing approach flow depth, beyond which further increases in flow depth had no significant effect on the equilibrium scour depth. Moreover, they found that as sediment gradation became more non-uniform, the scour depth was substantially reduced due to the formation of an armor layer within the scour hole [13]. In a later experimental study in a rectangular channel, Wu and Chiew (2013) investigated the scour hole and the flow field around a pipeline under steady-flow conditions. They concluded that the forces responsible for sediment motion and transport were vortices generated by the pressure difference between the upstream and downstream regions of the pipeline [14]. Cheng et al. (2014) conducted an experimental study in a straight flume to investigate the scour process around pipelines under one-way flow, wave-only, and combined wave–flow conditions. They examined the effects of parameters such as flow velocity, the angle of streamlines incidence relative to the pipeline, and the initial depth of the pipeline with respect to the bed. The results demonstrated that increasing the initial depth of the pipeline significantly reduced the extent of scour [15]. Azamathulla et al. (2014) experimented in a rectangular concrete flume to investigate the effect of the angle of flow incidence relative to a pipeline on the scour process. Their results showed that changing the orientation angle of the pipeline with respect to the flow direction caused the scour hole to shift toward the channel wall. The results also indicated that variations in the pipeline orientation angle had no significant effect on the maximum scour depth [16]. Based on combined experimental and numerical investigations, Zhao et al. (2015) showed that in a configuration of two consecutive pipelines with varying spacings, the scour depth beneath the upstream pipeline in a mobile bed was slightly greater than that of a single pipeline, whereas the scour depth beneath the downstream pipeline increased markedly. They also reported that the scour depth under clear-water conditions was greater than that in a live-bed regime, due to the absence of sediment replenishment from the upstream region. Moreover, reducing the spacing between the two pipelines led to a decrease in the maximum scour depth [17]. In their numerical study, Postacchini et al. (2015) examined the effect of bed sediment cohesion on the scour phenomenon beneath a pipeline [18]. Parsaie et al. (2019) employed a Support Vector Machine (SVM) model to predict the scour depth around pipelines crossing rivers. Their findings indicated that the parameters τ (bed shear stress), e/D (relative roughness), and Fr (Froude number) exerted the most significant influence on the intensity of this phenomenon [19]. In an experimental study, Bijanvand et al. (2021) investigated scour around pipelines crossing river flow under unsteady flow conditions in a straight channel. The experiments were conducted using three pipe diameters and three installation configurations. The results showed that the final scour hole depth under unsteady flow conditions was smaller than that under steady flow, and that installing the pipeline at a height equal to one-quarter of the pipe diameter above the bed led to an increase in scour around the pipeline [20]. Li and Ma (2022) examined the scour mechanisms around submarine pipelines in suspended and semi-buried conditions. The results indicated that the intensity and pattern of local scour were influenced by factors such as the Froude number, pipe diameter, distance from the bed, and installation depth. Semi-buried or fully buried pipeline installations generally led to an increase in the surrounding scour depth. In addition, the pipeline elevation and curvature played a significant role in shaping the flow field and scour pattern, such that increasing the distance between the pipeline and the bed enhanced the flow velocity above the pipeline and intensified downstream scour [21]. Kolur et al. (2022) investigated the effects of diameter, arrangement, and number of pipelines on the depth and pattern of scour in a rectangular channel. Their findings revealed that in pipelines of identical diameter, the maximum scour occurred between the pipes, while in pipelines of varying diameters, the largest scour formed around the larger pipe. Positioning the larger pipe upstream reduced the scour depth, and a descending diameter arrangement was identified as the optimal configuration [22]. Valizadeh et al. (2023) experimentally investigated the effects of pipe orientation and the use of an apron on the scour and sedimentation patterns around a semi-buried pipeline in a mild 90-degree bend. The results showed that the crossing angle of the pipeline in the river bend significantly altered the scour pattern; the vertical configuration produced the greatest downstream scour, whereas the repelling configuration generated the maximum scour beneath the pipeline [23]. Hu et al. (2023) numerically investigated local scour around submarine pipelines using two‑dimensional hydrodynamic and sediment‑transport models. The simulations showed that bed shear stress was the primary driver of scouring, and that factors such as flow velocity, sediment grain size, pipe diameter, and the initial pipe–bed distance exerted significant influence on the equilibrium scour depth and the morphology of the scour hole surrounding the pipeline [24]. Karamzadeh et al. (2024) examined the effect of protective blades on the depth and length of the scour hole beneath a submerged pipeline in a rectangular flume and proposed predictive relationships for these parameters. The experimental results demonstrated that the position and length of the blade, particularly for small‑diameter pipelines, played a significant role in reducing scour depth [25]. Li and Dziedzic (2025) experimentally investigated the scour behavior around a pipeline crossing a river under different flow conditions, pipe configurations, and ice conditions. The results indicated that ice cover increased the equilibrium scour depth and reduced the time required to reach equilibrium. In contrast, multi-pipe layouts reduced the maximum scour depth but accelerated scour onset [26]. Yang and Yang (2026) investigated experimentally the local scour caused by a pipeline crossing the width of a river. The results showed that the rate of scour hole expansion depends on the flow velocity and the water depth. In addition, as the pipe diameter increases, the number of scour holes formed beneath the pipeline decreases, and the locations of these holes along the pipeline axis become more stable [27].

Studies conducted in straight channels have clearly demonstrated the role of hydraulic and geometric factors in the development of scour around pipelines; however, the conditions prevailing in river bends are more complex due to the formation of secondary currents induced by centrifugal force and changes in the distribution of velocity and shear stress. These processes cause lateral sediment transport and, consequently, alter the scour and deposition patterns compared with straight channels. The downstream reaches of sharp river bends are among the most morphodynamically sensitive zones, because the flow exiting the bend retains part of its three-dimensional structure, affects the hydrodynamic and morphodynamic behavior of the bed in this region, and increases the likelihood of scour around hydraulic structures. Therefore, generalizing the results of studies conducted in straight channels to this region is limited, and the independent investigation of this reach, particularly in the presence of crossing pipelines, is of special importance. A review and comparison of previous studies indicates that, although the role of factors such as hydraulic conditions, sediment characteristics, and pipe installation conditions in the initiation and development of scour around pipelines is well recognized, most studies have examined these effects independently. For example, some studies have focused on the influence of pipe burial depth or installation configuration, others on hydraulic parameters such as flow velocity, Froude number, and the angle of flow incidence on the pipe, and only a limited number have addressed unsteady flow conditions. Moreover, most of these studies have been conducted in straight channels or within the bend itself, while the morphodynamic behavior of the bed in the straight reach downstream of the bend, which is directly affected by the flow issuing from the bend, has received less attention. Therefore, despite a sound understanding of the independent effects of the factors influencing scour, available information on the combined effects of unsteady flow conditions and different pipe installation configurations on scour development and bed topographic changes in this region remains limited. Accordingly, these two factors were selected as the main variables of the present study to evaluate their interactive effects on topographic changes in the straight reach downstream of the bend. The novelty of this study lies in providing an integrated experimental assessment of the morphodynamic response of the bed in the straight reach downstream of a 180-degree sharp bend in the presence of a crossing pipeline under unsteady flow conditions. In this study, the simultaneous effects of different pipe installation conditions and different peak-flow timing ratios in the unsteady-flow hydrograph on scour development, deposition pattern, and bed topographic changes were experimentally investigated. This approach enables a better understanding of bed behavior under conditions closer to those found in natural rivers and can provide a basis for safer design, placement, and management of crossing pipelines downstream of sharp bends.

## 2. Materials and methods

The Materials and Methods section consists of five subsections.

In Section 2.1, the experimental setup is described. Section 2.2 presents the characteristics and size of the bed particles, as well as the installation and fixation of the pipe in the laboratory flume. Section 2.3 explains the experimental procedure and the operational steps. Section 2.4 describes the post-experiment data collection procedure and the method used to record the observations. Finally, Section 2.5 presents the experiment naming convention and the format of the experiment identifiers.

### 2.1. Description of the setup

The experiments were conducted in a laboratory flume incorporating a 180-degree bend with a central curvature radius of 2 m, classified as a sharp bend. The flume consists of a 6.5 m upstream reach and a 5.1 m downstream reach, which are connected by the bend with an inner radius of 1.5 m and an outer radius of 2.5 m. The channel width is 1 m, and the flume bed is made of metal, while the sidewalls are constructed of glass with a height of 0.7 m to allow visual observation of the flow. In addition, a 25 m³ metal reservoir was installed beneath the flume to supply and recirculate water within the system. A schematic view of the laboratory flume is presented in Fig 1.

**Fig 1:**
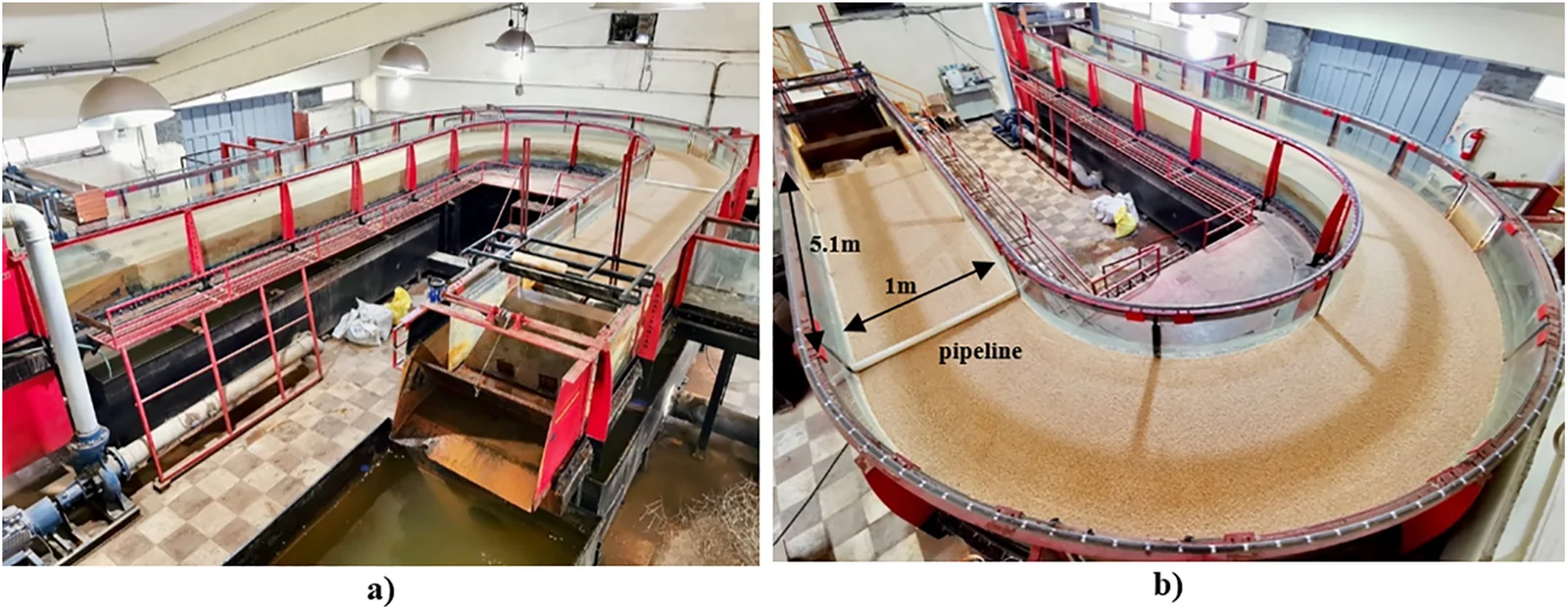
View of the laboratory flume: (a) in front view, and (b) plan.

To reduce flow turbulence and to homogenize the inflow to the channel, a flow straightener was installed at the channel inlet. This device consisted of two frames made of multilayer fine-mesh aluminum screens placed in the path of the flow discharged from the pump. As a result, the flow entered the straight upstream reach of the channel with reduced turbulence, and after passing through this straight reach with minimum disturbance and turbulence, it entered the bend. Along the upstream bend, the flow conditions changed due to the formation of secondary flow, its interaction with the longitudinal flow, and the development of a helical flow pattern.

### 2.2. Description of the bed and pipe installation

Given that preliminary experiments showed noticeable changes in the bed topography up to 1.5 m downstream of the end of the bend, the bed topography along the straight downstream reach was measured up to 2 m to investigate the effect of the interaction between the flow exiting the bend and the pipe oriented perpendicular to the unsteady flow path. the pipeline was placed at the terminal angle of the bend, corresponding to the beginning of the downstream straight reach. The selection of this pipeline location was motivated by two main objectives. Given that under real-world and practical conditions, efforts are typically made to avoid placing pipeline–river crossings within bends, by choosing this position, the pipeline was located not only at the terminal part of the bend but also effectively within the straight reach. Therefore, this configuration allowed examination of the maximum influence of the secondary and helical flows generated within the bend on the pipeline installed at the beginning of the downstream straight reach. Further, to model the pipeline, a polycarbonate pipe with a diameter of 5 cm was installed in a streamwise direction under three different burial conditions: level with the bed surface, elevated by one pipe diameter above the bed, and buried within the bed material. With regard to selecting the installation level of the pipe perpendicular to the unsteady flow relative to the initial bed, the buried condition represents the conventional method of protecting pipelines by embedding them within the bed, thereby reducing direct flow–pipe interaction. The level with the bed condition refers to the situation in which scour around the pipe has progressed to a depth equal to the pipe diameter; in other words, the pipe has become exposed at the level of the riverbed over time, or, due to construction constraints, it has been installed flush with the bed. The above the bed condition indicates a state in which severe flooding has caused deep scour around the pipe, and in subsequent floods the pipe is positioned above the initial bed. In this condition, part of the flow passes beneath the pipe and part passes over it, while the pipe itself acts as an obstacle in the flow path. Since each of these three pipe-installation conditions relative to the initial bed affects the bed differently under unsteady flow, these three installation levels were selected relative to the initial bed. Before each experiment, the pipe was positioned at the desired distance from the bed, perpendicular to the flow direction and across the channel width. To ensure a constant pipe clearance from the initial bed, a level gauge was used. After verifying the correct alignment, the pipe was fixed to both channel walls using transparent aquarium sealant. The sealant was applied carefully so as not to induce flow distortion or additional roughness effects, and thus to prevent any wall-induced deflection of the flow. Throughout the experiments, the pipe remained completely stationary, with no vibration or displacement, and behaved as a rigid body oriented perpendicular to the flow. In addition, a mechanical bed-leveling device was used to prepare the initial bed. This device was mounted on rails installed along the top of the channel walls, with the same slope as the longitudinal bed slope of the channel. At the start of each test, the device was moved along the channel to produce an initial bed with a uniform longitudinal slope. Fig 2 illustrates the different installation configurations of the pipe relative to the bed level.

**Fig 2:**
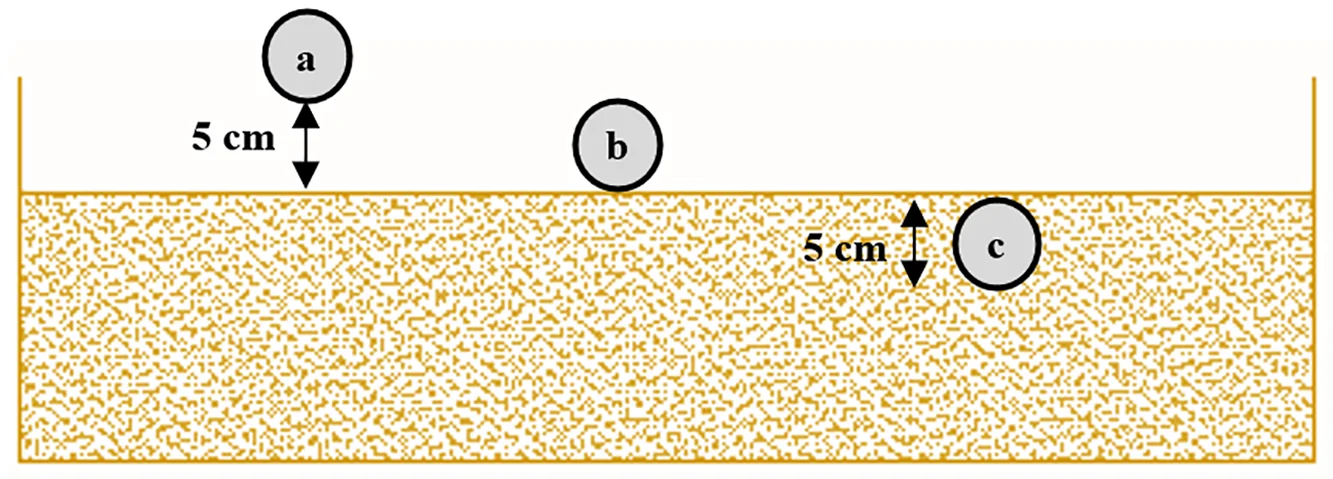
Schematic of the different pipeline installation configurations relative to the bed level.

In these experiments, the bed material consisted of silica sand with a median grain size of 1.5 mm. According to Melville and Chiew (1987), if the standard deviation of the bed material particles is less than 1.3, the effect of particle non-uniformity on scour development is negligible. In the present study, the standard deviation of the bed particles was 1.14. Therefore, these materials fall within a uniformity range in which armoring and selective transport effects are minimized [28].

### 2.3. Experimental procedure

To evaluate the effect of the hydrograph peak time on the scour process around the pipeline, the experiments were conducted with a total duration of 100 minutes and under four different rising‑limb ratios of the hydrograph (0.25, 0.50, 0.75, and 1.00 times the total duration), based on previous studies on scour around hydraulic structures [8, 10]. In all four hydrographs used in these experiments, the base discharge, peak discharge, and duration time were kept constant, and the only variable parameter was the timing of the peak discharge relative to the duration time. This parameter was defined by four different ratios in order to investigate the role of the timing of the peak discharge relative to flood duration in the scour and deposition process. In the hydrograph with Tr/Td = 0.25, the discharge reaches its peak during the first quarter of the experiment and then gradually returns to the base discharge, representing a flood with a sudden peak under real conditions. In the hydrograph with Tr/Td = 0.50, the rising and falling limbs are equal, and the peak discharge occurs at mid-duration, representing an approximately normal flood. In the hydrograph with Tr/Td = 0.75, the discharge reaches its peak after three quarters of the experimental duration and then returns to the base discharge, representing a gradual flood with a delayed peak. In the hydrograph with Tr/Td = 1.0, the discharge increases gradually from the base discharge to the peak discharge, representing the rise of rainfall to its maximum followed by its abrupt cessation.

In all experiments, by keeping the start time of each test consistent relative to the previous test, the pre-wetting conditions of the bed were identical. The experiments were conducted under unsteady flow conditions, with the discharge gradually altered within the range of 25–60 L/s. The water height in the flume was regulated using the tailgate, whose position was adjusted such that for discharge rates lower than 42.5 L/s, the bed shear stress values remained below the critical value, thereby ensuring clear-water conditions. At a discharge rate of 42.5 L/s, the incipient motion of the sediment particles was reached. With further increases in discharge, the bed transitioned into a live-bed condition, in which scour, sediment transport, and sedimentation processes occurred simultaneously.

### 2.4. Bed measurement method

After the 100-minute test duration and subsequent flume drainage, the downstream bed topography of the bend was collected on a predefined grid. The resulting data were then processed and analyzed.

The bed topography was measured using a laser distance meter with an accuracy of 1 mm. The laser meter was mounted on a movable rail system, which allowed systematic displacement of the instrument along the longitudinal and transverse directions of the channel. For data acquisition, the bed was discretized into a grid with transverse spacing of 2.5 cm and longitudinal spacing of 5 cm. In addition, in the vicinity of the pipe and at locations where the topographic variations were more pronounced, the longitudinal spacing was reduced to 2.5 cm or less. To calibrate the measurements and ensure their accuracy before the start of each experiment, the initially flat and leveled channel bed was taken as the reference surface, and the distance from the laser meter to the initial bed surface was taken as the zero-reference level. After each test, the vertical distance between the laser meter and the bed surface was recorded at each grid point, and bed topography changes were calculated based on the difference from the initial zero-reference level. To avoid light refraction effects, all measurements were performed after complete drainage of the bed. In addition, measurements were repeated at several points in each experiment to ensure the repeatability of the measuring locations.

### 2.5. Test naming convention

The experiments were named based on two parameters: the burial depth and the ratio of the hydrograph discharge peak time to the total hydrograph duration. The tests are denoted by the symbol $T_{pipe\;position\;relative\;to\;the\;bed-hydrograph\;ratio}$. The pipe position relative to the bed was classified using three letters: a, b and c. Letter a represents the pipe installation above the bed level, b denotes the pipe installation level with the bed surface, and c is the pipe installation below the initial bed level, i.e., in a buried condition.

## 3. Results and discussion

The Results and Discussion section is divided into five subsections.

In Section 3.1, the maximum scour and deposition depths, as well as their longitudinal positions relative to the pipe installation point, are examined. Section 3.2 analyzes the bed topography under different pipe installation conditions and hydrographs. Section 3.3 presents and interprets the cross-sectional profiles, while Section 3.4 presents and interprets the longitudinal profiles of the bed downstream of the pipe. Finally, Section 3.5 is devoted to the statistical analysis of the data.

### 3.1. Investigation of the maximum scour and sedimentation points

Table 1 presents the maximum scour depth and sedimentation for the pipeline installed at the beginning of the downstream straight reach of the 180-degree bend under three burial conditions: placed level with the bed surface, elevated by one pipe diameter above the bed, and buried by one pipe diameter within the bed material. These results were reported for four different ratios of peak discharge time to flooding duration (0.25, 0.50, 0.75, and 1.00). It should be noted that all of the reported values were nondimensionalized with respect to the pipe diameter (5 cm).

**Table 1:** Non-dimensional maximum scour depth and sedimentation height and their locations.

installation Configuration of pipe	T_r_/T_d_	The title of the experiment	d_s_			h_s_		
**d**_**s max**_ **/D**	**X/D**	**Y/D**	**h**_**s max**_ **/D**	**X/D**	**Y/D**
**Above** **the bed level**	**0.25**	**T**_**a-0.25**_	1.70	0.50	1.50	0.98	1.50	29.0
	**0.50**	**T**_**a-0.50**_	1.74	0.50	1.50	1.0	3.50	30.0
	**0.75**	**T**_**a-0.75**_	1.42	0.0	0.50	0.98	1.0	29.0
	**1.0**	**T**_**a-1.0**_	1.66	0.0	1.50	1.0	0.0	29.0
**bed-level**	**0.25**	**T**_**b-0.25**_	1.82	0.50	4.0	0.96	2.0	25.0
	**0.50**	**T**_**b-0.50**_	1.78	0.50	3.50	1.0	0.0	25.0
	**0.75**	**T**_**b-0.75**_	1.80	0.0	1.0	0.98	0.0	26.0
	**1.0**	**T**_**b-1.0**_	1.86	0.50	1.50	1.14	0.0	31.0
**buried**	**0.25**	**T**_**c-0.25**_	1.24	2.0	4.0	0.62	2.0	30.0
	**0.50**	**T**_**c-0.50**_	1.12	3.50	3.50	0.62	1.50	31.0
	**0.75**	**T**_**c-0.75**_	0.96	4.0	4.0	0.62	1.0	29.0
	**1.0**	**T**_**c-1.0**_	1.24	4.0	4.0	0.66	0.50	28.0

According to Table 1, the scour depth in the configuration where the pipe was placed level with the bed is greater than that in the other pipe installation conditions for all ratios of peak discharge time to flooding duration. Among the experiments, the Tb-1.0 case exhibited the largest maximum scour depth as well as the greatest sedimentation compared to the other tests. Positioning the pipe level with the bed induced severe disruption in the flow field resulting from the direct impact of the bend’s outflow on the pipeline. This increased shear stresses, which consequently exacerbated scouring and reduced bed stability around the pipe.

In contrast, the smallest maximum scour depth and sedimentation corresponded to the buried pipe configuration, which could be attributed to the protection of the pipe against direct flow impingement over most of the experimental duration. The smallest maximum scour depth was observed in the Tc-0.75 test, while the smallest maximum sedimentation occurred at the three ratios of discharge peak time to flooding duration (0.25, 0.50, and 0.75), remaining nearly constant across these conditions. In this configuration, due to the indirect interaction between the flow and the structure, flow turbulence intensity was reduced, and the bed shear stresses did not reach levels sufficient to cause widespread detachment of sediment particles. Consequently, more limited local scour and sedimentation occurred. When the pipe was positioned above the bed, the direct impact of the flow on the pipe surface increased scour compared to the buried configuration; however, because a portion of the flow passed beneath the pipe, the intensity of scour was lower than in the case where the pipe was level with the bed. Accordingly, this installation condition exhibited intermediate levels of scour and sedimentation relative to the other two configurations.

In 70% of the experiments, the maximum transverse scour depth occurred adjacent to the outer bank of the channel, which is consistent with the findings of Vaghefi et al. (2016) for a 180-degree sharp bend. They stated that, with the development of vortices induced by secondary flow and the gradual shift of the velocity core and bed shear stress concentration along the bend, the dominant zone of shear stress concentration in the downstream end of the bend shifts from the inner bank toward the outer bank [1]. Therefore, the occurrence of maximum scour in these regions can be attributed to the persistence of the secondary flow exiting the bend in this area, namely the downstream straight reach.

Fig 3 illustrates the maximum scour and its location along the downstream straight reach of the 180-degree bend for different pipeline installation conditions relative to the initial bed level and for various ratios of the parameters of an unsteady flow.

**Fig 3:**
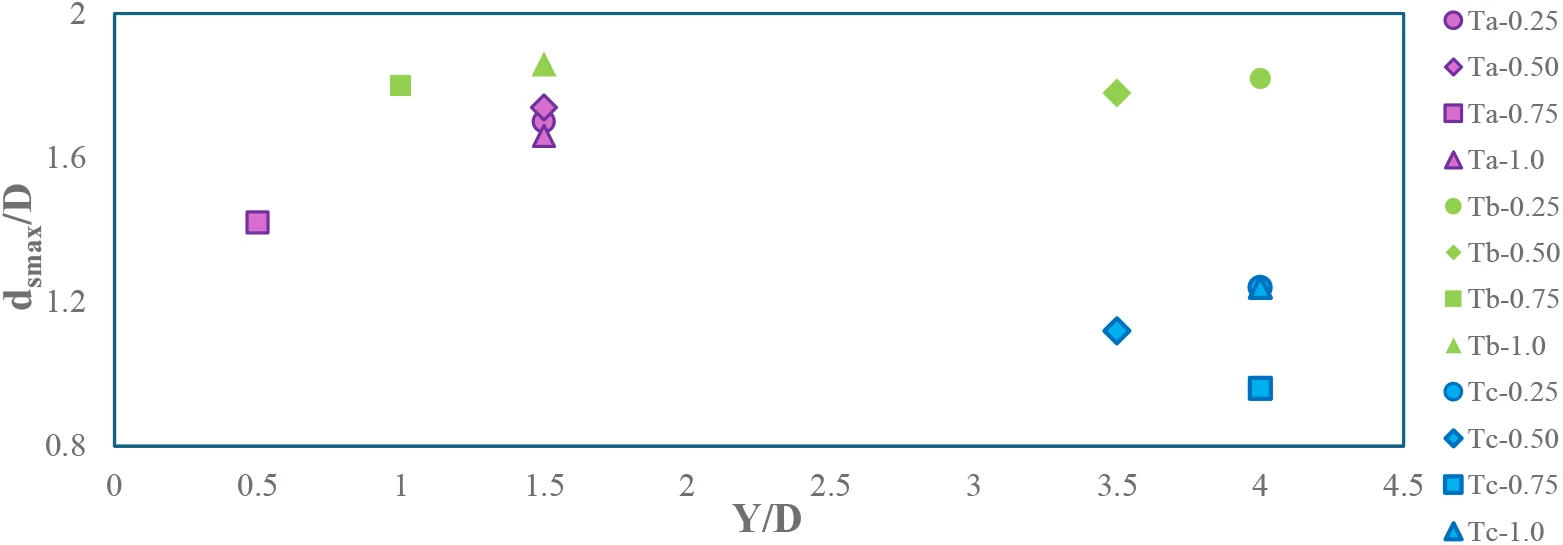
Dimensionless maximum scour depth values relative to their location in the downstream straight reach of the 180-degree bend.

Regarding the location of maximum scour around the pipeline along the downstream straight reach of the 180-degree bend, the experiments in which the pipe was installed above the bed or level with the bed indicated that the maximum scour occurred at a longitudinal distance of 1.5 pipe diameters. In case when the pipe was buried within the bed, Maximum scour occurred at a longitudinal distance of 4 pipe diameters along the channel. This difference in the location of maximum scour was attributed to variations in the manner in which the flow impacted the pipeline and was subsequently redirected in different directions in the form of upward and downward flow components, as well as differences in flow separation patterns upon interaction with the pipeline structure. Direct interaction between the flow and the pipe in pipe positions of level with bed and above the initial bed level altered the flow velocity and direction, leading to corresponding changes in bed shear stresses around the pipeline; as a result, the maximum scour developed at a short distance from the pipe. In contrast, in the buried configuration, the reduced direct contact between the flow and the pipe caused the zones of maximum bed shear stress to shift farther from the pipe location, resulting in the occurrence of maximum scour at greater longitudinal and transverse distances compared to the other two configurations.

The comparison of the present study’s results with previous investigations shows that the mechanism of scour occurrence at a longitudinal position relative to the channel center is modified by the upstream bend of the pipe. The scour behavior observed in this study reveals significant differences in the longitudinal position of maximum scour relative to the pipe center compared with the results of Bijanvand et al. (2021) for a straight reach. In Bijanvand et al., as the pipe burial depth relative to the bed decreased from above-bed to pipe installation level with the bed and, finally, to buried at a depth of D/4, the longitudinal position of maximum scour relative to the pipe center decreased accordingly; in other words, the above-bed installation produced the largest longitudinal distance between the pipe center and the position of maximum scour [20]. By contrast, in the present study, where the pipe was installed downstream of a bend, the pattern changed: the buried configuration produced the greatest longitudinal distance of maximum scour relative to the pipe center, whereas for the pipe installation level with the bed and the above-bed installation, the position of maximum scour occurred at shorter longitudinal distances than in the buried case. This difference in the spatial behavior of the maximum scour position reflects the dominance of bend induced helical and secondary flows over the pipe induced vortex structure, which, in the buried pipe condition, exerts a different influence on the downstream sediment transport pattern.

Fig 4 presents the maximum sedimentation and its location along the downstream straight reach of the 180-degree bend for different pipe installation conditions and various rations of unsteady flow conditions.

**Fig 4:**
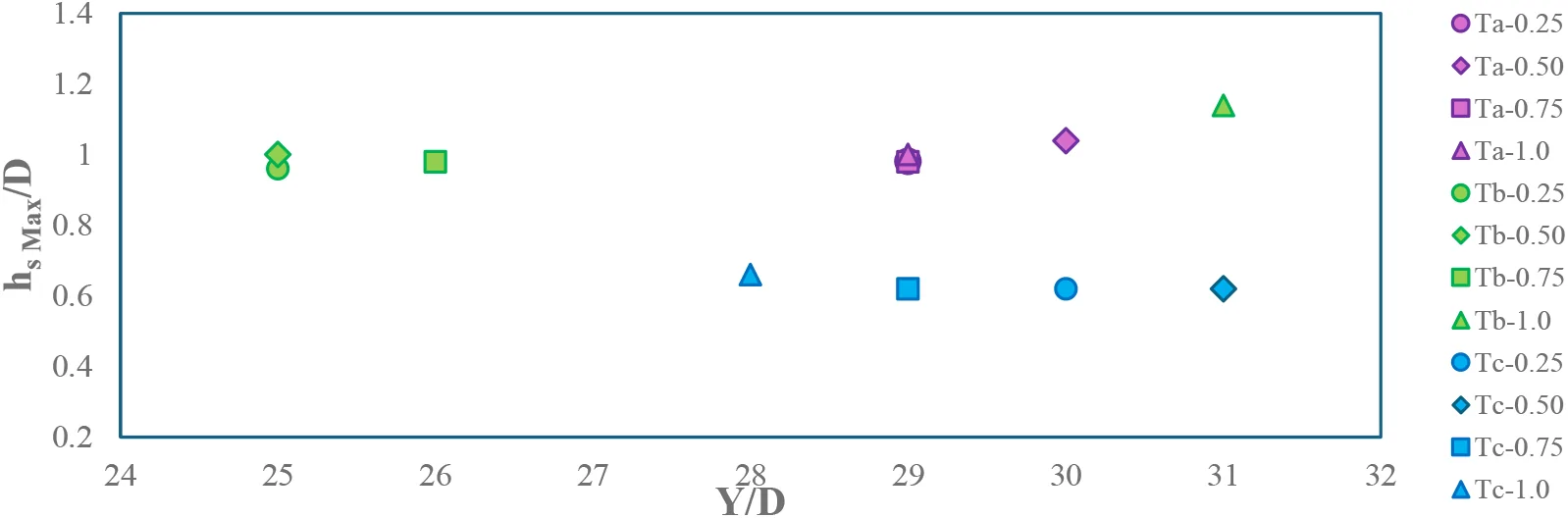
Dimensionless maximum sedimentation height values relative to their location in the downstream straight reach of the 180-degree bend.

Since the maximum sedimentation commonly occurred when the ratio of the peak discharge time to the flooding duration was equal to 1, in terms of the location along the downstream straight reach of the 180-degree bend for three pipe installation conditions – level with the bed, above the bed, and buried within the bed – the maximum sedimentation occurred at positions equal to 31, 29, and 28 pipe diameters, respectively, measured along the downstream straight reach. Therefore, it can be concluded that, as sedimentation increased due to changes in the pipe position relative to the initial bed level, the maximum sedimentation occurred farther away from the pipe location and toward downstream. Conversely, when sedimentation decreased, the maximum sedimentation occurred in a position closer to the pipe. This behavior is attributed to differences in sediment transport capacity and flow pattern caused by the pipe’s installation relative to the bed. When the pipe was installed level with the bed, the flow disturbance induced by the formation of a small falling jet from the top of the pipe—particularly at the early stage of the experiment—led to a localized increase in velocity and bed shear stress in the downstream region, resulting in an increase in the sediment transport capacity. Consequently, sediment particles detached from the bed remained suspended for a longer distance and their subsequent deposition took place farther downstream from the pipe location. With a reduction in the area of direct flow–pipe interaction, particularly in the buried condition, the sediment transport capacity of the flow decreased, causing sediment particles to settle at a shorter distance from the pipe location. As a result, the zone of maximum sedimentation shifted toward regions closer to the pipeline. These findings highlighted the decisive role of pipeline installation configuration in controlling the spatial distribution of sedimentation and the stability of the riverbed downstream of bends. position of maximum sedimentation relative to the pipe center is also influenced by the upstream bend. In Bijanvand et al. (2021), conducted on a straight reach, the longitudinal distance of maximum sedimentation from the pipe center continuously decreased as the pipe installation changed from above-bed to pipe installation level with the bed and then to buried at a depth of D/4 [20]. By contrast, in the present study, performed on the straight downstream reach of the bend, the pattern differed: the largest longitudinal distance of maximum sedimentation was observed for the pipe installation level with the bed, followed by the above-bed case, while the buried configuration exhibited the smallest distance. This contrasting behavior can be attributed to the effect of secondary flows and the interaction between the bend’s outflow and the cross-flow structure around the transverse pipe at the Bend Exit, which alters sediment transport and deposition patterns relative to a straight reach pipe installation.

### 3.2. Bed topography analysis

Fig 5 illustrates the bed topography of the downstream straight reach of the 180-degree bend in the presence of the pipeline.

**Fig 5:**
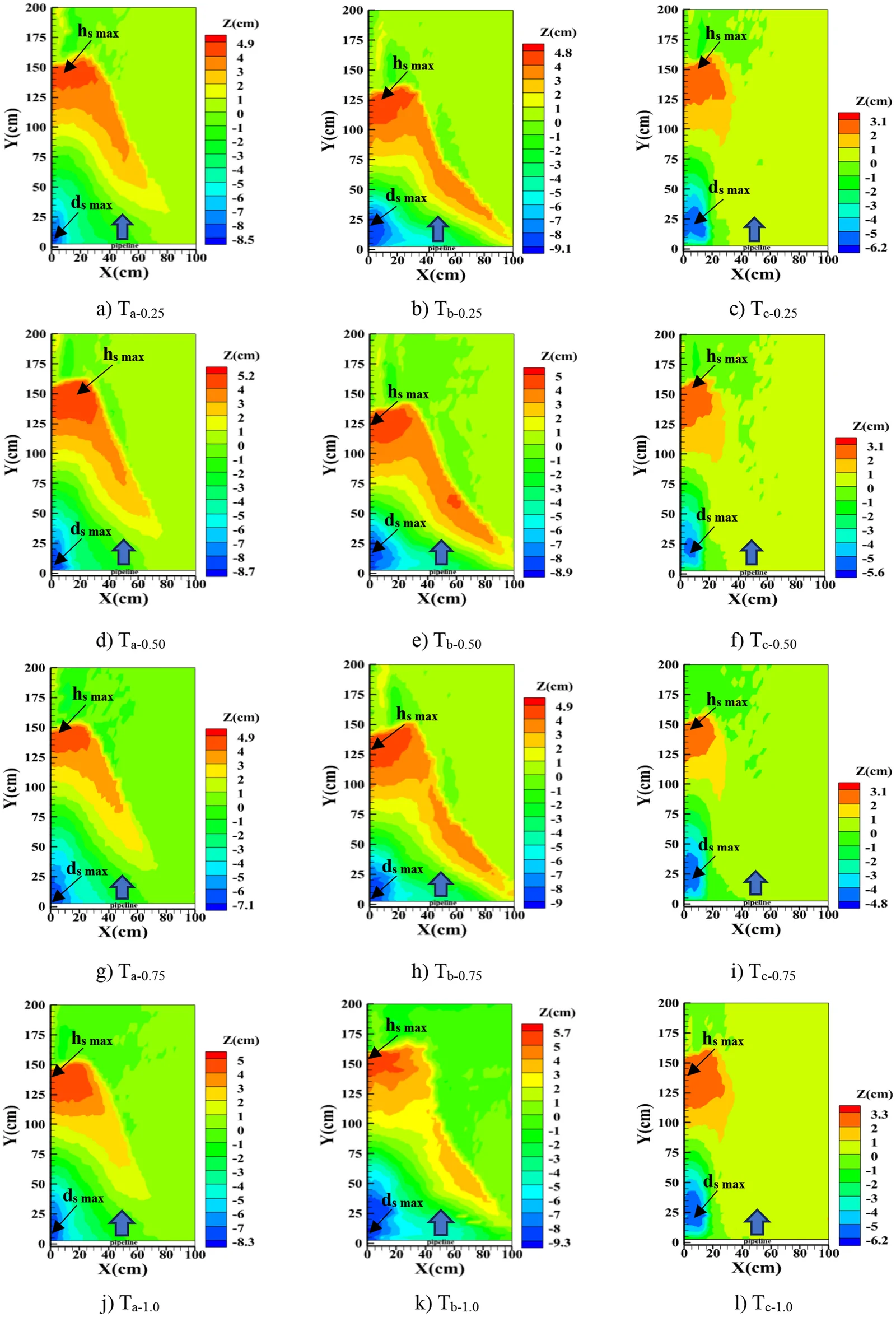
The bed topography of the downstream straight reach of the 180-degree bend in the different pipeline installation configurations relative to the bed level.

Analysis of the bed topography changes downstream of the 180° bend in the presence of the pipe shows that installing the pipe perpendicular to the flow direction leads to a distinct pattern of bed deformation. In all T_r_/T_d_ ratios and pipe installation configurations, the bed changes at the end of the experiment evolved such that, with progression towards the downstream of the pipe, the major part of the bed deformation was concentrated along the left bank of the channel. This phenomenon can be attributed to the nature of secondary flow in bends, where velocity vectors near the water surface are driven towards the outer bank and, upon impinging on this bank, intensify scour and sediment transport in that region. This behavior al pattern is in close agreement with the observations of Valizadeh et al. (2023) for semi-buried pipes in a mild 90° bend [23]. The consistency between the present findings and those reported by Valizadeh et al. for a mild bend indicates that, even when the relative bend curvature radius changes from mild to sharp, the primary mechanism of scour concentration along the outer bank for pipes perpendicular to the flow remains the dominant driver of bed morphology adjustment.

the most pronounced bed topography changes occurred when the pipe was installed level with the bed. In this configuration, the interaction between the secondary flow exiting the upstream bend and its direct impact on the pipe near the outer bank of the channel, through modification of the bed shear stress around the pipe and changes in flow velocity, caused the extent of the scour and sedimentation zones concentrated along the left bank to become larger than in the other pipe installation configurations. In contrast, for all pipe installation configurations and hydrographs considered, the smallest bed topography changes in terms of both scour and sedimentation were observed when the pipe was installed in the buried condition. In this pipe installation configuration, because the pipe is not directly exposed to the flow during the initial stages of the experiment, its influence on the flow field is weaker than in the other configurations. However, due to the bend upstream of the pipe and the stronger concentration of incoming helical flows towards the straight reach along the left bank compared with the right bank, sediments in this region gradually move over the pipe during the experiment. The removal of the sediment cover from the pipe surface leads to direct contact between the flow and the pipe, thereby modifying flow direction and velocity and ultimately changing the bed topography by forming a local scour hole around the pipe and advancing the eroded sediments towards the downstream of the pipe during the remaining experiment time in the buried condition. In the buried configuration, flow alterations due to pipe–flow contact are weaker than in the configurations where the pipe is exposed to the flow throughout the experiment, so the flow does not have sufficient capacity to transport sediments to more distant areas. Consequently, bed changes in the buried condition are smaller than in the other pipe installation configurations because of the reduced obstruction effect of the pipe.

For the pipe installed above the bed, the extent of bed topography variations lay between the other two configurations, such that the scour and sedimentation zones were more pronounced than those of the buried condition but less extensive than those of the level-with-bed condition. The direct presence of the pipe as an obstacle to the flow and the contact between the flow and the pipe in this configuration also produce greater bed topography changes than in the buried condition, due to the resulting modifications in the flow structure. However, because part of the flow passes beneath the pipe, the shear generated around the pipe is lower than in the pipe installation level with the bed condition, and consequently both the intensity and spatial extent of the bed topography changes are smaller than in the pipe installation level with the bed configuration.

According to the explanations provided, The most significant downstream bed topography changes occurred for the level-with-bed installation in test T_b-1.0_, with a maximum scour depth of 9.3 cm (equivalent to 1.8 pipe diameter) and a maximum sedimentation of 5.7 cm (equivalent to 1.1 pipe diameter). Furthermore, the least pronounced downstream bed topography changes were associated with the buried pipe configuration in test T_c-0.75_, where the maximum scour depth was 4.8 cm (equivalent to 0.9 pipe diameter) and the maximum sedimentation was 3.1 cm (equivalent to 0.6 pipe diameter). Thus, it may be inferred that changing the pipe installation condition from level with the bed, which produced the greatest bed topography changes, to the buried condition, which produced the smallest bed topography changes, led, on average, to a 37.1% reduction in the scour hole around the pipe and a 38.0% reduction in sedimentation volume downstream of the pipe.

Moreover, regarding the effect of the ratio of peak discharge time to flooding duration on scour around the pipeline located downstream of the 180 degree bend, Given that the peak discharge was kept the same for all four hydrographs, the observed differences in scour depth cannot be attributed to the magnitude of the peak discharge. The results also show that the hydrograph with a peak-time-to-duration ratio of 1.0 produced the greatest changes in bed topography, whereas the ratio of 0.75 resulted in the least bed-topography change. This indicates that the timing of peak discharge played an important role in the development of scour and bed deposition. Moreover, because secondary flow in the upstream curved reach of the pipe was present in all examined cases, this flow pattern was observed in every configuration, and its effect on downstream bed-topography change was evident under the same geometric bend conditions. Likewise, the cumulative shear stresses associated with flow impingement on the pipe were present to the same extent in all pipe-installation conditions. Therefore, only the effect of hydrograph peak timing, under the three pipe-positioning conditions relative to the initial bed, was considered influential and was examined here.

since the greatest bed topography changes occurred at a T_r_/T_d_ ratio of 1.0 and the smallest changes occurred at a T_r_/T_d_ ratio of 0.75, it can be concluded that decreasing the ratio of peak discharge time to flooding duration from 1.0 to 0.75 resulted in an average 13.3% reduction in scour and a 7.3% reduction in sedimentation. This indicates that the influence of the hydrograph shape on pipeline scour downstream of the 180 degree bend was smaller than the influence of the pipeline burial depth.

### 3.3. Cross-sectional bed profiles

Figs 6–8 present the transverse bed profiles of the straight reach downstream of the 180 degree bend in the presence of the pipeline for the three installation conditions and four hydrograph shapes of the rising limb and the peak discharge time to duration at different sections, extracted at distances of 2, 5, and 10 times the pipe diameter along the channel. Based on Figs 6 and 7, which depict bend’s downstream straight reach bed cross sections corresponding to the above bed and level with bed installation conditions, it was observed that for all four T_r_/T_d_ ratios and in all sections for these two pipe conditions, scour occurred near the left bank. This is due to the impact of the secondary flow exiting the 180 degree bend on the left bank and its continuation into the downstream straight reach and the increase in the shear stress affecting the bed due to down flows on surface and toward the left bank, ultimately intensifying scour beneath the pipeline in that region. Gradually distancing away from the pipeline installation point toward downstream, from the 2 pier diameter section to the 10 pier diameter section, sedimentation increase was observed in the central region of the channel bed due to the reduction in the velocity of the bend’s outflow in the downstream straight reach, creating the conditions for the settling of the sediment transported from the bend and exiting from the hole formed around the pipeline.

**Fig 6:**
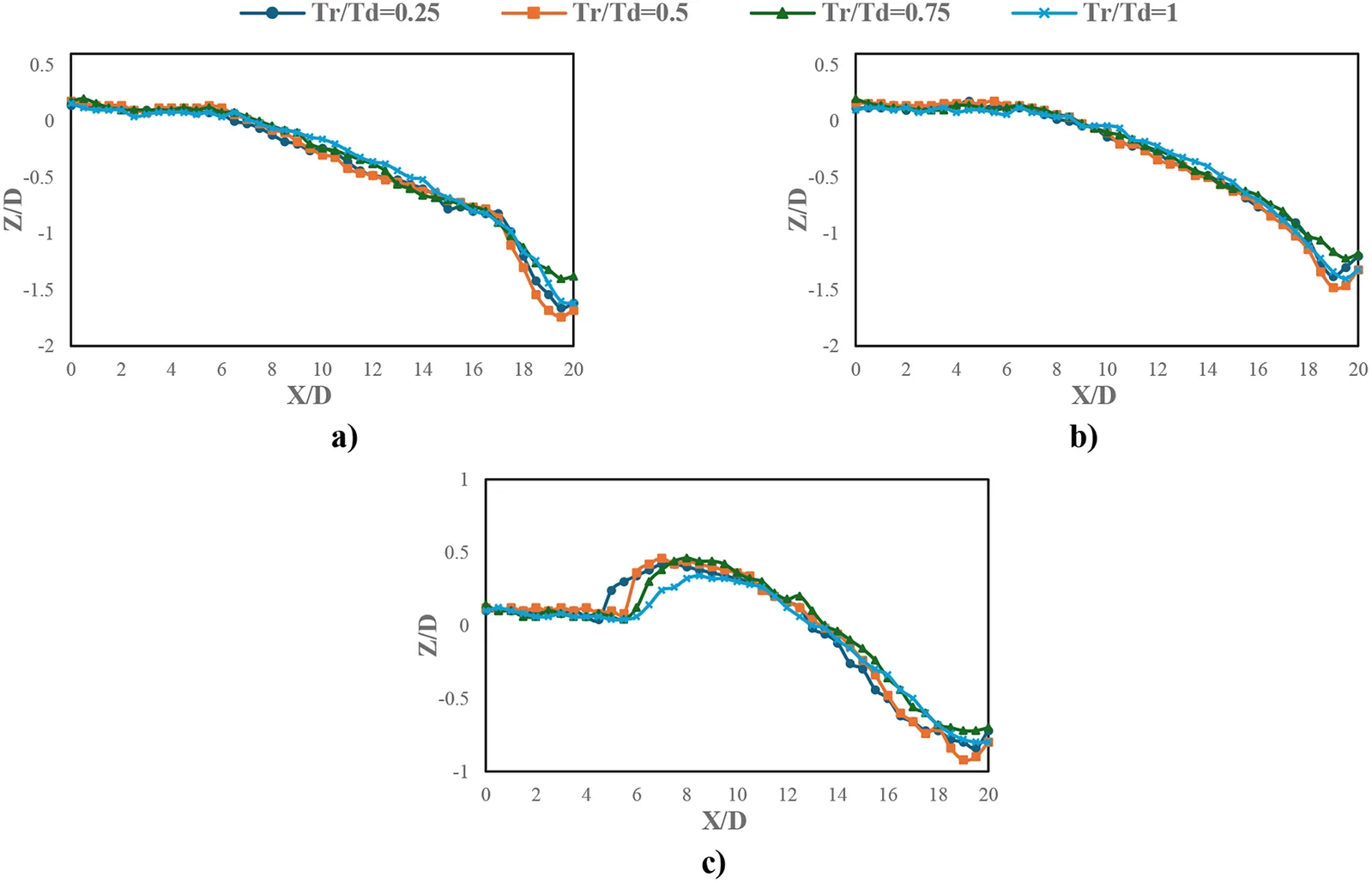
Instance of bed profiles in different cross sections for the above-bed pipe condition at pipe distances downstream corresponding to (a) 2, (b) 5, and (c) 10 times the pipe diameter.

**Fig 7:**
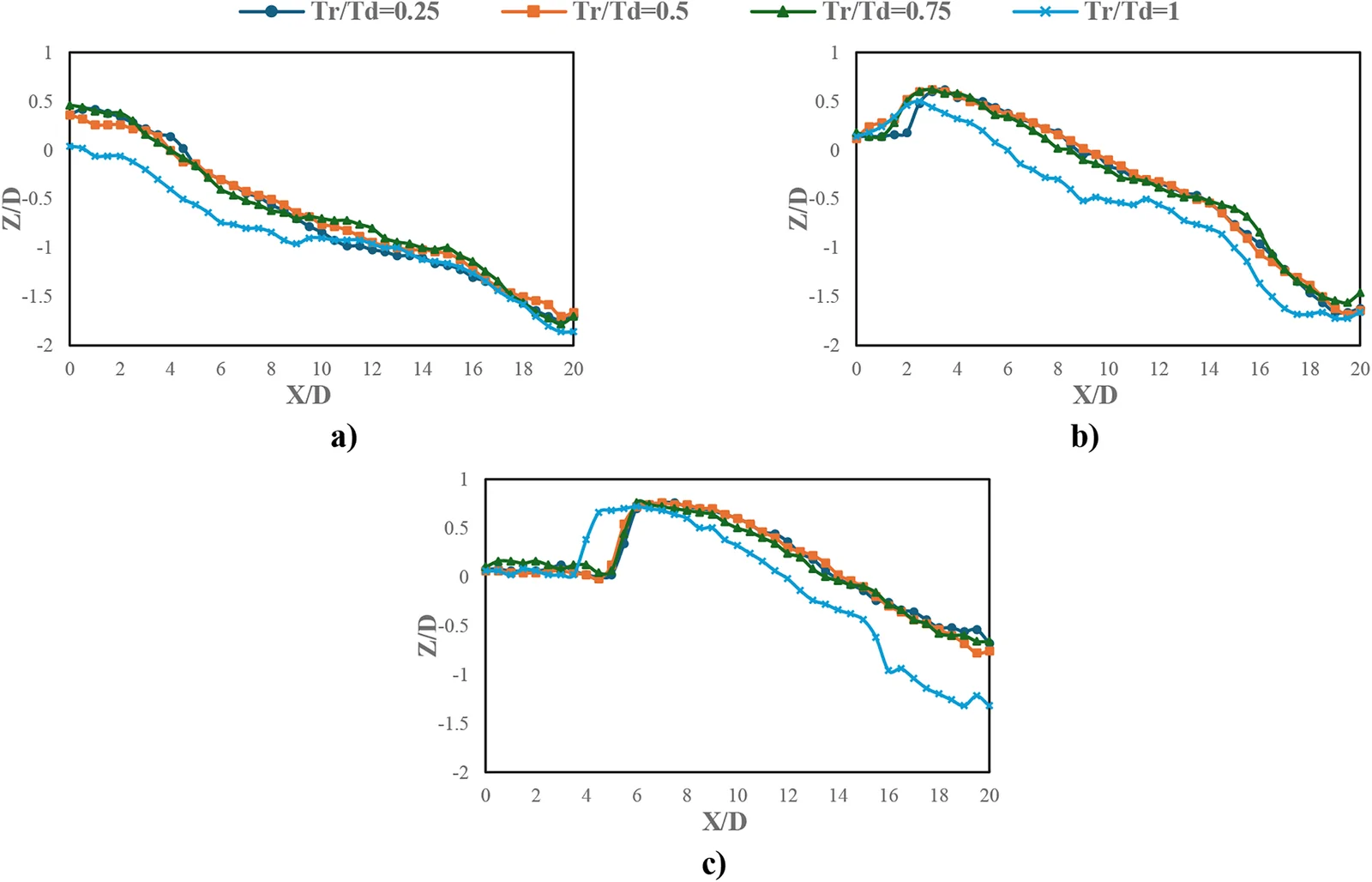
Instance of bed profiles in different cross sections for the level-with-bed pipe condition at pipe distances downstream corresponding to (a) 2, (b) 5, and (c) 10 times the pipe diameter.

**Fig 8:**
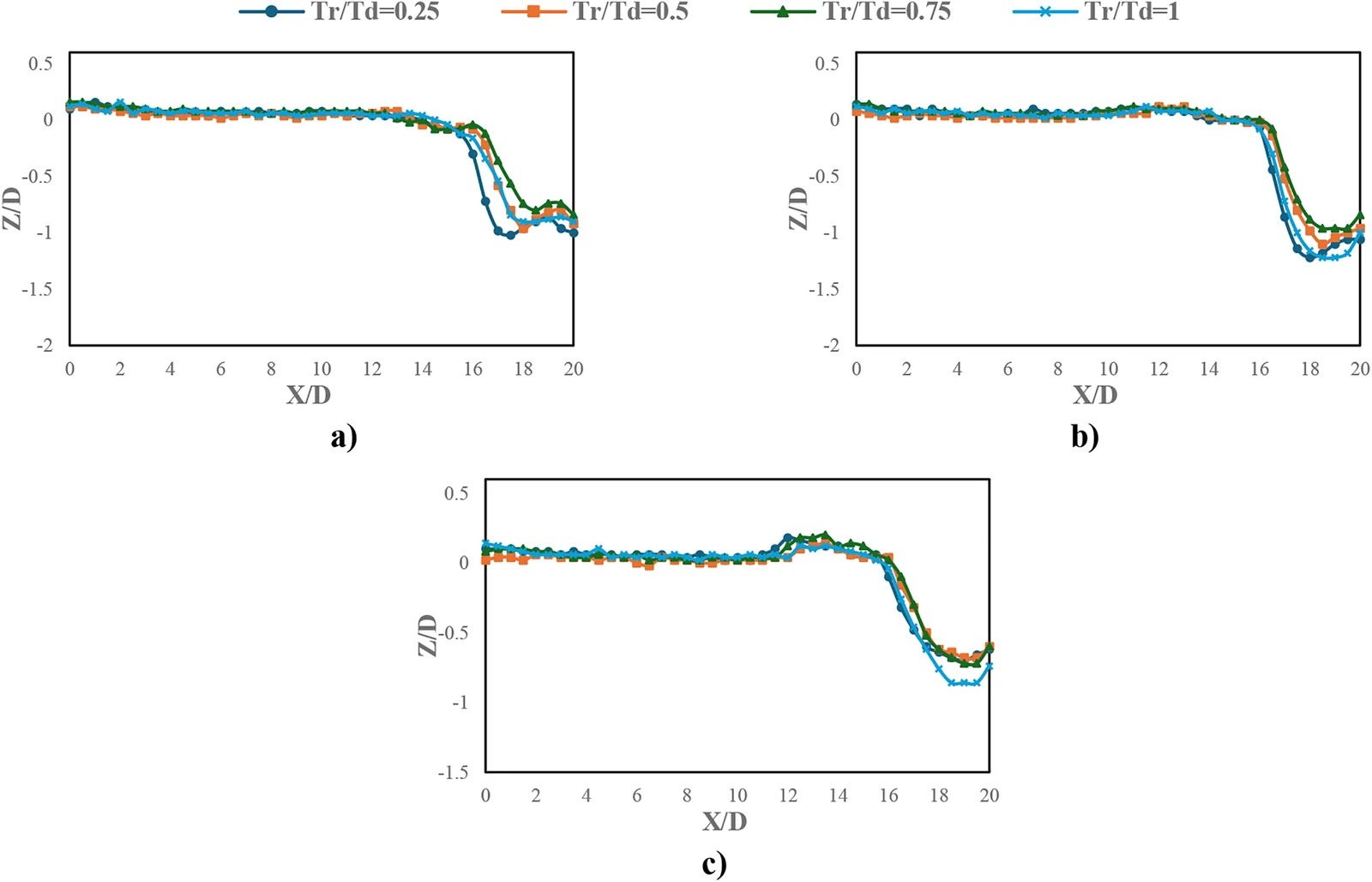
Instance of bed profiles in different cross sections for the buried pipe condition at pipe distances downstream corresponding to (a) 2, (b) 5, and (c) 10 times the pipe diameter.

Additionally, gradually distancing away from the pipeline installation site along the channel, the depth of the scour hole adjacent to the left bank gradually decreased. A comparison of Figs 6a and 7a shows that the size of the downstream scour hole near the left bank is larger for the level with bed installation than for the above bed installation. This difference is attributed to the stronger flow turbulence in the pipe installation level with the bed condition compared with the above bed installation.

Fig 8 illustrates the transverse bed profiles of the straight reach downstream of the bend for the buried pipe condition. The examinations indicated that, similar to the other installation configurations, scour in this case also occurred in the vicinity of the left bank due to the bend’s outflow and the concentration of flow near this side of the channel. Moreover, for all four Tr/Td ratios and across all cross sections, bed changes under this pipe installation condition from the inner bank to approximately the channel mid width were relatively constant and stable, and were predominantly characterized by sedimentation. Further away from the pipe location toward the downstream along the channel, as the flow fully entered the straight downstream reach and the influence of the pipe on flow turbulence diminished at larger distances, the magnitude of scour along the left bank progressively decreased. A comparison of the cross sections corresponding to different pipe installation conditions relative to the initial bed level indicated that, in the buried configuration, sedimentation and scour attained their minimum magnitudes due to the protection of the pipe from direct flow impingement during the experimental period and the negligible influence of the buried pipe on the overall flow structure. In contrast, the pipe installation level with the bed exhibited the maximum sedimentation and the greatest scour depth. In addition, in all experiments, the effect of the ratio of peak discharge time to flooding duration on variations in bed scour and sedimentation in the downstream straight reach of the bend was relatively minor when compared with the influence of the pipe installation condition.

### 3.4. Longitudinal bed profiles

Figs 9–11 present longitudinal bed profiles of the downstream straight reach of the 180 degree bend in the presence of the pipeline, for three installation configurations relative to the initial bed level at sections located at 5%, 50%, and 95% of the channel width.

**Fig 9:**
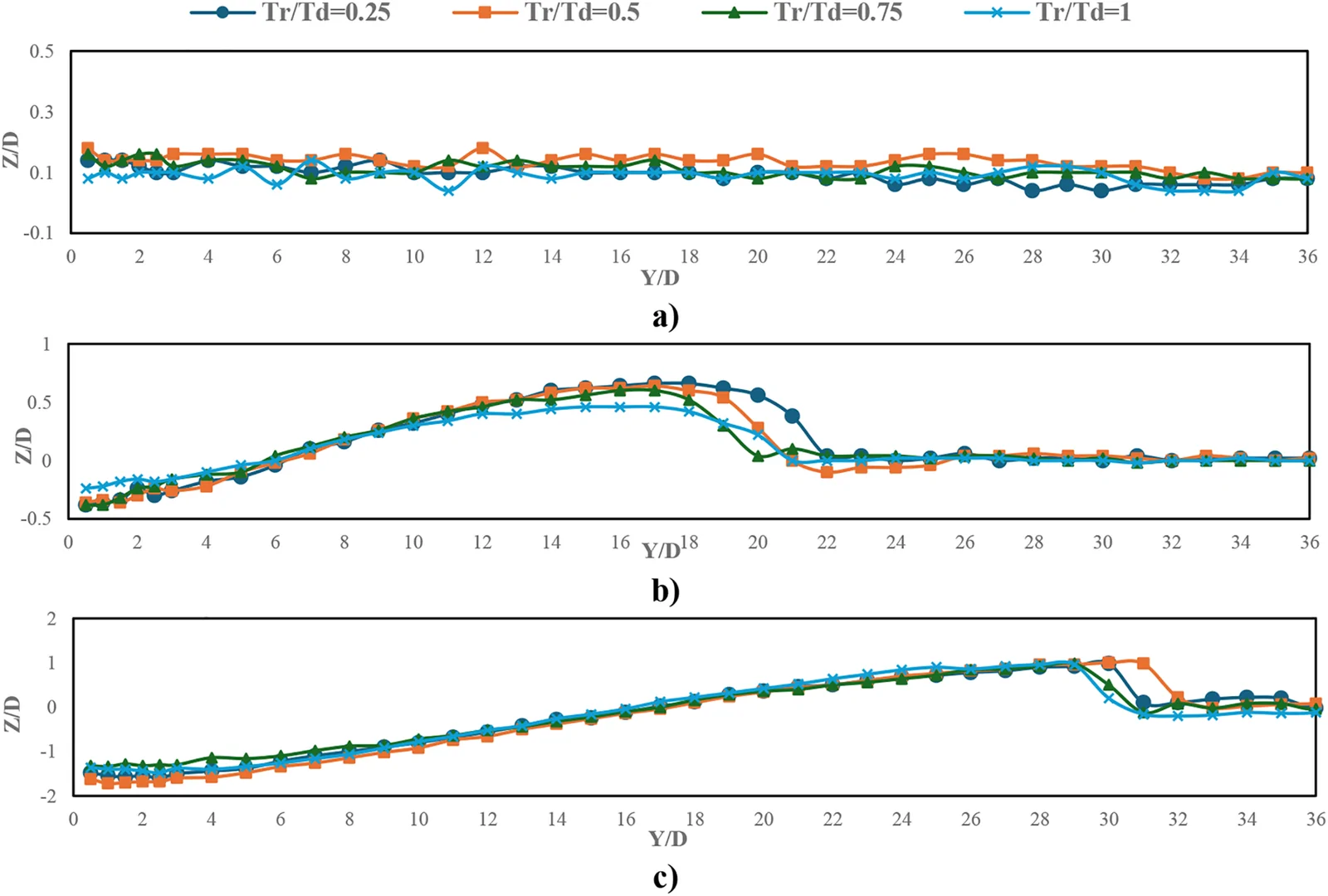
Instances of longitudinal bed profiles for the above-bed pipe installation at distances of (a) 5%, (b) 50%, and (c) 95% of the channel width from the right bank.

**Fig 10:**
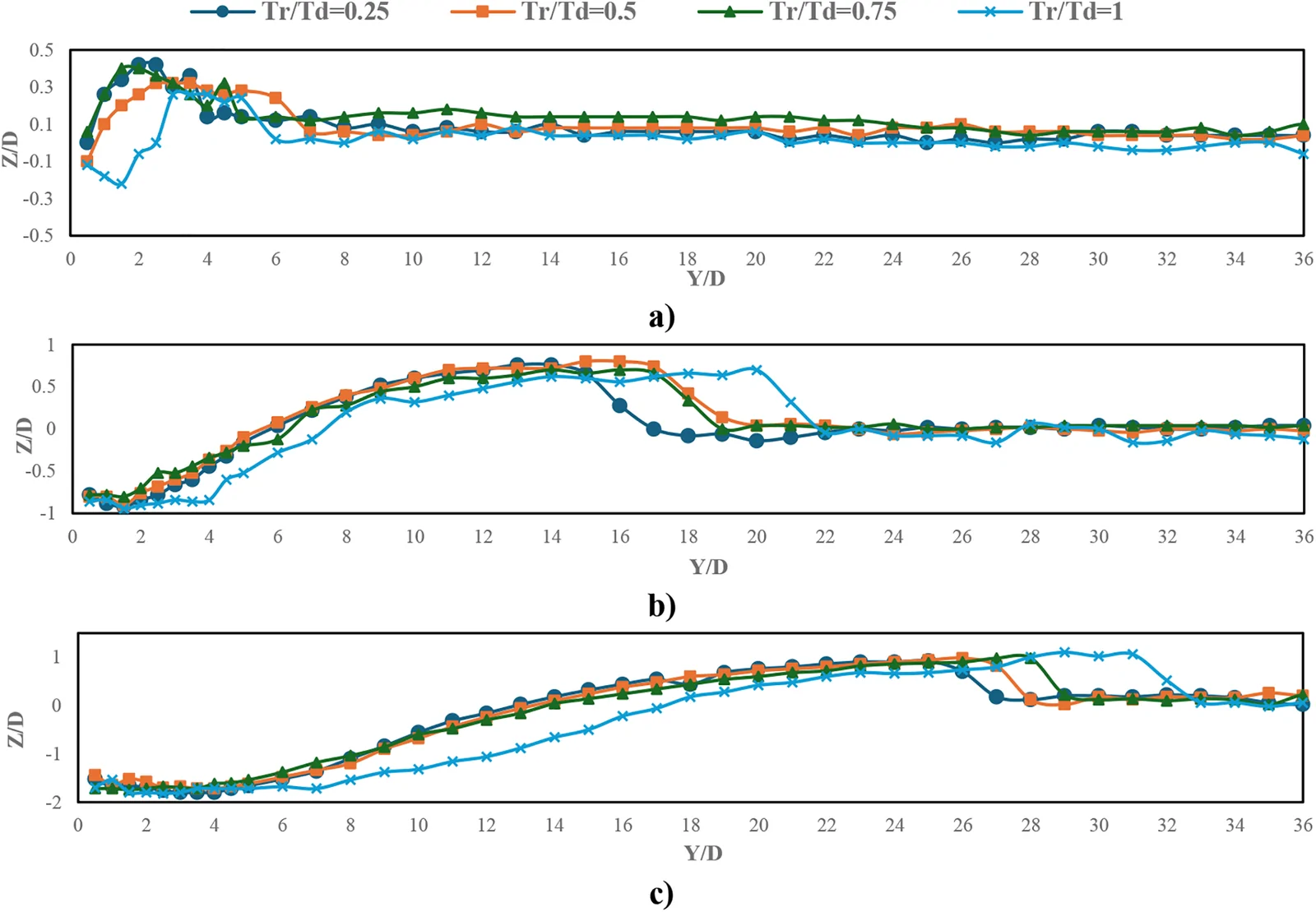
Instances of longitudinal bed profiles for the level-with-bed pipe installation at distances of (a) 5%, (b) 50%, and (c) 95% of the channel width from the right bank.

**Fig 11:**
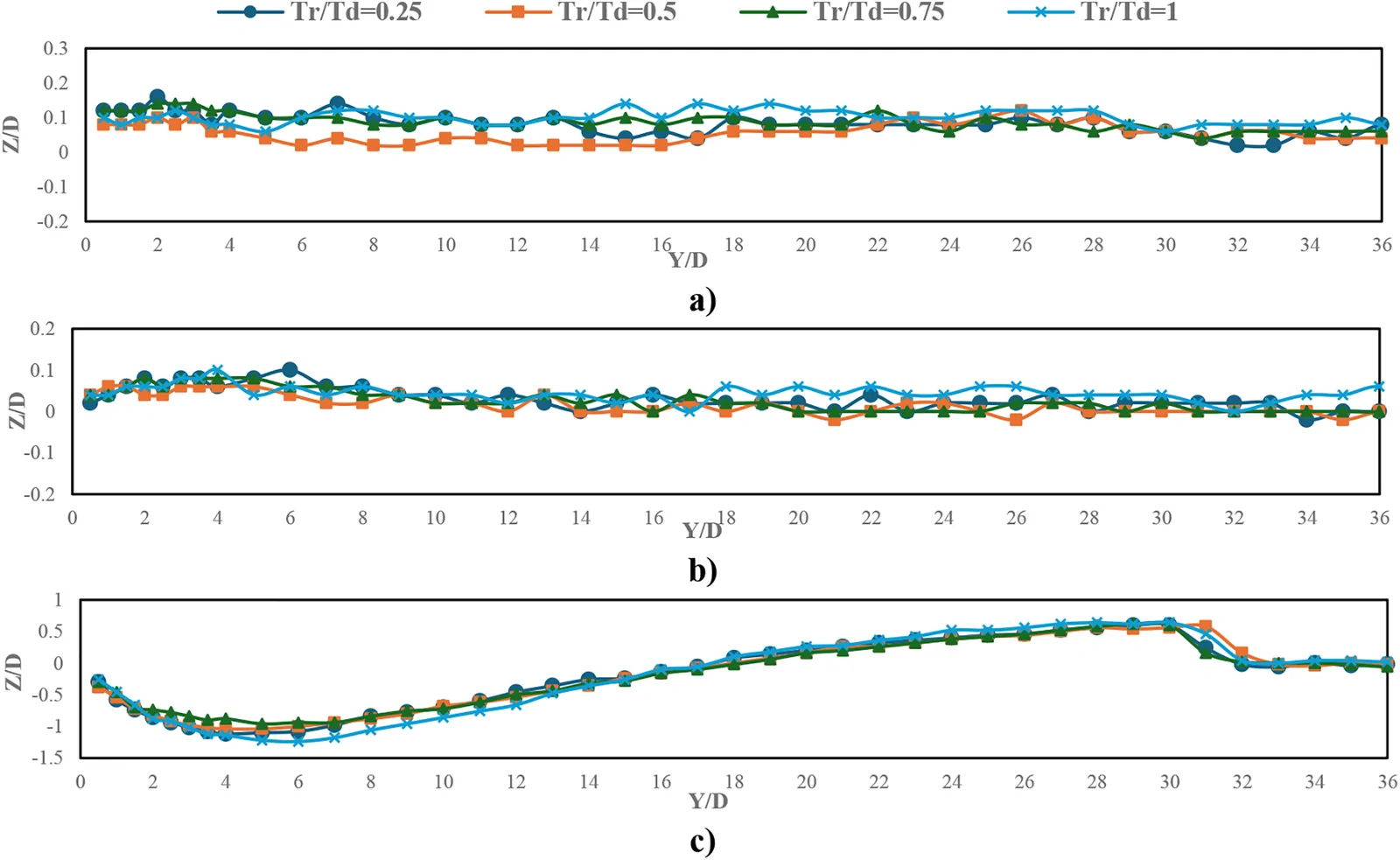
Instances of longitudinal bed profiles for buried pipe installation at distances of (a) 5%, (b) 50%, and (c) 95% of the channel width from the right bank.

Based on Fig 9, which depicts the above bed pipe installation, the longitudinal profile at the 5% width section from the right bank of the channel shows that a relative reduction in flow velocity in this region, due to the persistence of bend induced helical flow towards the left bank along part of the downstream bed, led to the dominance of sedimentation processes and the formation of a stable sedimentation pattern along the reach near the right bank. Moreover, at the 50% width section in the channel center, the presence of the pipe and its influence on flow velocity increased bed shear stresses in the vicinity of the pipe, resulting in local scouring; further downstream, with gradual energy dissipation, sediment transport capacity decreased and sedimentation became dominant at intermediate distances, while the weakening of bend induced helical flow and the more uniform distribution of velocity caused bed changes to occur more smoothly. At the 95% of the channel width from the right bank, the persistence of secondary flow energy exiting the bend and its interaction with the pipe led to scour extending over a longer downstream distance from the pipe location; with the gradual weakening of the flow farther downstream, conditions became favorable for sedimentation.

Fig 10 compares the longitudinal bed profiles for the level with bed installation condition. At the longitudinal section at 5% of the channel width from the right bank, a relative reduction in velocity and bed shear stress, together with the effect of the level with bed pipe acting as an obstacle to the flow, resulted in sediment accumulation at the beginning of the reach, and at greater distances from the pipe location along the channel, sedimentation continued in a more uniform manner.

In addition, at the 50% width section in mid channel, the concentration of shear stresses in the vicinity of the pipe led to local scouring, which, as flow energy gradually dissipated downstream, transitioned into a sedimentation region. At the 95% of the channel width from the right bank, similar to the above bed installation, the persistence the flow exiting the bend caused scour to extend over a longer distance from the pipe location, and with the gradual dissipation of flow energy as the distance from both the bend and the pipe increased, sedimentation became the dominant process along the downstream reach.

Fig 11 compares the longitudinal sections for the buried pipe condition. At the 5% and 50% of the channel width from the right bank, the reduced direct interaction between the flow and the pipe, together with the dominance of sedimentary behavior in the downstream straight reach near the right bank and mid-channel, resulted in shear stresses that were insufficient to induce erosion. Consequently, uniform and stable sedimentation was observed in these regions. In contrast, at the 95% of the channel width from the right bank, the residual energy of the secondary flow exiting the bend produced limited scour in the vicinity of the pipe. As the flow weakened downstream, this scour zone transitioned into a sedimentation region at intermediate distances along the channel, and eventually the bed reached a stable condition.

A comparison of the longitudinal bed profiles for the three pipe installation configurations demonstrated that the installation condition played a decisive role in controlling both the intensity and spatial distribution of scour and sedimentation in the downstream straight reach of the bend. In the level-with-bed condition, the greatest disturbance to the flow structure, caused by the formation of a falling jet over the pipe, produced more intense local scour near the pipe installation point, with a wider downstream extent of influence. For the above-bed installation, bed erosion was reduced compared with the level condition, and a more balanced distribution of scour and sedimentation was observed. Conversely, in the buried configuration, the flow–pipe interaction was minimized, and bed changes occurred predominantly in the form of uniform and stable sedimentation, with a marked reduction in scour. These findings indicated that pipe burial could be considered the most effective strategy for controlling bed erosion and enhancing hydraulic stability in the downstream straight reach of channel bends.

### 3.5. Statistical analysis

Fig 12 presents the effect of hydrograph shape on the maximum scour downstream of the pipe installed in the downstream straight reach of the bend. For the hydrograph with a peak time equal to the base time, the maximum scour depth exceeds the mean value across the three pipe installation configurations relative to the initial bed level. In this unsteady‑flow hydrograph, the longer duration of high discharges during the approach to peak, together with the interaction between the pipe‑induced vortices and the bend‑induced helical flow, intensifies bed shear stress and increases the maximum scour depth to values higher than the mean for each installation condition. In contrast, for the hydrograph with a peak time equal to 75% of the base time—which represents a flood with a delayed peak discharge relative to the normal case—the maximum scour depth in all three pipe installation configurations is lower than the corresponding mean value, due to sediment infilling within the scour hole. In other words, for this ratio of rising to falling limb, before the downstream vortices around the pipe can enlarge the scour hole to its maximum depth, the flow enters the falling limb, thereby limiting the scour development and reducing the maximum depth relative to the mean value. When the peak time is equal to 50% of the base time, the simultaneous effects of sediment exiting the downstream scour hole and sediment collapsing into the hole from upstream cause the maximum scour depth to deviate from the mean value in a configuration‑dependent manner. Finally, when the peak time is equal to 25% of the base time, because the peak occurs early in the experiment, the maximum scour depth in all three pipe installation configurations relative to the initial bed level is greater than the mean value.

**Fig 12:**
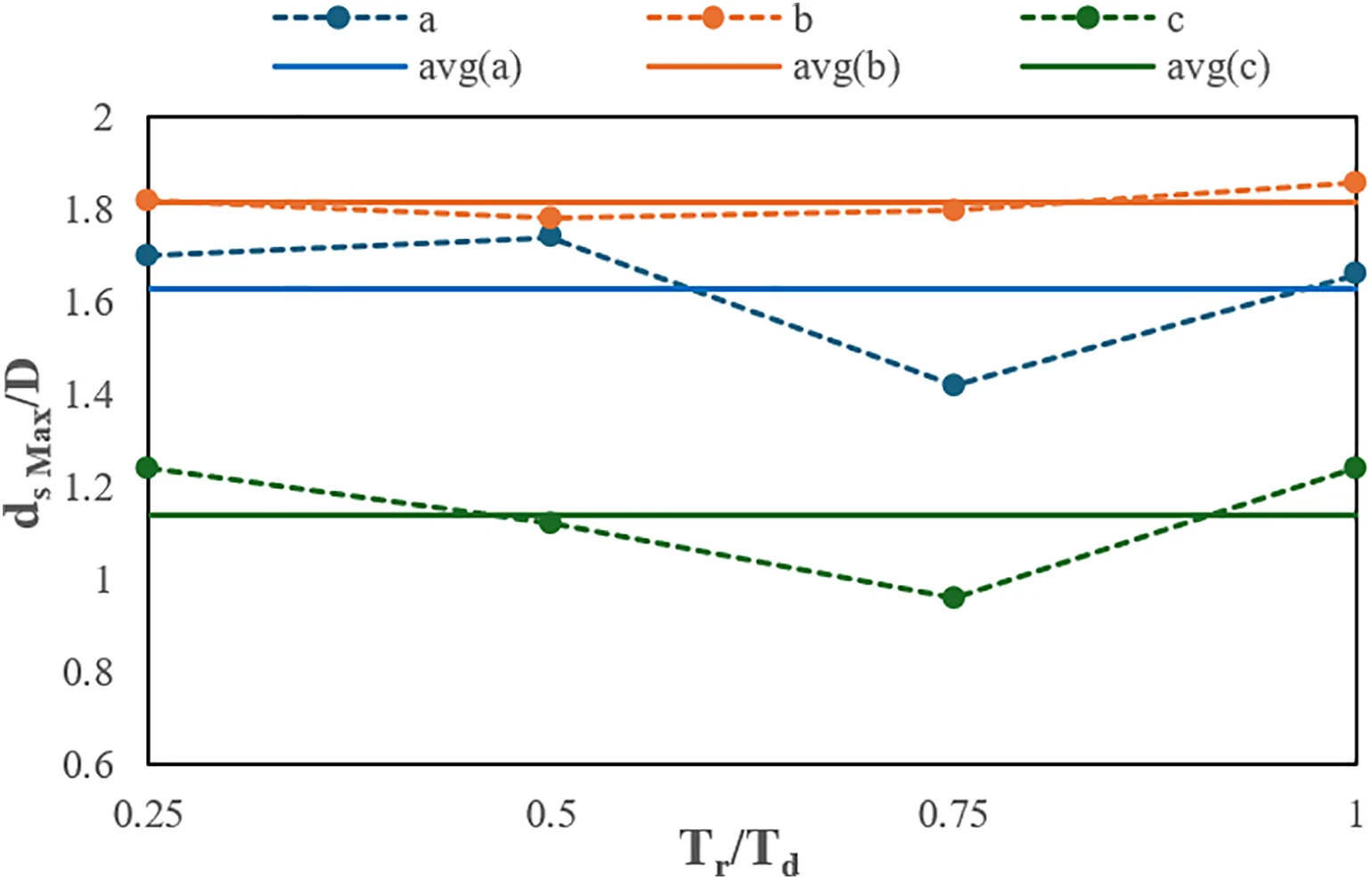
Influence of the hydrograph rising‑to‑falling limb ratio on maximum scour for different pipe installation conditions.

In the level‑with‑bed pipe installation condition, which represents the most critical installation configuration among the tested cases, the presence of the pipe as a direct obstacle to the flow results in the highest maximum scour in all four hydrographs compared with the other installation conditions. However, in this pipe installation condition, the direct interaction between the bend’s outflow and the pipe structure, through its effect on flow velocity, tends to bring the downstream scour around the pipe to an approximately equilibrium state. Therefore, variations in the hydrograph peak time have a smaller influence on the maximum scour in this installation condition than in the other configurations; as the hydrograph transitions from the rising to the falling limb, no pronounced or significant change in the maximum scour magnitude is observed.

Fig 13 presents the effect of hydrograph shape on the maximum sedimentation downstream of the pipe installed in the downstream straight reach of the bend. The results show that, for all three pipe installation configurations relative to the initial bed level, the hydrograph with a peak time equal to the base time produces a maximum sedimentation greater than the mean value, whereas for hydrographs with peak‑time ratios of 0.25 and 0.75, the maximum sedimentation is lower than the corresponding mean value. In hydrographs whose falling limb has a gradual slope, the flow has sufficient time to continuously redistribute sediments along the reach; in other words, during the falling limb, the flow transports sediments from accumulation zones toward the downstream and prevents excessive deposition at a single location. By contrast, in the hydrograph with a peak time equal to the base time, the absence of a developed falling limb and the abrupt cessation of flow immediately after reaching the peak discharge suppress the continued sediment redistribution process. Consequently, all particles transported during the peak‑discharge period are deposited in place without the opportunity for further movement, leading to maximum sedimentation values higher than those of the other hydrographs.

**Fig 13:**
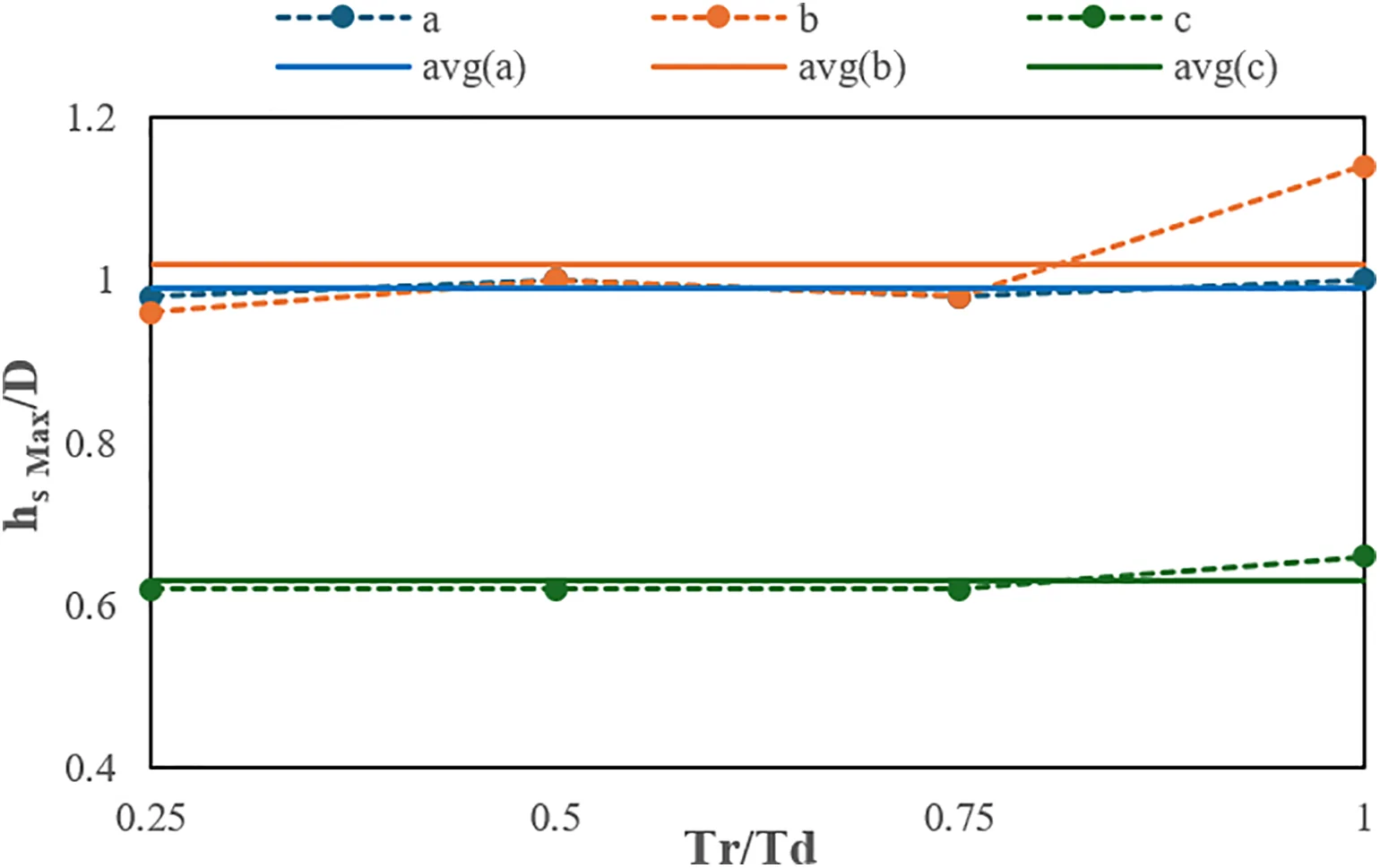
Influence of the hydrograph rising‑to‑falling limb ratio on maximum sedimentation for different pipe installation conditions.

In the analysis of the effect of hydrograph shape on maximum sedimentation, it was found that, unlike the scour response, changes in hydrograph shape under the level‑with‑bed pipe installation condition exert a pronounced influence on the maximum sedimentation. This is because, in contrast to scour, the sedimentation process is controlled by how the discharge decays over time, which determines whether sufficient time is available for particle settling. In the level‑with‑bed condition, where the pipe is located precisely at the interface between the flow and the bed, even small hydrograph‑induced variations in flow velocity lead to substantial fluctuations in the sedimentation magnitude. By contrast, the sensitivity of the above‑bed and buried installation conditions to changes in hydrograph shape is lower, and the impact on sedimentation is more limited. In the above‑bed installation, the pipe is offset from the bed surface, and in the buried installation, the flow interacts only indirectly with the pipe; in both cases, the turbulence generated by the pipe has a local character and its influence on the overall sedimentation process is reduced, causing the temporal changes in the hydrograph to have a markedly weaker effect on maximum sedimentation.

Fig 14 presents the dimensionless maximum scour versus the dimensionless maximum sedimentation for the different pipe installation conditions in the downstream straight reach of the 180° bend. The results indicate that the pipe installation condition relative to the bed level is the primary factor controlling the dominant direction of the process, although under certain specific hydrograph shapes, flow behavior can be substantially modified.

**Fig 14:**
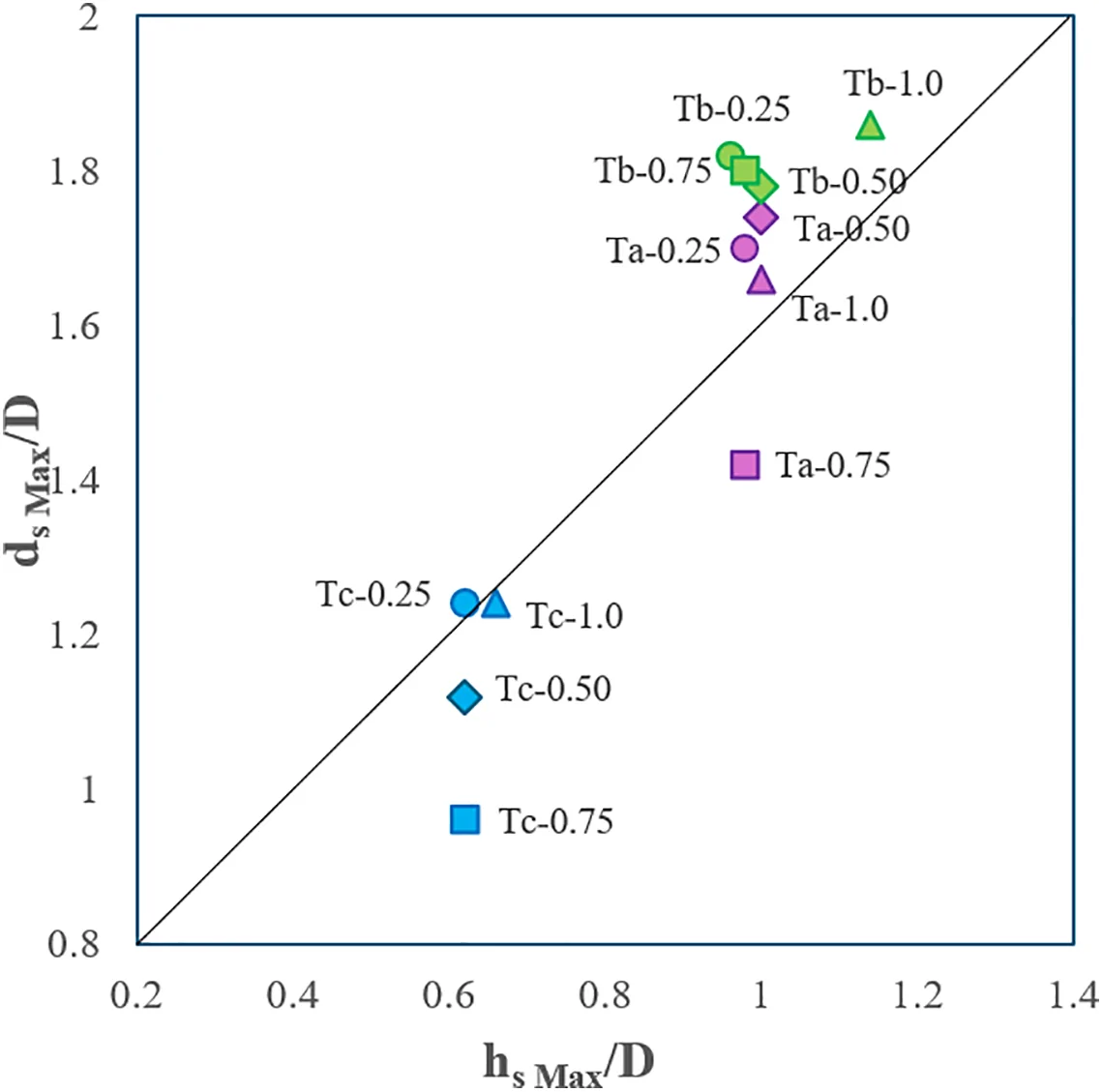
Dimensionless maximum scour versus dimensionless maximum sedimentation for different pipe installation conditions.

For the above‑bed pipe installations (Ta), most points lie above the bisector line, indicating that the maximum scour process dominates over maximum sedimentation. However, an atypical and exceptional behavior is observed for the Ta‑0.75 hydrograph: in this case, the corresponding point moves below the bisector line, showing that, unlike the other cases, maximum sedimentation dominates over maximum scour. For the level‑with‑bed installations (Tb), for all four hydrographs considered, the points lie above the bisector line, and the placement of the pipe directly in the flow path, together with the stronger turbulence of the bend’s outflow, leads to a dominant maximum scour process relative to maximum sedimentation. In the buried installations (Tc), in contrast to the other two conditions, all points lie below the bisector line, reflecting the dominance of maximum sedimentation over maximum scour. In this installation condition, the pipe is located beneath the bed surface and protected from direct flow impact, which promotes the formation of low‑velocity zones around the pipe and results in extensive sediment deposition.

To derive a mathematical relationship governing the experimental data, a power‑law equation was fitted to the laboratory results. Owing to the non‑linear form of the equation, linear regression based on the least‑squares method was applied to determine the model coefficients. The resulting empirical relationship provides a tool for predicting the dimensionless scour depth under different pipe installation conditions and hydrograph shapes. Table 2 presents the coefficients of the equation.

**Table 2:** Coefficients of Equation 1.

Coefficien	Configuration of pipe		
**a**	**b**	**c**
**α**	1.56	1.82	1.09
**β**	−0.06	0.01	−0.06


$$\begin{array}{c} \frac{{\text{d}}_{\text{s Max}}}{\text{D}}=\alpha {(\frac{{\text{T}}_{\text{r}}}{{\text{T}}_{\text{D}}})}^{\beta } \end{array}$$ (1)

After obtaining the empirical coefficients for each pipe installation condition, the proposed equation was used to compute scour depths for all experimental cases. To assess the predictive capability of the model, the deviation between the calculated and measured scour depths was quantified using the relative error percentage and illustrated in Fig 15. The scatter of the data points around the bisector line, together with the relative error values computed for each point, demonstrates the performance of the proposed predictive model.

**Fig 15:**
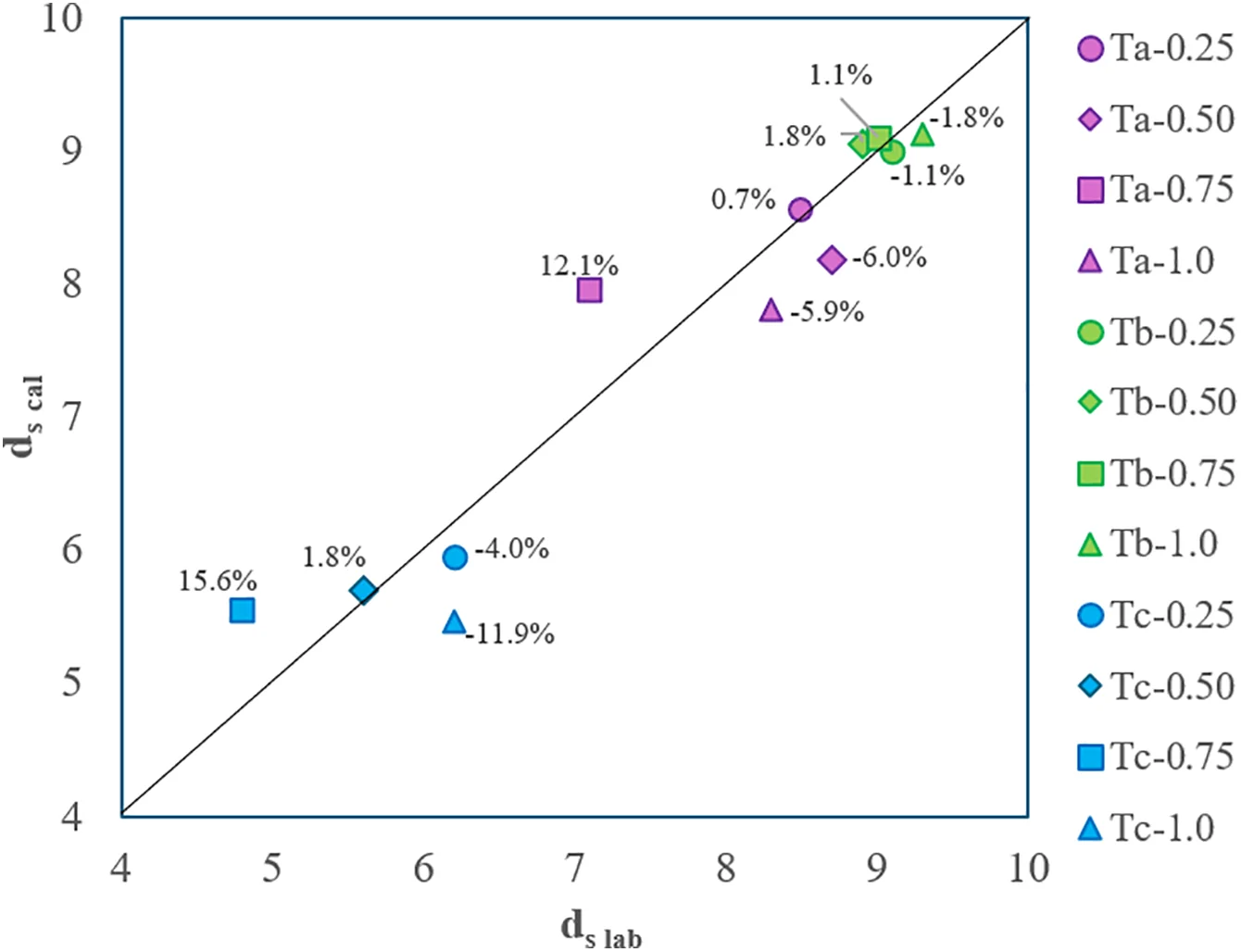
Ratio of experimental to computed maximum scour for different pipe installation conditions.

## 4. Conclusions

The objective of the present study was to investigate the effect the outflow of a 180-degree bend induced on local scouring around pipelines crossing the river width and installed at the beginning of the downstream straight reach. To this end, the pipeline was installed under different configurations relative to the initial bed level and subjected to various unsteady-flow hydrographs downstream of the curved reach. The resulting data were subsequently analyzed. The overall results of this study indicated that installing the pipeline level with the bed material downstream of the 180-degree bend produced the greatest alterations in bed topography and scour patterns in the bend’s downstream straight reach. Changing the pipe installation condition from level with the bed to a buried configuration led, on average, to a 37.1% reduction in scouring and a 38.0% reduction in sedimentation. In addition, an examination of the effects of the rising limb duration, the peak time, and the falling limb of the unsteady-flow hydrograph further revealed that the largest bed topography changes occurred at a Tr/Td ratio of 1.0, whereas the smallest changes occurred at Tr/Td of 0.75. Reducing the ratio of peak discharge time to flooding duration from 1.0 to 0.75 resulted, on average, in a 13.3% decrease in maximum scour and a 7.3% decrease in maximum sedimentation. Accordingly, it can be concluded that the influence of unsteady flow on reducing maximum scour downstream of the 180-degree bend was less significant than the effect of the pipeline installation condition relative to the initial bed level. The results also demonstrated that the pipeline installation configuration played a decisive role in controlling the spatial distribution of sedimentation and enhancing riverbed stability in the downstream reach of bends. As a result, buried pipe installation, particularly under the hydrograph with Tr/Td=0.75, resulted in the greatest reduction in the maximum scour and deposition percentages. This behavior indicates that proper pipe burial can enhance bed stability in the downstream reach of the bend during flood flows by reducing topographic changes. From a river engineering perspective, these results can be used to determine the appropriate burial depth for pipelines crossing rivers and to reduce the risk of scour and pipe instability caused by bed morphological changes under flood conditions.

**Table:** 

• Required Symbols	
ds _max_: Maximum scour depth	h_s max_: Maximum sedimentation height
T_r_/T_d_: Ratio of time to peak discharge to duration time	D: Pipe diameter
X: Cross-stream position	Y: position along the straight reach
a: above the bed condition	b: bed level condition
c: buried condition	Z: Bed elevation change
α, β: equation coefficients	

## Supporting information

S1 File Fee Waiver. (RAR)
