# Supplementary material for: Investigation of the effect of outflow from a sharp bend, influenced by a pipe installed at the bend exit, on the scour pattern in the downstream straight reach under unsteady flow conditions
Source: PLoS One. 2026 Oct 27;21(10):e0360062. doi: 10.1371/journal.pone.0360062 (PMC13712536; doi:10.1371/journal.pone.0360062)
Supplement: Supplementary file 1 [file pone.0360062.s002.pdf]

These comments are very important to improve the quality of the paper

No.	Comment
ABSTRACT	
1	Clearly state the research gap in the abstract and explain why the downstream reach of a 180-degree sharp bend requires further investigation compared with previous studies.
2	Include a brief description of the experimental program, particularly the tested pipeline installation conditions and the range of unsteady-flow scenarios considered in the study.
3	Specify the main experimental parameters and measurement variables used to evaluate scour development and bed topography changes.
4	Expand the practical significance of the findings by briefly explaining how the reported scour reduction can support safer pipeline installation and river engineering applications.
INTRODUCTION	
5	Strengthen the critical discussion of prior studies by more explicitly highlighting their limitations and clarifying how the present study addresses these gaps.
6	Include a more comprehensive comparison between studies conducted in straight channels and those performed in channel bends to better justify the selection of the 180-degree sharp bend configuration.
7	Expand the novelty statement by clearly distinguishing the present work's contribution from previous investigations of pipeline scour under unsteady flow conditions and across different installation configurations.

LITERATURE REVIEW	
8	The discussion presented in Lines 64–75 focuses on unsteady-flow studies; however, most of the cited works investigate bridge pier scour rather than pipeline scour. It is recommended to include additional recent studies that specifically address pipeline scour under unsteady flow conditions to strengthen the relevance of this section.
9	The section on pipeline-related studies (Lines 80–154) provides a chronological summary of prior work. However, a comparative discussion highlighting the similarities and differences among the reported findings would enhance the review's scientific depth and more effectively justify the selected experimental variables.
10	The review includes several studies on scour around pipelines in straight channels, whereas limited attention is given to studies conducted in channel bends. Expanding the discussion on bend-related scour processes would provide a stronger foundation for the selected 180-degree bend configuration.
11	The novelty statement presented in Lines 155–164 could be strengthened by directly linking the identified limitations of previous studies to the objectives of the current research. A concise summary table comparing previous investigations with the present study may help clarify the research contribution.
PROPOSED THEORETICAL FRAMEWORK	
12	In Section 3.2, the mapping of de Bono's tools to architectural design stages is conceptually clear; however, the framework would benefit from a justification for selecting all four tools (Six Thinking Hats, Six Frames, Six Value Medals, and Six Action Shoes). Please explain why these tools were chosen over other cognitive or design-thinking approaches and how each contributes uniquely to the framework.

13	Table 1 provides a descriptive mapping between de Bono's tools and AI-assisted workflows; however, the operational procedure remains unclear. Consider adding a practical example that illustrates how a designer moves from the "cognitive structuring" stage to the "AI interaction" stage, including the roles of prompts, evaluation criteria, and decision-making.
14	In Section 3.3, the proposed five-layer model (input, cognitive structuring, AI interaction, evaluation, and decision-making) is presented at a conceptual level. Please clarify the specific methods, tools, or assessment mechanisms used within each layer to demonstrate that the framework is sufficiently comprehensive, reproducible, and applicable in real architectural design workflows.
RESEARCH METHOD AND CASE STUDY	
15	In Section 4.1, the study states that it adopts a qualitative analytical approach using a case study method; however, the methodological procedure remains broad. Please clarify the exact steps used to compare the unstructured AI-assisted design with the structured de Bono-based process, including how consistency between the two modes was controlled.
16	In Sections 4.3 and 4.4, the comparison between Mode A and Mode B is important, but the evaluation process needs more detail. Please explain how the expert review comments were collected, how many experts were involved, what criteria they used, and whether the evaluation criteria in Table 3 were applied equally to both design modes.
RESULTS AND DISCUSSION	
17	The discussion section would be strengthened by providing a more in-depth comparison between the findings obtained and those reported in previous studies. In particular, the similarities and differences in the effectiveness of the structured de Bono-based approach should be critically discussed and supported by relevant literature, rather than relying primarily on descriptive interpretations of the results.

18	The results demonstrate the advantages of the proposed framework; however, the discussion should further explain the underlying reasons behind the observed improvements in design quality, coherence, and decision-making. Linking these findings to established theories of human–AI collaboration and cognitive structuring would enhance the discussion's scientific value and improve the interpretation of the results.
CONCLUSIONS	
19	The conclusions section effectively summarizes the main findings; however, it would benefit from a clearer distinction between the study findings, practical implications, and future research directions. This would improve the overall structure and enhance the study's contribution to both research and practice.
20	Several conclusions are presented as general statements. Consider linking each major conclusion more explicitly to the corresponding results and discussion findings, and indicate how the proposed framework advances current knowledge in AI-assisted architectural design compared with existing approaches.
GENERAL COMMENTS	
• Please improve the captions of the figures and tables by making them more self-explanatory. The reader should be able to understand the purpose of each figure or table without referring extensively to the main text.<br>• Although the novelty of the study is mentioned, a concise subsection summarizing the theoretical and practical contributions of the proposed framework would help readers better understand its added value compared with existing human–AI collaboration approaches.<br>• The framework was evaluated through a single architectural case study. Consider discussing the framework's potential applicability to other architectural project types and design contexts to enhance its generalizability.<br>• The manuscript would benefit from a dedicated limitations subsection discussing potential constraints related to the selected case study, expert evaluation process, and AI-generated outputs.	

- A final proofreading of the manuscript is recommended to improve sentence structure, grammar, and consistency of terminology throughout the text.
