# Supplementary material for: Investigation of the effect of outflow from a sharp bend, influenced by a pipe installed at the bend exit, on the scour pattern in the downstream straight reach under unsteady flow conditions
Source: PLoS One. 2026 Oct 27;21(10):e0360062. doi: 10.1371/journal.pone.0360062 (PMC13712536; doi:10.1371/journal.pone.0360062)
Supplement: Peer review history

# Peer review: Decision Letter 0

**DOI:** 10.1371/journal.pone.0360062.r001

**Authors:** Kaywan Othman Ahmed

23 Jun 2026

Dear Dr. Vaghefi,

Thank you for submitting your manuscript to PLOS ONE. After careful consideration, we feel that it has merit but does not fully meet PLOS ONE’s publication criteria as it currently stands. Therefore, we invite you to submit a revised version of the manuscript that addresses the points raised during the review process.

Please submit your revised manuscript by Aug 22 2026 11:59PM. If you will need more time than this to complete your revisions, please reply to this message or contact the journal office at plosone@plos.org. When you're ready to submit your revision, log on to https://www.editorialmanager.com/pone/ and select the 'Submissions Needing Revision' folder to locate your manuscript file.

- A letter that responds to each point raised by the academic editor and reviewer(s). You should upload this letter as a separate file labeled 'Response to Reviewers'.
- A marked-up copy of your manuscript that highlights changes made to the original version. You should upload this as a separate file labeled 'Revised Manuscript with Track Changes'.
- An unmarked version of your revised paper without tracked changes. You should upload this as a separate file labeled 'Manuscript'.

If you would like to make changes to your financial disclosure, please include your updated statement in your cover letter. Guidelines for resubmitting your figure files are available below the reviewer comments at the end of this letter.

If applicable, we recommend that you deposit your laboratory protocols in protocols.io to enhance the reproducibility of your results. Protocols.io assigns your protocol its own identifier (DOI) so that it can be cited independently in the future. For instructions see: https://journals.plos.org/plosone/s/submission-guidelines#loc-laboratory-protocols. Additionally, PLOS ONE offers an option for publishing peer-reviewed Lab Protocol articles, which describe protocols hosted on protocols.io. Read more information on sharing protocols at https://plos.org/protocols?utm_medium=editorial-email&utm_source=authorletters&utm_campaign=protocols.

As the corresponding author, your ORCID iD is verified in the submission system and will appear in the published article. PLOS supports the use of ORCID, and we encourage all coauthors to register for an ORCID iD and use it as well. Please encourage your coauthors to verify their ORCID iD within the submission system before final acceptance, as unverified ORCID iDs will not appear in the published article. *Only* the individual author can complete the verification step; PLOS staff *cannot* verify ORCID iDs on behalf of authors.

We look forward to receiving your revised manuscript.

Kind regards,

Kaywan Othman Ahmed

Academic Editor

PLOS One

**Journal Requirements:**

When submitting your revision, we need you to address these additional requirements.

1. Please ensure that your manuscript meets PLOS ONE's style requirements, including those for file naming. The PLOS ONE style templates can be found at

https://journals.plos.org/plosone/s/file?id=wjVg/PLOSOne_formatting_sample_main_body.pdfand

https://journals.plos.org/plosone/s/file?id=ba62/PLOSOne_formatting_sample_title_authors_affiliations.pdf

2. Please provide a complete Data Availability Statement in the submission form, ensuring you include all necessary access information or a reason for why you are unable to make your data freely accessible. If your research concerns only data provided within your submission, please write "All data are in the manuscript and/or supporting information files" as your Data Availability Statement.

3. We note that Figure 1 in your submission contain copyrighted images. All PLOS content is published under the Creative Commons Attribution License (CC BY 4.0), which means that the manuscript, images, and Supporting Information files will be freely available online, and any third party is permitted to access, download, copy, distribute, and use these materials in any way, even commercially, with proper attribution. For more information, see our copyright guidelines: http://journals.plos.org/plosone/s/licenses-and-copyright.

We require you to either (1) present written permission from the copyright holder to publish these figures specifically under the CC BY 4.0 license, or (2) remove the figures from your submission:

1. You may seek permission from the original copyright holder of Figure(s) [#] to publish the content specifically under the CC BY 4.0 license.

We recommend that you contact the original copyright holder with the Content Permission Form (http://journals.plos.org/plosone/s/file?id=7c09/content-permission-form.pdf) and the following text:

“I request permission for the open-access journal PLOS ONE to publish XXX under the Creative Commons Attribution License (CCAL) CC BY 4.0 (http://creativecommons.org/licenses/by/4.0/). Please be aware that this license allows unrestricted use and distribution, even commercially, by third parties. Please reply and provide explicit written permission to publish XXX under a CC BY license and complete the attached form.”

Please upload the completed Content Permission Form or other proof of granted permissions as an "Other" file with your submission.

In the figure caption of the copyrighted figure, please include the following text: “Reprinted from [ref] under a CC BY license, with permission from [name of publisher], original copyright [original copyright year].”

2. If you are unable to obtain permission from the original copyright holder to publish these figures under the CC BY 4.0 license or if the copyright holder’s requirements are incompatible with the CC BY 4.0 license, please either i) remove the figure or ii) supply a replacement figure that complies with the CC BY 4.0 license. Please check copyright information on all replacement figures and update the figure caption with source information. If applicable, please specify in the figure caption text when a figure is similar but not identical to the original image and is therefore for illustrative purposes only.

4. Please upload a copy of Figure 5, to which you refer in your text on page 16. If the figure is no longer to be included as part of the submission please remove all reference to it within the text.

5. If the reviewer comments include a recommendation to cite specific previously published works, please review and evaluate these publications to determine whether they are relevant and should be cited. There is no requirement to cite these works unless the editor has indicated otherwise.

[Note: HTML markup is below. Please do not edit.]

Reviewers' comments:

Reviewer's Responses to Questions

**Comments to the Author**

1. Is the manuscript technically sound, and do the data support the conclusions?

Reviewer #1: Yes

Reviewer #2: Partly

Reviewer #3: Partly

**********

2. Has the statistical analysis been performed appropriately and rigorously?

Reviewer #1: Yes

Reviewer #2: Yes

Reviewer #3: N/A

**********

3. Have the authors made all data underlying the findings in their manuscript fully available?

The PLOS Data policy

Reviewer #1: Yes

Reviewer #2: Yes

Reviewer #3: No

**********

4. Is the manuscript presented in an intelligible fashion and written in standard English?

Reviewer #1: Yes

Reviewer #2: Yes

Reviewer #3: Yes

**********

Reviewer #1: The manuscript presents an interesting and relevant study on human–AI collaboration in architectural design. The topic is timely, and the proposed framework offers potential value for AI-assisted design processes. The manuscript is generally well structured and clearly written. However, several minor revisions are recommended to improve the clarity of the framework, strengthen the discussion, and further highlight the scientific contribution of the study. The authors are encouraged to carefully address all comments and revisions provided in the attached review table before the manuscript is considered for publication.

Reviewer #2: 1.Experimental details are generally described, yet several aspects require clarification to ensure reproducibility. Please provide more information on how the pipe was fixed relative to the mobile bed, how alignment along the streamwise direction was controlled, and whether any displacement or vibration occurred during the experiments.

2.The selection of a 2 m downstream straight reach as the primary analysis domain should be better justified. It is not entirely clear whether this length is sufficient to capture the full spatial development of scour holes and depositional patterns generated by bend-induced secondary flow and pipe–flow interaction.

3.Choice of the three pipe configurations (bed level, raised above bed, and buried) should be linked more explicitly to realistic field scenarios. Please explain whether the burial depths and exposure conditions represent typical riverine or engineering practice, and how sensitive the results might be to alternative embedment depths.

4.Sediment characteristics are reported as D 50 =1.5 mm with a given standard deviation, but additional information on gradation, uniformity, and preparation procedure is needed. Clarifying whether the sediment was sufficiently uniform to avoid armoring or selective transport would strengthen confidence in the interpretation of scour patterns.

5.To enrich the introduction and broaden the context of hydraulic control and water management strategies, it is recommended to discuss studies related to operational optimization in hydraulic systems; in this context, the study, Optimization of Flood Control in the Lake by Gate Opening Simultaneously by Kusmajaya et al., could provide useful complementary insight into hydraulic flow regulation and system management approaches that may be relevant to the broader discussion of flow behavior and control in engineered water systems.

6.Hydrograph design includes four rising-limb ratios (0.25, 0.50, 0.75, and 1.00 of total duration), yet the rationale for selecting these specific values is not fully explained. It would be helpful to clarify how these hydrographs relate to realistic flood events and whether peak discharge, total volume, and recession limbs were controlled consistently across cases.

7.Interpretation of the influence of peak timing on scour development would benefit from a clearer quantitative comparison among hydrographs. Please specify whether differences in scour depth arise primarily from peak magnitude, duration of high flow, cumulative shear stress, or interaction with bend-induced secondary currents.

8.The literature review needs to be strengthened, and incorporating additional recent studies on sediment and flow management in engineered waterways could be beneficial. In this regard, considering studies like, Designing a Multistage Flood Control Channel for Sediment and Flow Management in Coastal Areas by Mananoma et al., could help better position the present work within current research trends on integrated hydraulic design and sediment control in complex flow environments.

9.Measurement of bed topography using a laser distance meter with 5 cm by 2.5 cm grid spacing requires an uncertainty assessment. Reporting instrument accuracy, calibration procedures, and estimated measurement error would allow readers to evaluate the reliability of reported maximum scour depths and sedimentation volumes.

10.Preparation of the initial bed before each run is described as mechanical leveling, but it is unclear whether repeatability tests were conducted. Information on pre-wetting, consolidation time, and verification of identical starting conditions would strengthen the validity of comparisons among experiments.

11.The conclusion that pipe position has a stronger effect on scour reduction than unsteady-flow parameters is important but appears largely descriptive. A more systematic sensitivity or statistical analysis would provide stronger support for this claim and help quantify the relative contributions of geometric and hydraulic factors.

12.Mechanistic explanation of observed bed changes could be expanded by linking results more explicitly to known bend-flow phenomena such as helical motion, secondary circulation, flow separation, and redistribution of near-bed shear stress around the pipe. Providing velocity field measurements or referencing established theoretical frameworks would enhance the discussion.

Reviewer #3: Investigation of the Effect of Outflow from a Sharp Bend, Influenced by a Pipe Installed at the Bend Exit, on the Scour Pattern in the Downstream Straight Reach under Unsteady Flow Conditions

The manuscript presents an experimental laboratory study on scour and sedimentation patterns around a pipeline installed at the outlet of a 180-degree sharp bend under unsteady flow conditions. The topic falls within the scope of PLOS One, given its relevance to hydraulic engineering, sediment transport, and river morphology. The contents are in favor for researcher and hydraulic engineers. However, the manuscript in its current form is not merit to be published and requires major corrections before it can be considered for publication. The revised version need review.

Main comments

1. A significant portion of the manuscript appears to be generated by artificial intelligence. This is evident from repetitive phrasing, overly general statements, lack of critical analysis, and unnatural sentence structures throughout the text. The authors must thoroughly rewrite the manuscript in their own words to ensure originality, clarity, and scientific depth.

2. The manuscript lacks clear logical flow, it is some sections containing content that belongs elsewhere. The methodology and results and discussion sections should be seprated and divided in some headings and subheading and numbered so that each part be destigushed clearly. The manuscript lacks a clear logical flow throughout. To improve clarity and readability, the authors should restructure these sections by introducing appropriate headings and subheadings.

3. The results are presented only as tables and graphs with basic descriptions, but there is no deep analysis or interpretation of the findings. A dedicated discussion of results is missing. Please provide a thorough discussion that interprets the results, compares them with previous research, explains the physical mechanisms, and highlights the implications of the study.

Specific comments

4. The abstract is disproportionately focused on results, which occupy more than half of its length, while the methodology is described only in general terms. Also there are too many general statement in the abstract. Structure the abstract in this order: rationale → objective → methodology → key findings. Include quantitative results (e.g., 54% reduction in ..)

5. Revise Introduction section to focus on directly relevant literature, organized by theme and publication year. Highlight recent advancements and avoid general statements. End with a clear research objective distinct from the title/abstract. Extent to about 1.5 pages. There are useful papers in aceesjr.com journal to be used.

The methodology section is described too generally and lacks critical technical details needed for reproducibility. Specific issues include:

6. Insufficient detail regarding the experimental setup, including the dimensions and specifications of the flume, the grid resolution, and measurement techniques, the sediment size and experiments time (justifications), how the flow turbulent diminished and …. .

7. The rationale for selecting specific hydrograph parameters (peak discharge time ratios of 0.25, 0.50, 0.75, 1.00) is not justified.

8. The sediment properties and their critical shear stress values should be provided in more detail. Or stat the flow regime with proper description in the case of clear water and live bed condition, sediment uniformity, pipe instalation and any useful details for readers. As mention earlier, The content in the Materials and Methods section should be clearly separated and numbered. Each part must be distinct and presented under appropriate subheadings with proper numbering.

9. Include sufficient discussion of results in the "Results and Discussion" section in proper places in the text. Use prior studies to validate or justify your findings.

10. Avoid placing figures and tables consecutively. First describe the figure/table in the text, discuss its results, and then proceed to the next figure/table (like line 343).

11. The discussion section, where present, is underdeveloped. The authors should more comprehensively interpret their findings in the context of previous research, explaining similarities and differences and their implications for engineering practice.

12. The section on AI-generated content is particularly concerning; even some tables in the discussion section appear to show signs of artificial generation and must be rewritten by the authors in their own words.

13. Many statements in the results and conclusion sections are general and do not reflect a critical engagement with the data. For instance, the claim that "the installation condition played a decisive role" is not explored in terms of physical processes (e.g., pressure gradients, vortex dynamics, turbulence modulation).

14. The conclusion that hydrograph shape has "less effect than pipe burial depth" is not adequately justified. A comparative analysis of the relative influence of each parameter (e.g., normalized sensitivity or contribution analysis) would strengthen this assertion. As it stands, the conclusion appears to be based solely on the observation that the percentage reductions differ (37% vs. 13%), which may not be statistically meaningful.

15. The caption is mislabeled. There is no Fig. 5 in text.

**********

what does this mean?). If published, this will include your full peer review and any attached files.

If you choose “no”, your identity will remain anonymous but your review may still be made public.

**Do you want your identity to be public for this peer review?** For information about this choice, including consent withdrawal, please see our Privacy Policy

Reviewer #1: No

Reviewer #2: No

Reviewer #3: **Yes:**  Ata Amini

**********

[NOTE: If reviewer comments were submitted as an attachment file, they will be attached to this email and accessible via the submission site. Please log into your account, locate the manuscript record, and check for the action link "View Attachments". If this link does not appear, there are no attachment files.]

To ensure your figures meet our technical requirements, please review our figure guidelines: https://journals.plos.org/plosone/s/figures

You may also use PLOS’s free figure tool, NAAS, to help you prepare publication quality figures: https://journals.plos.org/plosone/s/figures#loc-tools-for-figure-preparation.

NAAS will assess whether your figures meet our technical requirements by comparing each figure against our figure specifications.

Attachment Submitted filename: Comment Table.pdf

# Author comment: Author response to Decision Letter 1

**DOI:** 10.1371/journal.pone.0360062.r002

7 Aug 2026

Dear Editor,

We would like to express our sincere appreciation for your thorough evaluation of our manuscript entitled "Investigation of the Effect of Outflow from a Sharp Bend, Influenced by a Pipe Installed at the Bend Exit, on the Scour Pattern in the Downstream Straight Reach under Unsteady Flow Conditions" bearing the manuscript number PONE-D-26-26226. We are grateful for the constructive feedback received from the reviewers. In response, we have made substantial revisions to our manuscript to bolster its scientific merit. We have diligently addressed each comment and suggestion provided by the reviewers and have emphasized these modifications in the revised manuscript. All recommendations have been addressed and highlighted (Separately for referees 1, 2 and 3 with Green, Red and Blue colors respectively) in the attached revised manuscript. We also initially answered questions raised by the associate editor (Journal Requirements). Because some comments were shared by both reviewers, the corresponding revisions have been highlighted in two different colors within the revised manuscript. However, in this response document, each comment has been addressed separately under the respective reviewer's section. To facilitate the review process, we have submitted two iterations of the revised manuscript: One marked with highlights to indicate the changes made (Revised Manuscript with changes marked) and another a final version of the manuscript (Not Highlighted Manuscript) uploaded. Furthermore, we have included a comprehensive point-by-point response to the reviewers' remarks, as outlined below.

We would like to take this opportunity to thank you once again for your thorough consideration that will surely improve the manuscript for publication.

Associate Editor (Journal Requirements):

Authors would like to appreciate your kindness and thoughtful comments. Also, the responses to your comments/suggestions are as follows. We would like to take this opportunity to thank you once again for your thorough consideration that will surely improve the manuscript.

Comment 1.

Please ensure that your manuscript meets PLOS ONE's style requirements, including those for file naming. The PLOS ONE style templates can be found at

https://journals.plos.org/plosone/s/file?id=wjVg/PLOSOne_formatting_sample_main_body.pdf and

https://journals.plos.org/plosone/s/file?id=ba62/PLOSOne_formatting_sample_title_authors_affiliations.pdf

Reply 1:

The provided links were thoroughly reviewed, and we have made every effort to ensure that all the specified requirements have been fully met.

Comment 2.

Please provide a complete Data Availability Statement in the submission form, ensuring you include all necessary access information or a reason for why you are unable to make your data freely accessible. If your research concerns only data provided within your submission, please write "All data are in the manuscript and/or supporting information files" as your Data Availability Statement.

Reply 2:

A Data Availability Statement was added at the end of the manuscript, immediately before the References section, as follows:

Availability of data and materials

All data generated or analysed during this study are included in this published article.

Comment 3.

We note that Figure 1 in your submission contain copyrighted images. All PLOS content is published under the Creative Commons Attribution License (CC BY 4.0), which means that the manuscript, images, and Supporting Information files will be freely available online, and any third party is permitted to access, download, copy, distribute, and use these materials in any way, even commercially, with proper attribution. For more information, see our copyright guidelines: http://journals.plos.org/plosone/s/licenses-and-copyright........................................

Reply 3:

All figures presented in the manuscript were created by the authors based on the research conducted in this study. Figure 1 is an original photograph of our laboratory setup, captured by the authors. It has not been reproduced or adapted from any other source and therefore does not require permission for publication.

Comment 4.

Please upload a copy of Figure 5, to which you refer in your text on page 16. If the figure is no longer to be included as part of the submission please remove all reference to it within the text.

Reply 4:

Regarding Figure 5, there was an error in the figure numbering, which has been corrected in the revised version of the manuscript.

Comment 5.

If the reviewer comments include a recommendation to cite specific previously published works, please review and evaluate these publications to determine whether they are relevant and should be cited. There is no requirement to cite these works unless the editor has indicated otherwise.

Reply 5:

Related references were added to the text of the article. We would also like to respectfully note that some of Reviewer 1's comments appear to pertain to a different manuscript or may not be directly applicable to the present study. Accordingly, we have clarified in our responses which comments we believe are not relevant to this manuscript.

Reviewer #1:

The authors investigated the changes in bed topography over time as a result of submersion in both the upstream side and the downstream areas of a culvert. They performed their experiment in a laboratory setting and considered instances of regular and internal blockage within the culvert. They found that the most significant decrease in the uttermost scour hole deepness happened amid full submersions. I found the laboratory experiment and results interesting; however, the presentation, structure and literature review should be improved. Please see below my comments.

Dear Reviewer,

Authors would like to appreciate your kindness and thoughtful comments. We have revised the manuscript as suggested per the given comments. All recommendations have been addressed and highlighted in Green color on the attached revised manuscript. Because some comments were shared by both reviewers, the corresponding revisions have been highlighted in two different colors within the revised manuscript. However, in this response document, each comment has been addressed separately under the respective reviewer's section. Also, the responses to your comments/suggestions are as follows. We would like to take this opportunity to thank you once again for your thorough consideration that will surely improve the manuscript.

Comment 1:

Clearly state the research gap in the abstract and explain why the downstream reach of a 180-degree sharp bend requires further investigation compared with previous studies.

Reply 1:

Thank you to the respected reviewer. To clarify the issue raised, the following paragraph has been added to the abstract of the manuscript. Please refer to the revised version of the paper.

“Although numerous studies have investigated scour around pipes in straight channels and bends, changes in bed topography along the straight reach downstream of a sharp bend, which is affected by the complex flow pattern issuing from the bend, have received less attention. Therefore, this study examines the effect of pipe installation at the end of a sharp bend and its influence on the downstream bed topography along the straight reach. In addition, from both an operational and hydraulic perspective, placing the pipe outside the bend would be a preferable option for designers and engineers.”

Comment2:

Include a brief description of the experimental program, particularly the tested pipeline installation conditions and the range of unsteady-flow scenarios considered in the study.

Reply 2:

We thank the respected reviewer for the careful observation. In line with this suggestion, a brief description of the experimental program has been added to the abstract of the manuscript. In the revised version, three pipe installation conditions relative to the bed level, the pipe placement arrangement, and four unsteady-flow scenarios based on different ratios of peak-flow timing to flood duration are briefly stated in the abstract. Accordingly, the first paragraph has been added to the abstract. In addition, to clarify the different unsteady-flow hydrograph scenarios, the subsequent paragraphs have been added to Section 2.3 of the Materials and Methods.

“The experiments were conducted for three pipe-installation conditions relative to the bed level, namely level with the bed, above the bed, and buried, with the pipe positioned perpendicular to the flow direction and under four different ratios of hydrograph peak timing to flood duration in unsteady flow.”

“In all four hydrographs used in these experiments, the base discharge, peak discharge, and duration time were kept constant, and the only variable parameter was the timing of the peak discharge relative to the duration time. This parameter was defined by four different ratios in order to investigate the role of the timing of the peak discharge relative to flood duration in the scour and deposition process. In the hydrograph with Tr/Td=0.25, the discharge reaches its peak during the first quarter of the experiment and then gradually returns to the base discharge, representing a flood with a sudden peak under real conditions. In the hydrograph with Tr/Td=0.50, the rising and falling limbs are equal, and the peak discharge occurs at mid-duration, representing an approximately normal flood. In the hydrograph with Tr/Td=0.75, the discharge reaches its peak after three quarters of the experimental duration and then returns to the base discharge, representing a gradual flood with a delayed peak. In the hydrograph with Tr/Td=1.0, the discharge increases gradually from the base discharge to the peak discharge, representing the rise of rainfall to its maximum followed by its abrupt cessation.”

Comment 3:

Specify the main experimental parameters and measurement variables used to evaluate scour development and bed topography changes.

Reply 3:

We thank the respected reviewer for the constructive suggestion. To make the abstract clearer, the main variables used to evaluate the results were also added. Accordingly, the following paragraph has been added to the abstract. Please refer to the revised version of the manuscript.

“To evaluate the effects of different pipe installation conditions as well as the flow hydrograph, parameters such as the extent of scour-hole development, the maximum scour depth, the maximum deposition height, and the bed topography changes in the straight reach downstream of the bend were measured and analyzed.”

Comment 4:

Expand the practical significance of the findings by briefly explaining how the reported scour reduction can support safer pipeline installation and river engineering applications.

Reply 4:

We thank the respected reviewer for the valuable comment. In line with the suggestion, and to better clarify the practical significance of the findings, the first paragraph has been added to the end of the abstract and the second paragraph to the end of the Results section. Please refer to the revised version of the manuscript.

“The findings of this study can be used to identify the most suitable pipe installation condition in the straight reach downstream of sharp bends, reduce scour risk, predict bed changes, and improve the design and protection of pipelines in river engineering projects.”

“As a result, buried pipe installation, particularly under the hydrograph with Tr/Td=0.75, resulted in the greatest reduction in the maximum scour and deposition percentages. This behavior indicates that proper pipe burial can enhance bed stability in the downstream reach of the bend during flood flows by reducing topographic changes. From a river engineering perspective, these results can be used to determine the appropriate burial depth for pipelines crossing rivers and to reduce the risk of scour and pipe instability caused by bed morphological changes under flood conditions.”

Comment 5:

Strengthen the critical discussion of prior studies by more explicitly highlighting their limitations and clarifying how the present study addresses these gaps.

Reply 5:

We thank the respected reviewer for the valuable comment. In accordance with the suggestion, the Introduction section has been revised and strengthened. In the revised manuscript, in addition to reviewing previous studies, the limitations and research gaps in the existing literature have been discussed more clearly. In this regard, the following paragraph has been added to the Introduction.

“A review and comparison of previous studies indicates that, although the role of factors such as hydraulic conditions, sediment characteristics, and pipe installation conditions in the initiation and development of scour around pipelines is well recognized, most studies have examined these effects independently. For example, some studies have focused on the influence of pipe burial depth or installation configuration, others on hydraulic parameters such as flow velocity, Froude number, and the angle of flow incidence on the pipe, and only a limited number have addressed unsteady flow conditions. Moreover, most of these studies have been conducted in straight channels or within the bend itself, while the morphodynamic behavior of the bed in the straight reach downstream of the bend, which is directly affected by the flow issuing from the bend, has received less attention. Therefore, despite a sound understanding of the independent effects of the factors influencing scour, available information on the combined effects of unsteady flow conditions and different pipe installation configurations on scour development and bed topographic changes in this region remains limited. Accordingly, these two factors were selected as the main variables of the present study to evaluate their interactive effects on topographic changes in the straight reach downstream of the bend.”

Comment 6:

Include a more comprehensive comparison between studies conducted in straight channels and those performed in channel bends to better justify the selection of the 180-degree sharp bend configuration.

Reply 6:

We thank the respected reviewer for the valuable suggestion. The Introduction section has been revised and corrected. The revised version was strengthened by adding a comprehensive comparison between studies conducted in straight channels and river bends, so that the reason for selecting the straight reach downstream of the 180-degree bend and the importance of examining the bed behavior in this reach are made clearer. In this regard, the following paragraph has been added to the end of the literature review in the manuscript.

“Studies conducted in straight channels have clearly demonstrated the role of hydraulic and geometric factors in the development of scour around pipelines; however, the conditions prevailing in river bends are more complex due to the formation of secondary currents induced by centrifugal force and changes in the distribution of velocity and shear stress. These processes cause lateral sediment transport and, consequently, alter the scour and deposition patterns compared with straight channels. The downstream reaches of sharp river bends are among the most morphodynamically sensitive zones, because the flow exiting the bend retains part of its three-dimensional structure, affects the hydrodynamic and morphodynamic behavior of the bed in this region, and increases the likelihood of scour around hydraulic structures. Therefore, generalizing the results of studies conducted in straight channels to this region is limited, and the independent investigation of this reach, particularly in the presence of crossing pipelines, is of special importance.”

Comment 7:

Expand the novelty statement by clearly distinguishing the present work's contribution from previous investigations of pipeline scour under unsteady flow conditions and across different installation configurations.

Reply 7:

We thank the respected reviewer for the valuable suggestion. In the revised manuscript, the research novelty section has been rewritten and completed. In this section, the differences between the present study and previous studies have been clearly stated. Accordingly, the following paragraph has been added to the end of the Introduction.

“The novelty of this study lies in providing an integrated experimental assessment of the morphodynamic response of the bed in the straight reach downstream of a 180-degree sharp bend in the presence of a crossing pipeline under unsteady flow conditions. In this study, the simultaneous effects of different pipe installation conditions and different peak-flow timing ratios in the unsteady-flow hydrograph on scour development, deposition pattern, and bed topographic changes were experimentally investigated. This approach enables a better understanding of bed behavior under conditions closer to those found in natural rivers and can provide a basis for safer design, placement, and management of crossing pipelines downstream of sharp bends.”

Comment 8:

The discussion presented in Lines 64–75 focuses on unsteady-flow studies; however, most of the cited works investigate bridge pier scour rather than pipeline scour. It is recommended to include additional recent studies that specifically address pipeline scour under unsteady flow conditions to strengthen the relevance of this section.

Reply 8:

We thank the respected reviewer for the careful observation. In order to create greater coherence in the literature review, this section has been organized into three parts: studies related to river bends, studies related to unsteady flows and their effects on sediment transport, bed changes, and flood management, and finally studies related to scour around pipelines. Therefore, in the section in question, the focus was on research related to unsteady flows and their effects, while studies on scour around pipelines, including those conducted under unsteady flow conditions, were reviewed and compared separately in the subsequent part of the literature review. To avoid any misunderstanding, the following paragraph was added to the end of the subsection on unsteady-flow studies to clearly indicate its connection to the pipeline-studies subsection. In addition, further studies were added to enrich the literature review.

“Given that this subsection focuses on the behavior of unsteady flows, sediment transport, and bed changes, the studies related to scour around pipelines under these conditions have been reviewed separately in the next section in order to maintain coherence.”

The following paragraphs provide the descriptions of the articles added to the literature review section.

“Flows, in terms of temporal variability, are classified into steady and unsteady types. Given the temporal variations in flow depth and velocity inherent to the unsteady flow type, the present study focused on examining this class of flows. Accordingly, previous studies and published works related to the behavior of unsteady flows and their effects on flood management and bed changes are reviewed in the following sections.”

“Wang et al. (2019) in this experimental study, the evolution of bed morphology due to bed load transport under unsteady hydrograph flows was investigated. The results indicate that the magnitude and unsteadiness of the hydrograph have significant impacts on sediment transport rates and yields, as well as on the hysteresis patterns and yield ratios generated during the rising and falling limbs of the hydrograph”.

Wang L, Cuthbertson A, Pender G, Zhong D. Bed load sediment transport and morphological evolution in a degrading uniform sediment channel under unsteady flow hydrographs. Water Resources Research. 2019; 55(7), 5431-5452.‏ http://dx.doi.org/10.1029/2018WR024413

“Cahyani et al. (2022) a study was conducted on the Cengkareng Floodway to investigate the impact of sediment transport on bed morphological changes. Utilizing a Quasi-Unsteady Flow model within the HEC-RAS 1D software and accounting for tidal boundary conditions, the researchers simulated the sedimentation rates “.

Cahyani R, Cahyono M, Eliasta L, Setiadi A, Moerwanto E. Analysis of The Effect Of Sediment Transport on River Bed Changes of Cengkareng Floodway. 2022.

“Mananoma et al. (2025) Regarding the management of flood flows and sediment transport, the researchers designed a multi-stage channel profile using HEC-HMS and HEC-RAS models to control flooding and mitigate the adverse impacts of sediment movement”.

Mananoma T, Sumarauw J, Legrans R, Ratu Y. Designing a multistage flood control channel for sediment and flow management in coastal areas. J. Hum. Earth Future. 2025; 6(3), 654-662.‏ http://dx.doi.org/10.28991/HEF-2025-06-03-011

"Kusmajaya et al. (2025) Regarding flood risk management, this study investigates the optimization of Limboto Lake operations as a flood retention basin. By employing optimization techniques and simulating various operational scenarios for the Tapodu Canal gates, the research demonstrates that appropriate gate regulation can significantly reduce peak discharge during high-return-period flood events, effectively preventing overflow in the downstream canal."

Kusmajaya F, Limantara L, Yuliani E, Soetopo W. Optimization of Flood Control in the Lake by Gate Opening Simultaneously. Journal of Human, Earth, and Future. 2025; 6(1), 150-165. http://dx.doi.org/10. 28991/HEF-2025-06-01-010.

Comment 9:

The section on pipeline-related studies (Lines 80–154) provides a chronological summary of prior work. However, a comparative discussion highlighting the similarities and differences among the reported findings would enhance the review's scientific depth and more effectively justify the selected experimental variables.

Reply 9:

We thank the respected reviewer for the valuable comment. Given the overlap between this comment and Comment 5, an effort was made to address both points in a unified manner and within a single paragraph so as to avoid repetition and to present the logical connection among the critique of previous studies, comparison of their results, and clarification of the research gap more clearly. In the revised version, an analytical and comparative paragraph has been added to the end of the literature review to clearly explain the rationale for selecting the investigated parameters, with emphasis on the existing gaps in previous studies.

Comment 10:

The review includes several studies on scour around pipelines in straight channels, whereas limited attention is given to studies conducted in channel bends. Expanding the discussion on bend-related scour processes would provide a stronger foundation for the selected 180-degree bend configuration.

Reply 10:

We thank the respected reviewer for the valuable comment. Given the overlap between this comment and Comment 6, an effort was made to address both points in a unified manner and within a single paragraph so as to avoid repetition. In the revised version, the discussion of scour processes in river bends was expanded, and the roles of secondary currents, centrifugal force, and changes in the distribution of velocity and shear stress in shaping scour and deposition patterns in bends were described in greater detail. This clarifies the importance of the straight reach downstream of the bend and the reason for selecting this reach as the study domain. It should also be noted that, in the present study, the pipeline is located precisely in the straight reach downstream of the bend. Accordingly, the main part of the literature review was devoted to studies on pipeline scour in straight channels. However, because the flow behavior and morphological changes in this reach are directly influenced by the flow exiting the 180-degree bend, some studies on pipelines in bends were also reviewed. In addition, following your comment, studies on scour in bends were added to the literature review. As you know, most studies on scour in bends focus on structures located within the bend, and fewer studies have examined scour in the bend alone; nevertheless, the following paragraph was added to the literature review as an example.

“Sui et al. (2006) and Biswas & Barbhuiya (2020) investigated scour in river bends with 90- and 180-degree angles and, in their studies, examined the parameters affecting scour as well as changes in the bed topography of the bend.”

Sui J, Fang D, & Karney BW. An experimental study into local scour in a channel caused by a 90 bend. Canadian Journal of Civil Engineering. 2006;33(7), 902-911. https://doi.org/10.1139/l06-037 . ‏

Biswas P, Barbhuiya AK. Scour at River Bend: A Parametric Study. Iran J Sci Technol Trans Civ Eng 2020.;44, 1001–1021. https://doi.org/10.1007/s40996-020-00414-9

Comment 11:

The novelty statement presented in Lines 155–164 could be strengthened by directly linking the identified limitations of previous studies to the objectives of the current research. A concise summary table comparing previous investigations with the present study may help clarify the research contribution.

Reply 11:

We thank the respected reviewer for the valuable comment. Given the overlap between this comment and the previous ones, the literature review and the statement of the research novelty were thoroughly revised. In the revised version, the differences between studies conducted in straight channels and river bends were first described; then, the limitations and gaps in the previous studies were stated more clearly; and finally, the direct connection between these gaps and the objectives and novelty of the present study was explained. Since presenting a comparative table in the literature review section is not customary, and with your permission, such a table was not added to the manuscript, and a comprehensive textual explanation was provided instead. However, for your reference, the table below was prepared to compare the characteristics of selected previous studies with those of the present study and to clearly show the differences and the scientific contribution of the present work.

Main focus of the study Study environment Flow condition Study

Investigation of the flow field and scour mechanisms around a pipeline Straight channel Steady Flow Wu & Chiew (2013)

Investigation of the effects of pipe burial depth, flow angle of incidence, and hydraulic on scour Straight channel Steady Flow Cheng et al. (2014)

Investigation of the effects of pipe diameter and different installation configurations Straight channel Unsteady Flow Bijanvand et al. (2021)

Investigation of the effects of pipe angle and apron on the scour and sedimentation patterns 90-degree bend Steady Flow Valizadeh et al. (2023)

Simultaneous investigation of the effects of bend conditions, pipe installation configurations, and different unsteady-flow scenarios on scour around a pipeline Straight reach downstream of a 180-degree sharp bend Unsteady Flow Present study

Comment 12:

In Section 3.2, the mapping of de Bono’s tools to architectural design stages is conceptually clear; however, the framework would benefit from a justification for selecting all four tools (Six Thinking Hats, Six Frames, Six Value Medals, and Six Action Shoes). Please explain why these tools were chosen over other cognitive or design-thinking approaches and how each contributes uniquely to the framework.

Reply 12:

Thank you for your comment. It appears that this comment refers to a paper with a different topic and is not consistent with the subject, structure, and content of the present study, which focuses on scour around pipelines downstream of sharp bends.

Comment 13:

Table 1 provides a descriptive mapping between de Bono’s tools and AI-assisted workflows; however, the operational procedure remains unclear. Consider adding a practical example that illustrates how a designer moves from the “cognitive structuring” stage to the “AI interaction” stage, including the roles of prompts, evaluation criteria, and decision-making.

Reply 13:

With thanks and respect, it appears that the issue raised in this comment is not directly related to the present study, as the current paper investigates, experimentally, scour and bed topographic changes around pipelines in the straight reach downstream of a 180-degree sharp bend under unsteady flow conditions.

Comment 14:

In Section 3.3, the proposed five-layer model (input, cognitive structuring, AI interaction, evaluation, and decision-making) is presented at a conceptual level. Please clarify the specific methods, tools, or assessment mechanisms used within each layer to demonstrate that the framework is sufficiently comprehensive, reproducible, and applicable in real architectural design workflows.

Reply 14:

With thanks and respect, this comment appears to relate to a topic other than the present manuscript and does not align with the objectives, methodology, or scope of the study presented here.

Comment 15:

In Section 4.1, the study states that it adopts a qualitative analytical approach using a case study method; however, the methodological procedure remains broad. Please clarify the exact steps used to compare the unstructured AI-assisted design with the structured de Bono-based process, including how consistency between the two modes was controlled.

Reply 15:

With thanks and respect, it appears that the issue raised in this comment is not directly related to the present study, as the current paper investigates, experimentally, scour and bed topographic changes around pipelines in the straight reach downstream of a 180-degree sharp bend under unsteady flow conditions.

Comment 16:

In Sections 4.3 and 4.4, the comparison between Mode A and Mode B is important, but the evaluation process needs more detail. Please explain how the expert review comments were collected, how many experts were involved, what criteria they used, and whether the evaluation criteria in Table 3 were applied equally to both design modes.

Reply 16:

With thanks and respect, it appears that the issue raised in this comment is not directly related to the present study, as the current paper investigates, experimentally, scour and bed topographic changes around pipelines in the straight reach downstream of a 180-degree sharp bend under unsteady flow conditions.

Comment 17:

The discussion section would be strengthened by providing a more in-depth comparison between the findings obtained and those reported in previous studies. In particular, the similarities and differences in the effectiveness of the structured de Bono-based approach should be critically discussed and supported by relevant literature, rather than relying primarily on descriptive interpretations of the results.

Reply 17:

With thanks and respect, this comment appears to relate to a topic other than the present manuscript and does not align with the objectives, methodology, or scope of the study presented here.

Comment 18:

The results demonstrate the advantages of the proposed framework; however, the discussion should further explain the underlying reasons behind the observed improvements in design quality, coherence, and decision-making. Linking these findings to established theories of human–AI collaboration and cognitive structuring would enhance the discussion's scientific value and improve the interpretation of the results.

Reply 18:

With thanks and respect, it appears that the issue raised in this comment is not related to the present study.

Comment 19:

The conclusions section effectively summarizes the main findings; however, it would benefit from a clearer distinction between the study findings, practical implications, and future research directions. This would improve the overall structure and enhance the study's contribution to both research and practice.

Reply 19:

With thanks and respect, it appears that the issue raised in this comment is not directly related to the present study, as the current paper investigates, experimentally, scour and bed topographic changes around pipelines in the straight reach downstream of a 180-degree sharp bend under unsteady flow conditions.

Comment 20:

Several conclusions are presented as general statements. Consider linking each major conclusion more explicitly to the corresponding results and discussion findings, and indicate how the proposed framework advances current knowledge in AI-assisted architectural design compared with existing approaches.

Reply 20:

With thanks and respect, this comment appears to relate to a topic other than the present manuscript and does not align with the objectives, methodology, or scope of the study presented here.

Comment 21:

Please improve the captions of the figures and tables by making them more self-explanatory. The reader should be able to understand the purpose of each figure or table without referring extensively to the main text.

Reply 21:

With thanks and respect, it appears that the issue raised in this comment is not related to the present study.

Comment 22:

Although the novelty of the study is mentioned, a concise subsection summarizing the theoretical and practical contributions of the proposed framework would help readers better understand its added value compared with existing human–AI collaboration approaches.

Reply 22:

With thanks and respect, it appears that the issue raised in this comment is not related to the present study.

Comment 23:

The framework was evaluated through a single architectural case study. Consider discussing the framework's potential applicability to other architectural project types and design contexts to enhance its generalizability.

Reply 23:

With thanks and respect, it appears that the issue raised in this comment is not related to the present study.

Comment 24:

The manuscript would benefit from a dedicated limitations subsection discussing potential constraints related to the selected case study, expert evaluation process, and AI-generated outputs.

Reply 24:

With thanks and respect, it appears that the issue raised in this comment is not related to the present study.

Comment 25:

A final proofreading of the manuscript is recommended to improve sentence structure, grammar, and consistency of terminology throughout the text.

Reply 25:

With thanks and respect, it appears that the issue raised in this comment is not related to the present study.

Reviewer #2:

Dear Reviewer,

Authors would like to appreciate your kindness and thoughtful comments. We have revised the manuscript as suggested per the given comments. All recommendations have been addressed and highlighted in Red color on the attached revised manuscript. Because some comments were shared by both reviewers, the corresponding revisions have been highlighted in two different colors within the revised manuscript. However, in this response document, each comment has been addressed separately under the respective reviewer's section. Also, the responses to your comments/suggestions are as follows. We would like to take this opportunity to thank you once again for your thorough consideration that will surely improve the manuscript.

Comment 1:

Experimental details are generally described, yet several aspects require clarification to ensure reproducibility. Please provide more information on how the pipe was fixed relative to the mobile bed, how alignment along the streamwise direction was controlled, and whether any displacement or vibration occurred during the experiments.

Reply 1:

Thank you for your valuable comment. To clarify the issue raised, the following paragraph added to the manuscript in Section 2-2, Materials and Methods. Please see the revised version of the paper.

“Before each experiment, the pipe was positioned at the desired distance from the bed, perpendicular to the flow direction and across the channel width. To ensure a constant pipe clearance from the initial bed, a level gauge was used. After verifying the correct alignment, the pipe was fixed to both channel walls using transparent aquarium sealant. The sealant was applied carefully so as not to induce flow distortion or additional roughness effects, and thus to prevent any wall-induced deflection of the flow. Throughout the experiments, the pipe remained completely stationary, with no vibration or displacement, and behaved as a rigid body oriented perpendicular to the flow. In addition, a mechanical bed-leveling device was used to prepare the initial bed. This device was mounted on rails installed along the top of the channel walls, with the same slope as the longitudinal bed slope of the channel. At the start of each test, the device was moved along the channel to produce an initial bed with a uniform longitudinal slope.”

Comment 2:

The selection of a 2 m downstream straight reach as the primary analysis domain should be better justified. It is not entirely clear whether this length is sufficient to capture the full spatial development of scour holes and depositional patterns generated by bend-induced secondary flow and pipe–flow interaction

Reply 2:

Thank you for your careful review and insightful comment. The selection of this analysis range for bed-change assessment was based on experimental observations and the bed topography results obtained from the preliminary tests. As also shown in the provided topographic images, the scour and deposition caused by the interaction of the flow exiting the bend with the pipe were observed up to approximately 1.5 m downstream of the pipe, while in the subsequent 0.5 m the bed changes were nearly uniform and negligible, and the bed topography pattern had reached a stable state. Nevertheless, a 2 m downstream reach from the pipe was considered in this study. Accordingly, the following paragraph was added to the manuscript in Section 2-2, Materials and Methods:

“Given that preliminary experiments showed noticeable changes in the bed topography up to 1.5 m downstream of the end of the bend, the bed topography along the straight downstream reach was measured up to 2 m to investigate the effect of the interaction between the flow exiting the bend and the pipe oriented perpendicular to the unsteady flow path.”

Comment 3:

Choice of the three pipe configurations (bed level, raised above bed, and buried) should be linked more explicitly to realistic field scenarios. Please explain whether the burial depths and exposure conditions represent typical riverine or engineering practice, and how sensitive the results might be to alternative embedment depths

Reply 3:

Thank you for your valuable point of view. The three selected configurations were chosen to simulate the common and practical conditions of pipeline installation in riverbeds and to investigate scour under different conditions of interaction between unsteady flow and the pipe. Certainly, the selected pipe-installation elevations relative to the initial bed can play an important role in the scour results in the downstream straight reach. Accordingly, in this study and considering the limitations associated with the research timeframe, the three elevations mentioned in the paper were selected with different characteristics and purposes. In addition, to clarify the issue raised, the following paragraph added to the manuscript in Section 2-2, Materials and Methods. Please see the revised version of the manuscript.

“With regard to selecting the installation level of the pipe perpendicular to the unsteady flow relative to the initial bed, the buried condition represents the conventional method of protecting pipelines by embedding them within the bed, thereby reducing direct flow–pipe interaction. The level with the bed condition refers to the situation in which scour around the pipe has progressed to a depth equal to the pipe diameter; in other words, the pipe has become exposed at the level of the riverbed over time, or, due to construction constraints, it has been installed flush with the bed. The above the bed condition indicates a state in which severe flooding has caused deep scour around the pipe, and in subsequent floods the pipe is positioned above the initial bed. In this condition, part of the flow passes beneath the pipe and part passes over it, while the pipe itself acts as an obstacle in the flow path. Since each of these three pipe-installation conditions relative to the initial bed affects the bed differently under unsteady flow, these three installation levels were selected relative to the initial bed.”

Comment 4:

Sediment characteristics are reported as D 50 =1.5 mm with a given standard deviation, but additional information on gradation, uniformity, and preparation procedure is needed. Clarifying whether the sediment was sufficiently uniform to avoid armoring or selective transport would strengthen confidence in the interpretation of scour patterns

Reply 4:

Thank you for your valuable comment. According to Melville and Chiew (1987), if the standard deviation of the bed material particles is less than 1.3, the effect of particle non-uniformity on scour development is negligible. In this study, the siliceous bed material had a particle diameter of 1.5 mm and a standard deviation of 1.14. Therefore, these materials fall within a uniformity range in which the effects of armoring and selective transport are minimized. It should be noted that all bed materials were sieved before the experiments, and the particle size distribution curve indicated particle uniformity. The reported parameters were also obtained based on the grain-size analysis of the bed materials. Accordingly, the following paragraph was added to the manuscript in Section 2-2, Materials and Methods. Please see the revised version of the manuscript.

“According to Melville and Chiew (1987), if the standard deviation of the bed material particles is less than 1.3, the effect of particle non-uniformity on scour development is negligible. In the present study, the standard deviation of the bed particles was 1.14. Therefore, these materials fall within a uniformity range in which armoring and selective transport effects are minimized.”

Comment 5:

To enrich the introduction and broaden the context of hydraulic control and water management strategies, it is recommended to discuss studies related to operational optimization in hydraulic systems; in this context, the study, Optimization of Flood Control in the Lake by Gate Opening Simultaneously by Kusmajaya et al., could provide useful complementary insight into hydraulic flow regulation and system management approaches that may be relevant to the broader discussion of flow behavior and control in engineered water systems.

Reply 5:

According to your suggestion, the relevant content has been added to the Introduction section of the manuscript. Please see the revised version of the manuscript.

Kusmajaya et al. (2025) Regarding flood risk management, this study investigates the optimization of Limboto Lake operations as a flood retention basin. By employing optimization techniques and simulating various operational scenarios for the Tapodu Canal gates, the research demonstrates that appropriate gate regulation can significantly reduce peak discharge during high-return-period flood events, effectively preventing overflow in the downstream canal [12].

Kusmajaya F, Limantara L, Yuliani E, Soetopo W. Optimization of Flood Control in the Lake by Gate Opening Simultaneously. Journal of Human, Earth, and Future. 2025;6(1), 150-165. http://dx.doi.org/10. 28991/HEF-2025-06-01-010

Comment 6:

Hydrograph design includes four rising-limb ratios (0.25, 0.50, 0.75, and 1.00 of total duration), yet the rationale for selecting these specific values is not fully explained. It would be helpful to clarify how these hydrographs relate to realistic flood events and whether peak discharge, total volume, and recession limbs were controlled consistently across cases.

Reply 6:

Thank you for your valuable comment. To clarify the issue raised, the following paragraph was added to the manuscript in Section 2-3, Materials and Methods. Please see the revised version of the paper.

“In all four hydrographs used in these experiments, the base discharge, peak discharge, and duration time were kept constant, and the only variable parameter was the timing of the peak discharge relative to the duration time. This parameter was defined by four different ratios in order to investigate the role of the timing of the peak discharge relative to flood duration in the scour and deposition process. In the hydrograph with Tr/Td=0.25, the discharge reaches its peak during the first quarter of the experiment and then gradually returns to the base discharge, representing a flood with a sudden peak under real conditions. In the hydrograph with Tr/Td=0.50, the rising and falling limbs are equal, and the peak discharge occurs at mid-duration, representing an approximately normal flood. In the hydrograph with Tr/Td=0.75, the discharge reaches its peak after three quarters of the experimental duration and then returns to the base discharge, representing a gradual flood with a delayed peak. In the hydrograph with Tr/Td=1.0, the discharge increases gradually from the base discharge to the peak discharge, representing the rise of rainfall to its maximum followed by its abrupt cessation.”

Comment 7:

Interpretation of the influence of peak timing on scour development would benefit from a clearer quantitative comparison among hydrographs. Please specify whether differences in scour depth arise primarily from peak magnitude, duration of high flow, cumulative shear stress, or interaction with bend-induced secondary currents.

Reply 7:

Thank you for your valuable comment. To clarify the issue, the following paragraph was added to the manuscript in Section 3-2, Results and Discussion. Please see the revised version of the manuscript.

“Given that the peak discharge was kept the same for all four hydrographs, the observed differences in scour depth cannot be attributed to the magnitude of the peak discharge. The results also show that the hydrograph with a peak-time-to-duration ratio of 1.0 produced the greatest changes in bed topography, whereas the ratio of 0.75 resulted in the least bed-topography change. This indicates that the timing of peak discharge played an important role in the development of scour and bed deposition.

Moreover, because secondary flow in the upstream curved reach of the pipe was present in all examined cases, this flow pattern was observed in every configuration, and its effect on downstream bed-topography change was evident under the same geometric bend conditions. Likewise, the cumulative shear stresses associated with flow impingement on the pipe were present to the same extent in all pipe-installation conditions. Therefore, only the effect of hydrograph peak timing, under the three pipe-positioning conditions relative to the initial bed, was considered influential and was examined here.”

Comment 8:

The literature review needs to be strengthened, and incorporating additional recent studies on sediment and flow management in engineered waterways could be beneficial. In this regard, considering studies like, designing a Multistage Flood Control Channel for Sediment and Flow Management in Coastal Areas by Mananoma et al., could help better position the present work within current research trends on integrated hydraulic design and sediment control in complex flow environments.

Reply 8:

With thanks and respect, the literature review has been updated and strengthened, and the following papers have been added to the manuscript in the revised version. In addition, the explanatory paragraph below has been placed before the presentation of the studies.

“Flows, in terms of temporal variability, are classified into steady and unsteady types. Given the temporal variations in flow depth and velocity inherent to the unsteady flow type, the present study focused on examining this class of flows. Accordingly, previous studies and published works related to the behavior of unsteady flows and their effects on flood management and bed changes are reviewed in the following sections.”

Wang L, Cuthbertson A, Pender G, Zhong D. Bed load sediment transport and morphological evolution in a degrading uniform sediment channel under unsteady flow hydrographs. Water Resources Research. 2019;55(7), 5431-5452.‏ http://dx.doi.org/10.1029/2018WR024413

“Cahyani et al. (2022) A study was conducted on the Cengkareng Floodway to investigate the impact of sediment transport on bed morphological changes. Utilizing a Quasi-Unsteady Flow model within the HEC-RAS 1D software and accounting for tidal boundary conditions, the researchers simulated the sedimentation rates [9]. “

Cahyani R, Cahyono M, Eliasta L, Setiadi A, Moerwanto E. Analysis of The Effect Of Sediment Transport on River Bed Changes of Cengkareng Floodway. 2022.

“Mananoma et al. (2025) Regarding the management of flood flows and sediment transport, the researchers designed a multi-stage channel profile using HEC-HMS and HEC-RAS models to control flooding and mitigate the adverse impacts of sediment movement [11].”

Mananoma T, Sumarauw J, Legrans R, Ratu Y. Designing a multistage flood control channel for sediment and flow management in coastal areas. J. Hum. Earth Future. 2025;6(3), 654-662.‏ http://dx.doi.org/10.28991/HEF-2025-06-03-011

Comment 9:

Measurement of bed topography using a laser distance meter with 5 cm by 2.5 cm grid spacing requires an uncertainty assessment. Reporting instrument accuracy, calibration procedures, and estimated measurement error would allow readers to evaluate the reliability of reported maximum scour depths and sedimentation volumes.

Reply 9:

Thank you for your valuable comment. The following paragraph was added to Section 2.4 of the Materials and Methods section to provide a more detailed explanation. Please see the revised version of the manuscript.

“The bed topography was measured using a laser distance meter with an accuracy of 1 mm. The laser meter was mounted on a movable rail system, which allowed systematic displacement of the instrument along the longitudinal and transverse directions of the channel. For data acquisition, the bed was discretized into a grid with transverse spacing of 2.5 cm and longitudinal spacing of 5 cm. In addition, in the vicinity of the pipe and at locations where the topographic variations were more pronounced, the longitudinal spacing was reduced to 2.5 cm or less. To calibrate the measurements and ensure their accuracy before the start of each experiment, the initially flat and leveled channel bed was taken as the reference surface, and the distance from the laser meter to the initial bed surface was taken as the zero-reference level. After each test, the vertical distance between the laser meter and the bed surface was recorded at each grid point, and bed topography changes were calculated based on the difference from the initial zero-reference level. To avoid light refraction effects, all measurements were performed after complete drainage of the bed. In addition, measurements were repeated at several points in each experiment to ensure the repeatability of the measuring locations.”

Comment 10:

Preparation of the initial bed before each run is described as mechanical leveling, but it is unclear whether repeatability tests were conducted. Information on pre-wetting, consolidation time, and verification of identical starting conditions would strengthen the validity of comparisons among experiments.

Reply 10:

We are grateful for your valuable comments. In each pipe-installation condition, the experiment was repeated once, and the observations indicated good repeatability of the tests, such that the locations and magnitudes of scour and deposition were nearly identical. In addition, the method of bed leveling was explained in response to the previous comments, and a paragraph was added to the revised manuscript. Furthermore, the pre-wetting conditions were kept identical in all experiments. In other words, all tests were started at 8:00 a.m. on the day following the previous experiment; therefore, the initial moisture conditions of the bed were the same for all tests. In the first experiment, this condition was also ensured by allowing the flow to run on the day before the test. To clarify the issue, the following paragraph was also added to the revised manuscript in Section 2.3 of the Materials and Methods section:

“In all experiments, by keeping the start time of each test consistent relative to the previous test, the pre-wetting conditions of the bed were identical.”

Comment 11:

The conclusion that pipe position has a stronger effect on scour reduction than unsteady-flow parameters is important but appears largely descriptive. A more systematic sensitivity or statistical analysis would provide stronger support for this claim and help quantify the relative contributions of geometric and hydraulic factors.

Reply 11:

Thank you for your valuable comment. A new subsection 3.5, entitled “Statistical analysis”, has been added at the end of the Results section, which includes four figures (Figs 12–15), and the corresponding descriptive paragraphs for these figures have been incorporated into the revised version of the manuscript.

“Fig 12 presents the effect of hydrograph shape on the maximum scour downstream of the pipe installed in the downstream straight reach of the bend. For the hydrograph with a peak time equal to the base time, the maximum scour depth exceeds the mean value across the three pipe installation configurations relative to the initial bed level. In this unsteady‑flow hydrograph, the longer duration of high discharges during the approach to peak, together with the interaction between the pipe‑induced vortices and the bend‑induced helical flow, intensifies bed shear stress and increases the maximum scour depth to values higher than the mean for each installation condition. In contrast, for the hydrograph with a peak time equal to 75% of the base time—which represents a flood with a delayed peak discharge relative to the normal case—the maximum scour depth in all three pipe installation configurations is lower than the corresponding mean value, due to sediment infilling within the scour hole. In other words, for this ratio of rising to falling limb, before the downstream vortices around the pipe can enlarge the scour hole to its maximum depth, the flow enters the falling limb, thereby limiting the scour development and reducing the maximum depth relative to the mean value. When the peak time is equal to 50% of the base time, the simultaneous effects of sediment exiting the downstream scour hole and sediment collapsing into the hole from upstream cause the maximum scour depth to deviate from the mean value in a configuration‑dependent manner. Finally, when the peak time is equal to 25% of the base time, because the peak occurs early in the experiment, the maximum scour depth in all three pipe installation configurations relative to the initial bed level is greater than the mean value.

In the level‑with‑bed pipe installation condition, which represents the most critical installation configuration among the tested cases, the presence of the pipe as a direct obstacle to the flow results in the highest maximum scour in all four hydrographs compared with the other installation conditions. However, in this pipe installation condition, the direct interaction between the bend's outflow and the pipe structure, through its effect on flow velocity, tends to bring the downstream scour around the pipe to an approximately equilibrium state. Therefore, variations in the hydrograph peak time have a smaller influence on the maximum scour in this installation condition than in the other configurations; as the hydrograph transitions from the rising to the falling limb, no pronounced or significant change in the maximum scour magnitude is observed.

Figure 12. Influence of the hydrograph rising‑to‑falling limb ratio on maximum scour for different pipe installation conditions.

Fig 13 presents the effect of hydrograph shape on the maximum sedimentation downstream of the pipe installed in the downstream straight reach of the bend. The results show that, for all three pipe installation configurations relative to the initial bed level, the hydrograph with a peak time equal to the base time produces a maximum sedimentation greater than the mean value, whereas for hydrographs with peak‑time ratios of 0.25 and 0.75, the maximum sedimentation is lower than the corresponding mean value. In hydrographs whose falling limb has a gradual slope, the flow has sufficient time to continuously redistribute sediments along the reach; in other words, during the falling limb, the flow transports sediments from accumulation zones toward the downstream and prevents excessive deposition at a single location. By contrast, in the hydrograph with a peak time equal to the base time, the absence of a developed falling limb and the abrupt cessation of flow immediately after reaching the peak discharge suppress the continued sediment redistribution process. Consequently, all particles transported during the peak‑discharge period are deposited in place without the opportunity for further movement, leading to maximum sedimentation values higher than those of the other hydrographs.

In the analysis of the effect of hydrograph shape on maximum sedimentation, it was found that, unlike the scour response, changes in hydrograph shape under the level‑with‑bed pipe installation condition exert a pronounced influence on the maximum sedimentation. This is because, in contrast to scour, the sedimentation process is controlled by how the discharge decays over time, which determines whether sufficient time is available for particle settling. In the level‑with‑bed condition, where the pipe is located precisely at the interface between the flow and the bed, even small hydrograph‑induced variations in flow velocity lead to substantial fluctuations in the sedimentation magnitude. By contrast, the sensitivity of the above‑bed and buried installation conditions to changes in hydrograph shape is lower, and the impact on sedimentation is more limited. In the above‑bed installation, the pipe is offset from the bed surface, and in the buried installation, the flow interacts only indirectly with the pipe; in both cases, the turbulence generated by the pipe has a local character and its influence on the overall sedimentation process is reduced, causing the temporal changes in the hydrograph to have a markedly weaker effect on maximum sedimentation.

Figure 13. Influence of the hydrograph rising‑to‑falling limb ratio on maximum sedimentation for different pipe installation conditions.

Fig 14 presents the dimensionless maximum scour versus the dimensionless maximum sedimentation for the different pipe installation conditions in the downstream straight reach of the 180° bend. The results indicate that the pipe installation condition relative to the bed level is the primary factor controlling the dominant direction of the process, although under certain specific hydrograph shapes, flow behavior can be substantially modified.

For the above‑bed pipe installations (Ta), most points lie above the bisector line, indicating that the maximum scour process dominates over maximum sedimentation. However, an atypical and exceptional behavior is observed for the Ta‑0.75 hydrograph: in this case, the corresponding point moves below the bisector line, showing that, unlike the other cases, maximum sedimentation dominates over maximum scour. For the level‑with‑bed installations (Tb), for all four hydrographs considered, the points lie above the bisector line, and the placement of the pipe directly in the flow path, together with the stronger turbulence of the bend's outflow, leads to a dominant maximum scour process relative to maximum sedimentation. In the buried installations (Tc), in contrast to the other two conditions, all points lie below the bisector line, reflecting the dominance of maximum sedimentation over maximum scour. In this installation condition, the pipe is located beneath the bed surface and protected from direct flow impact, which promotes the formation of low‑velocity zones around the pipe and results in extensive sediment deposition.

Figure 14. Dimensionless maximum scour versus dimensionless maximum sedimentation for different pipe installation conditions.

To derive a mathematical relationship governing the experimental data, a power‑law equation was fitted to the laboratory results. Owing to the non‑linear form of the equation, linear regression based on the least‑squares method was applied to determine the model coefficients. The resulting empirical relationship provides a tool for predicting the dimensionless scour depth under different pipe installation conditions and hydrograph shapes. Table 2 presents the coefficients of the equation.

"d" _"s Max" /"D" "=α" 〖"(" "T" _"r" /"T" _"D" ")" 〗^"β" (1)

Table 2. Coefficients of Equation 1

c b a Configuration of pipe Coefficients

1.09 1.82 1.56 α

-0.06 0.01 -0.06 β

After obtaining the empirical coefficients for each pipe installation condition, the proposed equation was used to compute scour depths for all experimental cases. To assess the predictive capability of the model, the deviation between the calculated and measured scour depths was quantified using the relative error percentage and illustrated in Fig 15. The scatter of the data points around the bisector line, together with the relative error values computed for each point, demonstrates the performance of the proposed predictive model.”

Figure 15. Ratio of experimental to computed maximum scour for different pipe installation conditions.

Comment 12:

Mechanistic explanation of observed bed changes could be expanded by linking results more explicitly to known bend-flow phenomena such as helical motion, secondary circulation, flow separation, and redistribution of near-bed shear stress around the pipe. Providing velocity field measurements or referencing established theoretical frameworks would enhance the discussion.

Reply 12:

Thank you for your valuable comment. In the revised version of the manuscript, the explanations provided in the Results section have been expanded, and the findings have been interpreted in terms of the underlying physical mechanisms. In this context, the roles of flow evolution and structure, vortex formation, and the interaction between the flow and the pipe—resulting from the different pipe installation conditions—in shaping the scour and sedimentation patterns have been discussed in greater detail. Accordingly, new paragraphs containing additional discussion and analysis have been added to the manuscript. Please refer to the updated version of the manuscript.

However, it should be noted that no three dimensional velocity measurements were performed in this study; therefore, no quantitative statements have been made regarding detailed flow parameters. The primary objective of the present work was to investigate the downstream bed topography changes around the pipe installed at the end of the bend. Qualitative and observational assessments of the flow pattern were carried out during the experiments, and the scour analysis was supported using these general observations together with the hydrodynamic understanding of the bend’s outflow and its interaction with the pipe, but no quantitative flow field modelling or measurement was undertaken.

Reviewer #3:

Investigation of the Effect of Outflow from a Sharp Bend, Influenced by a Pipe Installed at the Bend Exit, on the Scour Pattern in the Downstream Straight Reach under Unsteady Flow Conditions. The manuscript presents an experimental laboratory study on scour and sedimentation patterns around a pipeline installed at the outlet of a 180-degree sharp bend under unsteady flow conditions. The topic falls within the scope of PLOS One, given its relevance to hydraulic engineering, sediment transport, and river morphology. The contents are in favor for researcher and hydraulic engineers. However, the manuscript in its current form is not merit to be published and requires major corrections before it can be considered for publication. The revised version need review.

Dear Reviewer,

Authors would like to appreciate your kindness and thoughtful comments. We have revised the manuscript as suggested per the given comments. All recommendations have been addressed and highlighted in Blue color on the attached revised manuscript. Because some comments were shared by both reviewers, the corresponding revisions have been highlighted in two different colors within the revised manuscript. However, in this response document, each comment has been addressed separately under the respective reviewer's section. Also, the responses to your comments/suggestions are as follows. We would like to take this opportunity to thank you once again for your thorough consideration that will surely improve the manuscript.

Comment 1:

A significant portion of the manuscript appears to be generated by artificial intelligence. This is evident from repetitive phrasing, overly general statements, lack of critical analysis, and unnatural sentence structures throughout the text. The authors must thoroughly rewrite the manuscript in their own words to ensure originality, clarity, and scientific depth.

Reply 1:

Thank you for your valuable perspective. This paper is based on an experimental study, and all data were obtained from the conducted experiments. The manuscript was also written by the authors based on the measured data. Although no artificial intelligence tools were used in the present study, the manuscript was carefully revised once again; repetitive phrases and general statements were removed, and the text was strengthened with more specific details regarding the causes of the observed phenomena and the reported results. Please see the revised version of the manuscript.

Comment 2:

The manuscript lacks clear logical flow, it is some sections containing content that belongs elsewhere. The methodology and results and discussion sections should be seprated and divided in some headings and subheading and numbered so that each part be destigushed clearly. The manuscript lacks a clear logical flow throughout. To improve clarity and readability, the authors should restructure these sections by introducing appropriate headings and subheadings.

Reply 2:

Thank you for your valuable comment. To improve the coherence and readability of the manuscript, the structure of the different sections was revised. The Materials and Methods and Results and Discussion sections were clearly separated and organized using appropriate numbered headings and subheadings. The order of presentation was also revised so that each section addresses its own topic independently and consistently, making the flow of the manuscript clearer and easier for readers to follow. Please see the revised version of the manuscript.

Accordingly, the following subheadings were added to the manuscript:

In the Materials and Methods section:

1. 2.1 Description of the setup

2. 2.2 Description of the bed and pipe installation

3. 2.3 Experimental procedure

4. 2.4 Bed measurement method

5. 2.5 Test naming convention

In the Results and Discussion section:

1. 3.1 Investigation of the maximum scour and sedimentation points

2. 3.2 Bed topography analysis

3. 3.3 Cross-sectional bed profiles

4. 3.4 Longitudinal bed profiles

5. 3.5 Statistical analysis

Comment 3:

The results are presented only as tables and graphs with basic descriptions, but there is no deep analysis or interpretation of the findings. A dedicated discussion of results is missing. Please provide a thorough discussion that interprets the results, compares them with previous research, explains the physical mechanisms, and highlights the implications of the study.

Reply 3:

Thank you for your valuable comment. In the revised version of the manuscript, the Results and Discussion section has been substantially revised and expanded. The analyses and interpretations of the tables and figures have been further elaborated in the text, the relevant physical mechanisms have been discussed in more detail, and the obtained results have been compared with previous studies in order to clarify the observed similarities and differences. Accordingly, the following paragraphs have been added to Section 3.1 of the Results and Discussion. Please refer to the updated version of the manuscript.

“In 70% of the experiments, the maximum transverse scour depth occurred adjacent to the outer bank of the channel, which is consistent with the findings of Vaghefi et al. (2016) for a 180-degree sharp bend. They stated that, with the development of vortices induced by secondary flow and the gradual shift of the velocity core and bed shear stress concentration along the bend, the dominant zone of shear stress concentration in the downstream end of the bend shifts from the inner bank toward the outer bank. Therefore, the occurrence of maximum scour in these regions can be attributed to the persistence of the secondary flow exiting the bend in this area, namely the downstream straight reach.”

“The comparison of the present study’s results with previous investigations shows that the mechanism of scour occurrence at a longitudinal position relative to the channel center is modified by the upstream bend of the pipe. The scour behavior observed in this study reveals significant differences in the longitudinal position of maximum scour relative to the pipe center compared with the results of Bijanvand et al. (2021) for a straight reach. In Bijanvand et al., as the pipe burial depth relative to the bed decreased from above-bed to pipe installation level with the bed and, finally, to buried at a depth of D/4, the longitudinal position of maximum scour relative to the pipe center decreased accordingly; in other words, the above-bed installation produced the largest longitudinal distance between the pipe center and the position of maximum scour [20]. By contrast, in the present study, where the pipe was installed downstream of a bend, the pattern changed: the buried configuration produced the greatest longitudinal distance of maximum scour relative to the pipe center, whereas for the pipe installation level with the bed and the above-bed installation, the position of maximum scour occurred at shorter longitudinal distances than in the buried case. This difference in the spatial behavior of the maximum scour position reflects the dominance of bend induced helical and secondary flows over the pipe induced vortex structure, which, in the buried pipe condition, exerts a different influence on the downstream sediment transport pattern.”

“position of maximum sedimentation relative to the pipe center is also influenced by the upstream bend. In Bijanvand et al., conducted on a straight reach, the longitudinal distance of maximum sedimentation from the pipe center continuously decreased as the pipe installation changed from above-bed to pipe installation level with the bed and then to buried at a depth of D/4 [20]. By contrast, in the present study, performed on the straight downstream reach of the bend, the pattern differed: the largest longitudinal distance of maximum sedimentation was observed for the pipe installation level with the bed, followed by the above-bed case, while the buried configuration exhibited the smallest distance. This contrasting behavior can be attributed to the effect of secondary flows and the interaction between the bend's outflow and the cross-flow structure around the transverse pipe at the Bend Exit, which alters sediment transport and deposition patterns relative to a straight reach pipe installation.”

Comment 4:

The abstract is disproportionately focused on results, which occupy more than half of its length, while the methodology is described only in general terms. Also there are too many general statement in the abstract. Structure the abstract in this order: rationale → objective → methodology → key findings. Include quantitative results (e.g., 54% reduction in .. )

Reply 4:

Thank you for your careful and valuable comment. To improve the clarity of the abstract and avoid general statements, the abstract was revised and the following paragraph was added to the methodology portion of the abstract so that the research procedure and experimental conditions are described more clearly. The subject, objective, and key quantitative findings had already been presented in the previous abstract.

“The experiments were conducted for three pipe-installation conditions relative to the bed level, namely level with the bed, above the bed, and buried, with the pipe positioned perpendicular to the flow direction and under four different ratios of hydrograph peak timing to flood duration in unsteady flow.”

Comment 5:

Revise Introduction section to focus on directly relevant literature, organized by theme and publication year. Highlight recent advancements and avoid general statements. End with a clear research objective distinct from the title/abstract. Extent to about 1.5 pages. There are useful papers in aceesjr.com journal to be used.

Reply 5:

Thank you, respected reviewer. The Introduction section was revised in light of your comments and those of the other reviewers. Extra sentences were removed, and the relevant articles recommended by the reviewers were also added. Please see the revised version of the manuscript.

Comment 6:

Insufficient detail regarding the experimental setup, including the dimensions and specifications of the flume, the grid resolution, and measurement techniques, the sediment size and experiments time (justifications), how the flow turbulent diminished and …. .

Reply 6:

Thank you very much for your valuable comments. Information such as the flume dimensions and its characteristics, as well as the sediment particle size, had already been mentioned in the Materials and Methods section. The duration of each experiment was 100 minutes and was selected based on previous studies on scour around hydraulic structures, which are also cited in the manuscript. To provide a more precise and transparent explanation of the points you raised, the following paragraphs were added to Sections 2.1, 2.3, and 2.4 of the Materials and Methods, respectively. Please see the revised version of the manuscript.

“To reduce flow turbulence and to homogenize the inflow to the channel, a flow straightener was installed at the channel inlet. This device consisted of two frames made of multilayer fine-mesh aluminum screens placed in the path of the flow discharged from the pump. As a result, the flow entered the straight upstream reach of the channel with reduced turbulence, and after passing through this straight reach with minimum disturbance and turbulence, it entered the bend. Along the upstream bend, the flow conditions changed due to the formation of secondary flow, its interaction with the longitudinal flow, and the development of a helical flow pattern.”

“To evaluate the effect of the hydrograph peak time on the scour process around the pipeline, the experiments were conducted with a total duration of 100 minutes and under four different rising‑limb ratios of the hydrograph (0.25, 0.50, 0.75, and 1.00 times the total duration), based on previous studies on scour around hydraulic structures [8,10].”

“In all four hydrographs used in these experiments, the base discharge, peak discharge, and duration time were kept constant, and the only variable parameter was the timing of the peak discharge relative to the duration time. This parameter was defined by four different ratios in order to investigate the role of the timing of the peak discharge relative to flood duration in the scour and deposition process. In the hydrograph with Tr/Td=0.25, the discharge reaches its peak during the first quarter of the experiment and then gradually returns to the base discharge, representing a flood with a sudden peak under real conditions. In the hydrograph with Tr/Td=0.50, the rising and falling limbs are equal, and the peak discharge occurs at mid-duration, representing an approximately normal flood. In the hydrograph with Tr/Td=0.75, the discharge reaches its peak after three quarters of the experimental duration and then returns to the base discharge, representing a gradual flood with a delayed peak. In the hydrograph with Tr/Td=1.0, the discharge increases gradually from the base discharge to the peak discharge, representing the rise of rainfall to its maximum followed by its abrupt cessation.”

“The bed topography was measured using a laser distance meter with an accuracy of 1 mm. The laser meter was mounted on a movable rail system, which allowed systematic displacement of the instrument along the longitudinal and transverse directions of the channel. For data acquisition, the bed was discretized into a grid with transverse spacing of 2.5 cm and longitudinal spacing of 5 cm. In addition, in the vicinity of the pipe and at locations where the topographic variations were more pronounced, the longitudinal spacing was reduced to 2.5 cm or less. To calibrate the measurements and ensure their accuracy before the start of each experiment, the initially flat and leveled channel bed was taken as the reference surface, and the distance from the laser meter to the initial bed surface was taken as the zero-reference level. After each test, the vertical distance between the laser meter and the bed surface was recorded at each grid point, and bed topography changes were calculated based on the difference from the initial zero-reference level. To avoid light refraction effects, all measurements were performed after complete drainage of the bed. In addition, measurements were repeated at several points in each experiment to ensure the repeatability of the measuring locations.”

Comment 7:

The rationale for selecting specific hydrograph parameters (peak discharge time ratios of 0.25, 0.50, 0.75, 1.00) is not justified.

Reply 7:

Thank you for your valuable comment. To clarify the issue raised, the following paragraph was added to the manuscript in Section 2-3, Materials and Methods. Please see the revised version of the paper.

“In all four hydrographs used in these experiments, the base discharge, peak discharge, and duration time were kept constant, and the only variable parameter was the timing of the peak discharge relative to the duration time. This parameter was defined by four different ratios in order to investigate the role of the timing of the peak discharge relative to flood duration in the scour and deposition process. In the hydrograph with Tr/Td=0.25, the discharge reaches its peak during the first quarter of the experiment and then gradually returns to the base discharge, representing a flood with a sudden peak under real conditions. In the hydrograph with Tr/Td=0.50, the rising and falling limbs are equal, and the peak discharge occurs at mid-duration, representing an approximately normal flood. In the hydrograph with Tr/Td=0.75, the discharge reaches its peak after three quarters of the experimental duration and then returns to the base discharge, representing a gradual flood with a delayed peak. In the hydrograph with Tr/Td=1.0, the discharge increases gradually from the base discharge to the peak discharge, representing the rise of rainfall to its maximum followed by its abrupt cessation.”

Comment 8:

The sediment properties and their critical shear stress values should be provided in more detail. Or stat the flow regime with proper description in the case of clear water and live bed condition, sediment uniformity, pipe installation and any useful details for readers. As mention earlier, The content in the Materials and Methods section should be clearly separated and numbered. Each part must be distinct and presented under appropriate subheadings with proper numbering.

Reply 8:

Thank you for your valuable comment. To clarify the issues raised, the following paragraphs were added to the Materials and Methods section under Subsections 2.2 and 2.3. In addition, as mentioned in our responses to the previous comments, new subheadings were introduced in the relevant sections of the manuscript. With the combination of the original content and the newly added explanations, the overall clarity and readability of the paper have been improved. Please see the revised version of the manuscript.

“The experiments were conducted under unsteady flow conditions, with the discharge gradually altered within the range of 25 to 60 L/s. The water height in the flume was regulated using the tailgate, whose position was adjusted such that for discharge rates lower than 42.5 L/s, the bed shear stress values remained below the critical value, thereby ensuring clear-water conditions. At a discharge rate of 42.5 L/s, the incipient motion of the sediment particles was reached. With further increases in discharge, the bed transitioned into a live-bed condition, in which scour, sediment transport, and sedimentation processes occurred simultaneously.”

“According to Melville and Chiew (1987), if the standard deviation of the bed material particles is less than 1.3, the effect of particle non-uniformity on scour development is negligible. In the present study, the standard deviation of the bed particles was 1.14. Therefore, these materials fall within a uniformity range in which armoring and selective transport effects are minimized.”

“Before each experiment, the pipe was positioned at the desired distance from the bed, perpendicular to the flow direction and across the channel width. To ensure a constant pipe clearance from the initial bed, a level gauge was used. After verifying the correct alignment, the pipe was fixed to both channel walls using transparent aquarium sealant. The sealant was applied carefully so as not to induce flow distortion or additional roughness effects, and thus to prevent any wall-induced deflection of the flow. Throughout the experiments, the pipe remained completely stationary, with no vibration or displacement, and behaved as a rigid body oriented perpendicular to the flow. In addition, a mechanical bed-leveling device was used to prepare the initial bed. This device was mounted on rails installed along the top of the channel walls, with the same slope as the longitudinal bed slope of the channel. At the start of each test, the device was moved along the channel to produce an initial bed with a uniform longitudinal slope.”

Comment 9:

Include sufficient discussion of results in the "Results and Discussion" section in proper places in the text. Use prior studies to validate or justify your findings.

Reply 9:

Thank you for your valuable comment. To provide sufficient explanations regarding the reported findings and to incorporate previous studies and compare them with the present research, the following paragraphs have been added to the Results and Discussion section of the manuscript. Please refer to the updated version of the paper.

“Analysis of the bed topography changes downstream of the 180° bend in the presence of the pipe shows that installing the pipe perpendicular to the flow direction leads to a distinct pattern of bed deformation. In all TR/TD ratios and pipe installation configurations, the bed changes at the end of the experiment evolved such that, with progression towards the downstream of the pipe, the major part of the bed deformation was concentrated along the left bank of the channel. This phenomenon can be attributed to the nature of secondary flow in bends, where velocity vectors near the water surface are driven towards the outer bank and, upon impinging on this bank, intensify scour and sediment transport in that region. This behavior al pattern is in close agreement with the observations of Valizadeh et al. (2023) for semi-buried pipes in a mild 90° bend [23]. The consistency between the present findings and those reported by Valizadeh et al. for a mild bend indicates that, even when the relative bend curvature radius changes from mild to sharp, the primary mechanism of scour concentration along the outer bank for pipes perpendicular to the flow remains the dominant driver of bed morphology adjustment.”

“the most pronounced bed topography changes occurred when the pipe was installed level with the bed. In this configuration, the interaction between the secondary flow exiting the upstream bend and its direct impact on the pipe near the outer bank of the channel, through modification of the bed shear stress around the pipe and changes in flow velocity, caused the extent of the scour and sedimentation zones concentrated along the left bank to become larger than in the other pipe installation configurations.”

“In contrast, for all pipe installation configurations and hydrographs considered, the smallest bed topography changes in terms of both scour and sedimentation were observed when the pipe was installed in the buried condition. In this pipe installation configuration, because the pipe is not directly exposed to the flow during the initial stages of the experiment, its influence on the flow field is weaker than in the other configurations. However, due to the bend upstream of the pipe and the stronger concentration of incoming helical flows towards the straight reach along the left bank compared with the right bank, sediments in this region gradually move over the pipe during the experiment. The removal of the sediment cover from the pipe surface leads to direct contact between the flow and the pipe, thereby modifying flow direction and velocity and ultimately changing the bed topography by forming a local scour hole around the pipe and advancing the eroded sediments towards the downstream of the pipe during the remaining experiment time in the buried condition. In the buried configuration, flow alterations due to pipe–flow contact are weaker than in the configurations where the pipe is exposed to the flow throughout the experiment, so the flow does not have sufficient capacity to transport sediments to more distant areas. Consequently, bed changes in the buried condition are smaller than in the other pipe installation configurations because of the reduced obstruction effect of the pipe.”

“For the pipe installed above the bed, the extent of bed topography variations lay between the other two configurations, such that the scour and sedimentation zones were more pronounced than those of the buried condition but less extensive than those of the level-with-bed condition. The direct presence of the pipe as an obstacle to the flow and the contact between the flow and the pipe in this configuration also produce greater bed topography changes than in the buried condition, due to the resulting modifications in the flow structure. However, because part of the flow passes beneath the pipe, the shear generated around the pipe is lower than in the pipe installation level with the bed condition, and consequently both the intensity and spatial extent of the bed topography changes are smaller than in the pipe installation level with the bed configuration.”

“Thus, it may be inferred that changing the pipe installation condition from level with the bed, which produced the greatest bed topography changes, to the buried condition, which produced the smallest bed topography changes, led, on average, to a 37.1% reduction in the scour hole around the pipe and a 38.0% reduction in sedimentation volume downstream of the pipe.”

“A comparison of Figs 6 a and 7 a shows that the size of the downstream scour hole near the left bank is larger for the level with bed installation than for the above bed installation. This difference is attributed to the stronger flow turbulence in the pipe installation level with the bed condition compared with the above bed installation.”

“Fig 8 illustrates the transverse bed profiles of the straight reach downstream of the bend for the buried pipe condition. The examinations indicated that, similar to the other installation configurations, scour in this case also occurred in the vicinity of the left bank due to the bend's outflow and the concentration of flow near this side of the channel. Moreover, for all four Tr/Td ratios and across all cross sections, bed changes under this pipe installation condition from the inner bank to approximately the channel mid width were relatively constant and stable, and were predominantly characterized by sedimentation. Further away from the pipe location toward the downstream along the channel, as the flow fully entered the straight downstream reach and the influence of the pipe on flow turbulence diminished at larger distances, the magnitude of scour along the left bank progressively decreased. A comparison of the cross sections corresponding to different pipe installation conditions relative to the initial bed level indicated that, in the buried configuration, sedimentation and scour attained their minimum magnitudes due to the protection of the pipe from direct flow impingement during the experimental period and the negligible influence of the buried pipe on the overall flow structure. In contrast, the pipe installation level with the bed exhibited the maximum sedimentation and the greatest scour depth. In addition, in all experiments, the effect of the ratio of peak discharge time to flooding duration on variations in bed scour and sedimentation in the downstream straight reach of the bend was relatively minor when compared with the influence of the pipe installation condition.”

“Figs 9-11 present longitudinal bed profiles of the downstream straight reach of the 180 degree bend in the presence of the pipeline, for three installation configurations relative to the initial bed level at sections located at 5%, 50%, and 95% of the channel width. Based on Figure 9, which depicts the above bed pipe installation, the longitudinal profile at the 5% width section from the right bank of the channel shows that a relative reduction in flow velocity in this region, due to the persistence of bend induced helical flow towards the left bank along part of the downstream bed, led to the dominance of sedimentation processes and the formation of a stable sedimentation pattern along the reach near the right bank. Moreover, at the 50% width section in the channel center, the presence of the pipe and its influence on flow velocity increased bed shear stresses in the vicinity of the pipe, resulting in local scouring; further downstream, with gradual energy dissipation, sediment transport capacity decreased and sedimentation became dominant at intermediate distances, while the weakening of bend induced helical flow and the more uniform distribution of velocity caused bed changes to occur more smoothly. At the 95% width section near the left bank, the persistence of secondary flow energy exiting the bend and its interaction with the pipe led to scour extending over a longer downstream distance from the pipe location; with the gradual weakening of the flow farther downstream, conditions became favorable for sedimentation.”

“Fig 10 compares the longitudinal bed profiles for the level with bed installation condition. At the longitudinal section at 5% of the channel width from the right bank, a relative reduction in velocity and bed shear stress, together with the effect of the level with bed pipe acting as an obstacle to the flow, resulted in sediment accumulation at the beginning of the reach, and at greater distances from the pipe location along the channel, sedimentation continued in a more uniform manner. In addition, at the 50% width section in mid channel, the concentration of shear stresses in the vicinity of the pipe led to local scouring, which, as flow energy gradually dissipated downstream, transitioned into a sedimentation region. At the 95% width section, similar to the above bed installation, the persistence the flow exiting the bend caused scour to extend over a longer distance from the pipe location, and with the gradual dissipation of flow energy as the distance from both the bend and the pipe increased, sedimentation became the dominant process along the downstream reach.”

Comment 10:

Avoid placing figures and tables consecutively. First describe the figure/table in the text, discuss its results, and then proceed to the next figure/table (like line 343).

Reply 10:

Thank you for your valuable suggestion. In the revised version, the order of figures and tables has been corrected so that each figure or table is first cited in the text and then its corresponding results and analysis are presented. Please refer to the updated version of the manuscript.

Comment 11:

The discussion section, where present, is underdeveloped. The authors should more comprehensively interpret their findings in the context of previous research, explaining similarities and differences and their implications for engineering practice.

Reply 11:

Thank you for your valuable comment. In the revised version, the Results and Discussion section has been substantially expanded. To provide a more comprehensive interpretation of the findings, the results of this study were compared with and discussed in light of previous studies so that the scientific significance of the findings could be presented more clearly. Since this comment is similar to the previous ones, we believe it had already been addressed in the earlier responses as well. Accordingly, new paragraphs containing additional discussion and analysis have been added to the manuscript. Please refer to the updated version of the paper.

Comment 12:

The section on AI-generated content is particularly concerning; even some tables in the discussion section appear to show signs of artificial generation and must be rewritten by the authors in their own words.

Reply 12:

Thank you for your valuable comment. The authors would like to state that no AI tools were used in preparing any part of this manuscript, including the text, figures, and tables. All data presented in the paper were obtained from the authors’ experimental results, and the figures and tables were prepared based on the laboratory data measured by the authors using Excel and Tecplot software. In addition, all manuscript text, data analysis, discussion of the results, and conclusions were written and developed by the authors.

Comment 13:

Many statements in the results and conclusion sections are general and do not reflect a critical engagement with the data. For instance, the claim that "the installation condition played a decisive role" is not explored in terms of physical processes (e.g., pressure gradients, vortex dynamics and turbulence modulation).

Reply 13:

Thank you for your valuable comment. In the revised version of the manuscript, the explanations provided in the Results section have been expanded, and the findings have been interpreted in terms of the underlying physical mechanisms. In this context, the roles of flow evolution and structure, vortex formation, and the interaction between the flow and the pipe—resulting from the different pipe installation conditions—in shaping the scour and sedimentation patterns have been discussed in greater detail. Please refer to the updated version of the manuscript. However, it should be noted that no three dimensional velocity measurements were performed in this study; therefore, no quantitative statements have been made regarding detailed flow parameters. The primary objective of the present work was to investigate the downstream bed topography changes around the pipe installed at the end of the bend. Qualitative and observational assessments of the flow pattern were carried out during the experiments, and the scour analysis was supported using these general observations together with the hydrodynamic understanding of the bend’s outflow and its interaction with the pipe, but no quantitative flow field modelling or measurement was undertaken.

Comment 14:

The conclusion that hydrograph shape has "less effect than pipe burial depth" is not adequately justified. A comparative analysis of the relative influence of each parameter (e.g., normalized sensitivity or contribution analysis) would strengthen this assertion. As it stands, the conclusion appears to be based solely on the observation that the percentage reductions differ (37% vs. 13%), which may not be statistically meaningful.

Reply 14:

Thank you for your valuable comment. A new subsection 3.5, entitled “Statistical analysis”, has been added at the end of the Results section, which includes four figures (Figs 12–15), and the corresponding descriptive paragraphs for these figures have been incorporated into the revised version of the manuscript.

“Fig 12 presents the effect of hydrograph shape on the maximum scour downstream of the pipe installed in the downstream straight reach of the bend. For the hydrograph with a peak time equal to the base time, the maximum scour depth exceeds the mean value across the three pipe installation configurations relative to the initial bed level. In this unsteady‑flow hydrograph, the longer duration of high discharges during the approach to peak, together with the interaction between the pipe‑induced vortices and the bend‑induced helical flow, intensifies bed shear stress and increases the maximum scour depth to values higher than the mean for each installation condition. In contrast, for the hydrograph with a peak time equal to 75% of the base time—which represents a flood with a delayed peak discharge relative to the normal case—the maximum scour depth in all three pipe installation configurations is lower than the corresponding mean value, due to sediment infilling within the scour hole. In other words, for this ratio of rising to falling limb, before the downstream vortices around the pipe can enlarge the scour hole to its maximum depth, the flow enters the falling limb, thereby limiting the scour development and reducing the maximum depth relative to the mean value. When the peak time is equal to 50% of the base time, the simultaneous effects of sediment exiting the downstream scour hole and sediment collapsing into the hole from upstream cause the maximum scour depth to deviate from the mean value in a configuration‑dependent manner. Finally, when the peak time is equal to 25% of the base time, because the peak occurs early in the experiment, the maximum scour depth in all three pipe installation configurations relative to the initial bed level is greater than the mean value.

In the level‑with‑bed pipe installation condition, which represents the most critical installation configuration among the tested cases, the presence of the pipe as a direct obstacle to the flow results in the highest maximum scour in all four hydrographs compared with the other installation conditions. However, in this pipe installation condition, the direct interaction between the bend's outflow and the pipe structure, through its effect on flow velocity, tends to bring the downstream scour around the pipe to an approximately equilibrium state. Therefore, variations in the hydrograph peak time have a smaller influence on the maximum scour in this installation condition than in the other configurations; as the hydrograph transitions from the rising to the falling limb, no pronounced or significant change in the maximum scour magnitude is observed.

Figure 12. Influence of the hydrograph rising‑to‑falling limb ratio on maximum scour for different pipe installation conditions.

Fig 13 presents the effect of hydrograph shape on the maximum sedimentation downstream of the pipe installed in the downstream straight reach of the bend. The results show that, for all three pipe installation configurations relative to the initial bed level, the hydrograph with a peak time equal to the base time produces a maximum sedimentation greater than the mean value, whereas for hydrographs with peak‑time ratios of 0.25 and 0.75, the maximum sedimentation is lower than the corresponding mean value. In hydrographs whose falling limb has a gradual slope, the flow has sufficient time to continuously redistribute sediments along the reach; in other words, during the falling limb, the flow transports sediments from accumulation zones toward the downstream and prevents excessive deposition at a single location. By contrast, in the hydrograph with a peak time equal to the base time, the absence of a developed falling limb and the abrupt cessation of flow immediately after reaching the peak discharge suppress the continued sediment redistribution process. Consequently, all particles transported during the peak‑discharge period are deposited in place without the opportunity for further movement, leading to maximum sedimentation values higher than those of the other hydrographs.

In the analysis of the effect of hydrograph shape on maximum sedimentation, it was found that, unlike the scour response, changes in hydrograph shape under the level‑with‑bed pipe installation condition exert a pronounced influence on the maximum sedimentation. This is because, in contrast to scour, the sedimentation process is controlled by how the discharge decays over time, which determines whether sufficient time is available for particle settling. In the level‑with‑bed condition, where the pipe is located precisely at the interface between the flow and the bed, even small hydrograph‑induced variations in flow velocity lead to substantial fluctuations in the sedimentation magnitude. By contrast, the sensitivity of the above‑bed and buried installation conditions to changes in hydrograph shape is lower, and the impact on sedimentation is more limited. In the above‑bed installation, the pipe is offset from the bed surface, and in the buried installation, the flow interacts only indirectly with the pipe; in both cases, the turbulence generated by the pipe has a local character and its influence on the overall sedimentation process is reduced, causing the temporal changes in the hydrograph to have a markedly weaker effect on maximum sedimentation.

Figure 13. Influence of the hydrograph rising‑to‑falling limb ratio on maximum sedimentation for different pipe installation conditions.

Fig 14 presents the dimensionless maximum scour versus the dimensionless maximum sedimentation for the different pipe installation conditions in the downstream straight reach of the 180° bend. The results indicate that the pipe installation condition relative to the bed level is the primary factor controlling the dominant direction of the process, although under certain specific hydrograph shapes, flow behavior can be substantially modified.

For the above‑bed pipe installations (Ta), most points lie above the bisector line, indicating that the maximum scour process dominates over maximum sedimentation. However, an atypical and exceptional behavior is observed for the Ta‑0.75 hydrograph: in this case, the corresponding point moves below the bisector line, showing that, unlike the other cases, maximum sedimentation dominates over maximum scour. For the level‑with‑bed installations (Tb), for all four hydrographs considered, the points lie above the bisector line, and the placement of the pipe directly in the flow path, together with the stronger turbulence of the bend's outflow, leads to a dominant maximum scour process relative to maximum sedimentation. In the buried installations (Tc), in contrast to the other two conditions, all points lie below the bisector line, reflecting the dominance of maximum sedimentation over maximum scour. In this installation condition, the pipe is located beneath the bed surface and protected from direct flow impact, which promotes the formation of low‑velocity zones around the pipe and results in extensive sediment deposition.

Figure 14. Dimensionless maximum scour versus dimensionless maximum sedimentation for different pipe installation conditions.

To derive a mathematical relationship governing the experimental data, a power‑law equation was fitted to the laboratory results. Owing to the non‑linear form of the equation, linear regression based on the least‑squares method was applied to determine the model coefficients. The resulting empirical relationship provides a tool for predicting the dimensionless scour depth under different pipe installation conditions and hydrograph shapes. Table 2 presents the coefficients of the equation.

"d" _"s Max" /"D" "=α" 〖"(" "T" _"r" /"T" _"D" ")" 〗^"β" (1)

Table 2. Coefficients of Equation 1

c b a Configuration of pipe Coefficients

1.09 1.82 1.56 α

-0.06 0.01 -0.06 β

After obtaining the empirical coefficients for each pipe installation condition, the proposed equation was used to compute scour depths for all experimental cases. To assess the predictive capability of the model, the deviation between the calculated and measured scour depths was quantified using the relative error percentage and illustrated in Fig 15. The scatter of the data points around the bisector line, together with the relative error values computed for each point, demonstrates the performance of the proposed predictive model.”

Figure 15. Ratio of experimental to computed maximum scour for different pipe installation conditions.

Comment 15:

The caption is mislabeled. There is no Fig. 5 in text.

Reply 15:

Thank you for your careful observation. Unfortunately, in the previous version of the manuscript, the number of Figure 4 was mistakenly duplicated. This error has been corrected in the revised version, and all figure numbering, in‑text references, and figure captions have been reviewed and made consistent. Please refer to the updated version of the manuscript.

Attachment Submitted filename: renamed_9077d.docx

# Peer review: Decision Letter 1

**DOI:** 10.1371/journal.pone.0360062.r003

**Authors:** Kaywan Othman Ahmed

23 Sep 2026

Investigation of the Effect of Outflow from a Sharp Bend, Influenced by a Pipe Installed at the Bend Exit, on the Scour Pattern in the Downstream Straight Reach under Unsteady Flow Conditions

PONE-D-26-26226R1

Dear Dr. Vaghefi,

We’re pleased to inform you that your manuscript has been judged scientifically suitable for publication and will be formally accepted for publication once it meets all outstanding technical requirements.

Within one week, you’ll receive an e-mail detailing the required amendments. When these have been addressed, you’ll receive a formal acceptance letter and your manuscript will be scheduled for publication.

An invoice will be generated when your article is formally accepted. Please note, if your institution has a publishing partnership with PLOS and your article meets the relevant criteria, all or part of your publication costs will be covered. Please make sure your user information is up-to-date by logging into Editorial Manager at Editorial Manager® and clicking the ‘Update My Information' link at the top of the page. For questions related to billing, please contact billing support.

If your institution or institutions have a press office, please notify them about your upcoming paper to help maximize its impact. If they’ll be preparing press materials, please inform our press team as soon as possible -- no later than 48 hours after receiving the formal acceptance. Your manuscript will remain under strict press embargo until 2 pm Eastern Time on the date of publication. For more information, please contact onepress@plos.org.

Kind regards,

Kaywan Othman Ahmed

Academic Editor

PLOS One

Additional Editor Comments (optional):

Reviewers' comments:

Reviewer's Responses to Questions

**Comments to the Author**

Reviewer #1: (No Response)

Reviewer #2: All comments have been addressed

Reviewer #3: All comments have been addressed

**********

2. Is the manuscript technically sound, and do the data support the conclusions?

Reviewer #1: (No Response)

Reviewer #2: Yes

Reviewer #3: Yes

**********

3. Has the statistical analysis been performed appropriately and rigorously?

Reviewer #1: (No Response)

Reviewer #2: Yes

Reviewer #3: Yes

**********

4. Have the authors made all data underlying the findings in their manuscript fully available?

The PLOS Data policy

Reviewer #1: (No Response)

Reviewer #2: Yes

Reviewer #3: Yes

**********

5. Is the manuscript presented in an intelligible fashion and written in standard English?

Reviewer #1: (No Response)

Reviewer #2: Yes

Reviewer #3: Yes

**********

Reviewer #1: The revised manuscript has been carefully reviewed. The authors have satisfactorily addressed the comments and suggestions raised during the previous review round. The manuscript has been substantially improved in terms of its structure, methodology, literature review, presentation, and discussion of the experimental results.

The revisions have adequately clarified the experimental procedures, research gap, novelty, and practical implications of the findings. Overall, the manuscript is now presented in a clear and satisfactory manner and meets the requirements for publication.

I have no further substantive comments.

Reviewer #2: The authors have successfully addressed the reviewer comments. Well done. Several points to consider to improve quality of this paper.

Reviewer #3: The authors have carefully addressed all of the comments and suggestions raised in my previous review. The manuscript has been substantially improved, particularly in terms of the methodology section, which now includes detailed information on the experimental setup, modeling approach, geometric and hydraulic data, and calibration procedures. The results section has been condensed and refocused on the main findings, and the discussion now provides a more thorough interpretation of the results in the context of previous studies.

In my opinion, the manuscript is now suitable for publication in PLOS One. The study presents valuable experimental data on scour patterns around pipelines under unsteady flow conditions in a sharp bend, and the findings contribute meaningfully to the field of river engineering and hydraulic structures. The paper is well-organized, the methodology is sound, and the conclusions are supported by the data.

I recommend acceptance of the manuscript in its current form.

**********

what does this mean?). If published, this will include your full peer review and any attached files.

If you choose “no”, your identity will remain anonymous but your review may still be made public.

**Do you want your identity to be public for this peer review?** For information about this choice, including consent withdrawal, please see our Privacy Policy

Reviewer #1: **Yes:**  samer abdulhussein

Reviewer #2: No

Reviewer #3: **Yes:**  Ata Amini

**********

# Editor report: Acceptance letter

**DOI:** 10.1371/journal.pone.0360062.r004

**Authors:** Kaywan Othman Ahmed

PONE-D-26-26226R1

PLOS One

Dear Dr. Vaghefi,

I'm pleased to inform you that your manuscript has been deemed suitable for publication in PLOS One. Congratulations! Your manuscript is now being handed over to our production team.

At this stage, our production department will prepare your paper for publication. This includes ensuring the following:

* All references, tables, and figures are properly cited

* All relevant supporting information is included in the manuscript submission,

* There are no issues that prevent the paper from being properly typeset

You will receive further instructions from the production team, including instructions on how to review your proof when it is ready. Please keep in mind that we are working through a large volume of accepted articles, so please give us a few days to review your paper and let you know the next and final steps.

Lastly, if your institution or institutions have a press office, please let them know about your upcoming paper now to help maximize its impact. If they'll be preparing press materials, please inform our press team within the next 48 hours. Your manuscript will remain under strict press embargo until 2 pm Eastern Time on the date of publication. For more information, please contact onepress@plos.org.

You will receive an invoice from PLOS for your publication fee after your manuscript has reached the completed accept phase. If you receive an email requesting payment before acceptance or for any other service, this may be a phishing scheme. Learn how to identify phishing emails and protect your accounts at https://explore.plos.org/phishing.

If we can help with anything else, please email us at customercare@plos.org.

Thank you for submitting your work to PLOS One and supporting open access.

Kind regards,

PLOS One Editorial Office Staff

on behalf of

Dr. Kaywan Othman Ahmed

Academic Editor

PLOS One
